# Copper-catalyzed intermolecular oxyamination of olefins using carboxylic acids and *O*-benzoylhydroxylamines

**DOI:** 10.3762/bjoc.12.4

**Published:** 2016-01-07

**Authors:** Brett N Hemric, Qiu Wang

**Affiliations:** 1 Department of Chemistry, Duke University, Durham, NC, USA

**Keywords:** copper, electrophilic amination, olefin oxyamination

## Abstract

This paper reports a novel approach for the direct and facile synthesis of 1,2-oxyamino moieties via an intermolecular copper-catalyzed oxyamination of olefins. This strategy utilizes *O*-benzoylhydroxylamines as an electrophilic amine source and carboxylic acids as a nucleophilic oxygen source to achieve a modular difunctionalization of olefins. The reaction proceeded in a regioselective manner with moderate to good yields, exhibiting a broad scope of carboxylic acid, amine, and olefin substrates.

## Introduction

**Figure 1:**
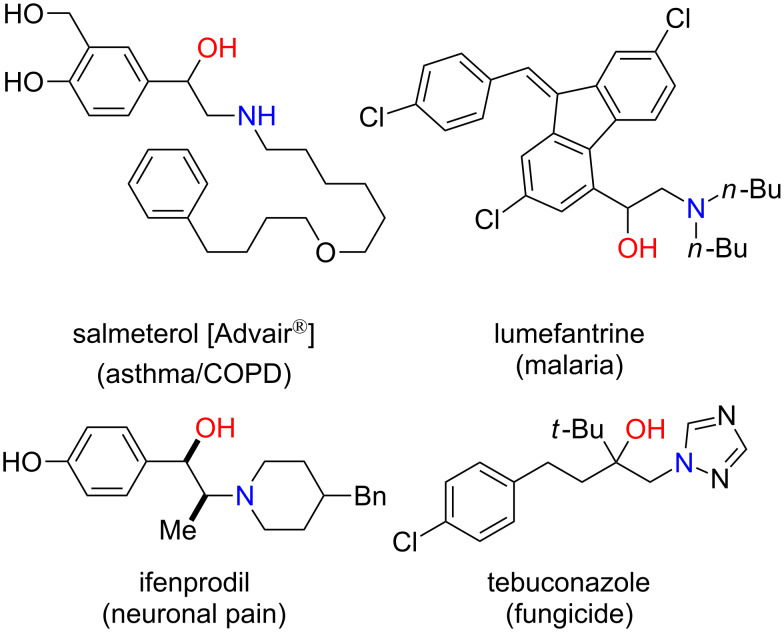
Examples of valuable 1,2-oxyamino-containing molecules.

The 1,2-oxyamino motif is highly valuable and found in a vast range of biologically active natural products, pharmaceuticals, and agrochemicals (Figure 1) [1–2]. Representative examples include salmeterol (Advair^®^), a β_2_-adrenergic receptor agonist [3]; lumefantrine, an antimalarial drug [4]; ifenprodil, an *N*-methyl-D-aspartate (NMDA) antagonist [5]; and tebuconazole, a commercial fungicide [6].

**Scheme 1:**
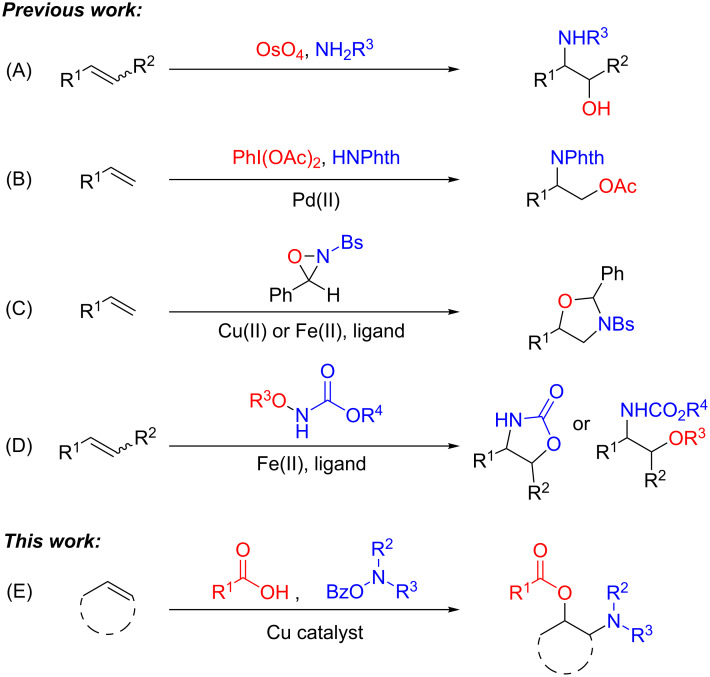
Strategies for intermolecular olefin oxyamination.

With the importance of 1,2-oxyamino motifs as privileged pharmacophores, the development of facile and efficient access to this class of molecules is highly valued. Toward this end, intermolecular olefin oxyamination allows for direct and modular installation of both oxygen and amino groups to readily available olefins in a single step, representing a powerful and appealing approach over multistep sequences [7–9]. Sharpless reported the first examples of this strategy using osmium tetroxide and amines to generate imido osmium intermediates (Scheme 1A) [10–12]. Although this transformation has received extensive application, the development of less toxic olefin oxyamination methods is greatly desired. In 2006, Stahl and Liu reported a palladium-catalyzed introduction of phthalimide (HNPhth) and acetate functionalities to terminal allylic and homoallylic ethers in the presence of an iodine oxidant (Scheme 1B) [13]. The Yoon lab also developed copper- and iron-catalyzed olefin difunctionalization reactions with oxaziridine derivatives to create 1,3-oxazolidines, which were readily converted to the 1,2-oxyamino functionality (Scheme 1C) [14–15]. Recently, the Xu lab developed another iron-catalyzed intermolecular olefin-oxyamination reaction with *O*-alkylhydroxylamides to construct either 1,2-oxyamino or 2-oxazolidinone motifs (Scheme 1D) [16]. Metal-free intermolecular oxyamination reactions have also been accomplished; examples were reported by the Zhu lab with the use of peroxides [17] and by the Studer lab with the use of hypervalent iodo-azide reagents [18]. Furthermore, the intramolecular oxyamination of olefins with a tethered amino or oxygen functionality has been achieved for the construction of a variety of 1,2-oxyamino products, using palladium [19–20], platinum [21], gold [22], copper [23–26], free-radical initiators [27–29], hypervalent iodine [30], and electrochemical oxidation [31–33], as well as an analogous intramolecular electrophilic amino lactonization from our group [34].

Despite these important achievements, current intermolecular olefin oxyamination reactions are often restricted by the use of specific amino and oxygen precursors, limiting the structural diversity of 1,2-oxyamino products. For example, most oxyamination methods are limited to the installation of amide or sulfonamide derivatives. The direct installation of electron-rich amino groups, especially tertiary cyclic amines, remains an unsolved problem. Furthermore, most methods employ inflexible oxygen sources, such as TEMPO and acetate. Herein, we envisioned that a copper-catalyzed intermolecular olefin oxyamination could be achieved using *O*-acylhydroxylamines as an electrophilic amino precursor [35–39] and carboxylic acids as a nucleophilic oxygen source (Scheme 1E). The proposed transformation, integrating an electrophilic amination with a nucleophilic oxygenation, builds upon our recent development in copper-catalyzed olefin difunctionalization, such as copper-catalyzed diamination [40] and amino lactonization [34]. This strategy overcomes common issues of chemo- and regioselectivity associated with adding two different nucleophiles and simultaneously eliminates the need for an external oxidant. Particularly advantageous is the direct addition of electron-rich amino groups, especially tertiary amines, which are difficult to access by other known methods. Furthermore, *O*-acylhydroxylamines are synthetically straightforward and benchtop stable, serving as attractive amine precursors [41–42]. Overall, the development of such a three-component transformation offers an appealing oxyamination strategy complementary to existing methods.

## Results and Discussion

Our investigation began with the use of pentafluorobenzoic acid (**1a**) as the oxygen source and 4-benzoyloxymorpholine (**3a**) as the nitrogen source in the proposed intermolecular oxyamination of styrene (**2a**, Table 1). Copper was found to be effective and critical to promote the formation of oxyamination product **4a** with excellent regioselectivity, as **4a** was not observed in the absence of a catalyst (Table 1, entry 1). Among a variety of copper salts (Table 1, entries 2–7), Cu(OAc)_2_ proved superior as a catalyst (Table 1, entry 2). When the stoichiometry of each reactant was examined for this three-component transformation, decreasing the amount of **1a** resulted in lower yields (Table 1, entry 2 vs 8), while decreasing the amount of **3a** had no effect (Table 1, entry 2 vs 9). Increasing the amount of **2a** led to a noticeable improvement (Table 1, entry 9 vs 12). Finally, the optimal conditions were established with three equivalents of both **1a** and **2a**, providing **4a** in a clean 78% isolation yield (Table 1, entry 13) [43].

**Table 1:** Optimization of copper-catalyzed intermolecular oxyamination.^a^

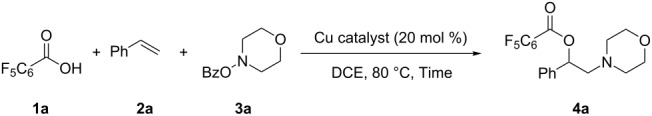						

Entry	Reactants (equiv)			Catalyst	Time^b^	**4a** (%)^c^
	**1a**	**2a**	**3a**			

1	2	1	2	–	24 h	0
2	2	1	2	Cu(OAc)_2_	15 min	63
3	2	1	2	Cu(OTf)_2_	15 min	45
4	2	1	2	CuCl_2_	15 min	60
5	2	1	2	Cu(TFA)_2_	15 min	47
6	2	1	2	Cu(OAc)	15 min	59
7	2	1	2	CuI	60 min	52
8	1	1	2	Cu(OAc)_2_	15 min	48
9	2	1	1	Cu(OAc)_2_	15 min	63
10	3	1	1	Cu(OAc)_2_	15 min	71
11	1	2	2	Cu(OAc)_2_	15 min	77
12	2	2	1	Cu(OAc)_2_	15 min	85
**13**	**3**	**3**	**1**	**Cu(OAc)_2_**	**15 min**	**99 (78)**^d^

^a^Reaction conditions: **1a**, **2a**, **3a**, catalyst (20 mol %), DCE (1.0 mL), 80 °C. ^b^Time required for consumption of **2a**. ^c^Only the indicated isomer was observed. Yields determined by ^1^H NMR spectroscopy with CH_2_Br_2_ as a quantitative internal standard. ^d^Isolated yield. OAc = acetate, OTf = trifluoromethanesulfonate, TFA = trifluoroacetate.

**Scheme 2:**
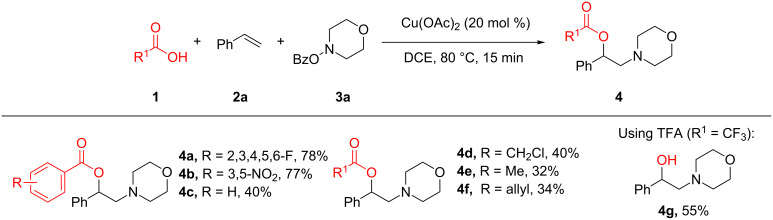
Examples of carboxylic acids in the olefin oxyamination reaction. Reaction conditions: **1** (1.2 mmol, 3.0 equiv), **2a** (3.0 equiv), **3a** (1.0 equiv), Cu(OAc)_2_ (20 mol %), DCE (2.0 mL), 80 °C, 15 min. Isolated yields.

With established oxyamination conditions in hand, the scope of carboxylic acids in this transformation was examined using olefin **2a** and *O*-benzoylhydroxylamine **3a** (Scheme 2). Both aryl and alkyl carboxylic acid derivatives proved to be viable substrates, smoothly providing 1,2-oxyamino products **4a–g**. Carboxylic acids containing a nitro group (**4b**), a halide group (**4d**), or an allyl group (**4f**) were tolerated, demonstrating the broad functional group compatibility of the reaction conditions. It is notable that higher efficiencies were observed in the reactions with more acidic benzoic acids, indicating a correlation of their reactivity to their acidity which remains to be further clarified [44]. Furthermore, when trifluoroacetic acid was employed as an oxygen source, the free 1,2-amino alcohol **4g** was isolated, possibly due to the labile hydrolysis of the trifluoroacetate group.

**Scheme 3:**
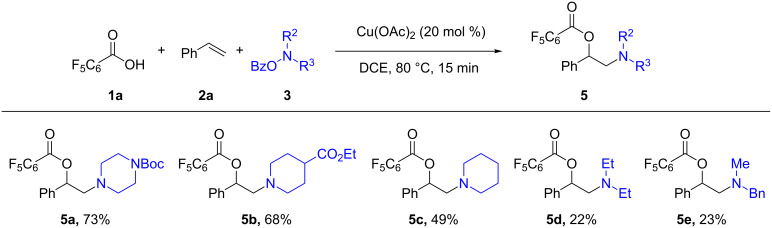
Examples of *O*-benzoylhydroxylamines in the olefin oxyamination reaction. Reaction conditions: **1a** (1.2 mmol, 3.0 equiv), **2a** (3.0 equiv), **3** (1.0 equiv), Cu(OAc)_2_ (20 mol %), DCE (2.0 mL), 80 °C, 15 min. Isolated yields.

Next, the scope of *O*-benzoylhydroxylamines was examined with carboxylic acid **1a** and olefin **2a** (Scheme 3). In the reactions with cyclic *O*-benzoylhydroxylamines, **5a–c** were readily formed in good yields, while acyclic amine products **5d** and **5e** were formed less effectively. Likely, the electronic and steric nature of the acyclic *O*-benzoylhydroxylamines may affect the stability of the corresponding amino intermediates, and result in facile breakdown of the amine precursors to the corresponding free amine. It should be noted that the benzyl group on the amine **5e** can be readily cleaved for the construction of a secondary amine.

Finally, the scope of olefins for the transformation was examined including both aryl and alkyl olefins (Table 2). First, styrene derivatives bearing a variety of substitutions on the aryl ring, including both electron-donating and electron-withdrawing groups, were all effective in providing the 1,2-oxyaminated products with exclusive regioselectivity (Table 2, entries 1–6). Interestingly, the products derived from electron-rich styrene substrates (i.e., **6a**, **6b**, **6e**) were formed in higher yields than those from electron-deficient ones (i.e., **6c** and **6f**). The reactions with simple olefins also provided desired products, yet in poor yields (Table 2, entries 7 and 8). These results indicate that olefin substrates capable of stabilizing a possible electron-deficient intermediate (carbon cation or radical) are likely favored in this transformation. Furthermore, 1,2-disubstituted olefins were also viable substrates (Table 2, entries 9–11). Particularly exciting is the formation of **6j** and **6k** in excellent diastereoselectivity. In contrast, acyclic 1,2-oxyamino product **6i** was formed as a 1:1 mixture of two diastereomers. These results suggest a contribution of the structural conformation to the diastereoselective outcome of the reaction. Yet, 1,1-disubstituted olefins were not effective, likely due to the increased steric hindrance in the oxygenation step (Table 2, entry 12).

**Table 2:** Examples of olefins in intermolecular oxyamination reaction.^a^

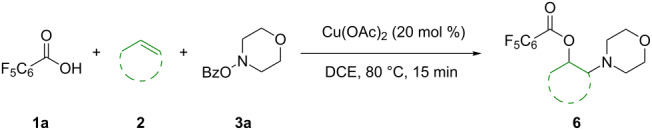				

Entry	Olefin	Product		Yield (%)^b^

1 2 3 4 5 6	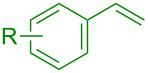	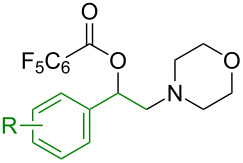	**6a**, R = 2–OMe **6b**, R = 3–OMe **6c**, R = 3–F **6d**, R = 4–Me **6e**, R = 4–OMe **6f**, R = 4–Cl	83 71 57 81 69 70
7 8	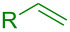	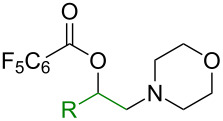	**6g**, R = CH_2_TMS **6h**, R = TMS	19 9
9	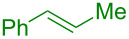	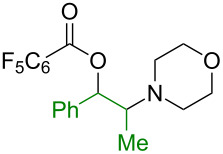	**6i**	69 (1:1 dr)^c^
10	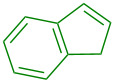	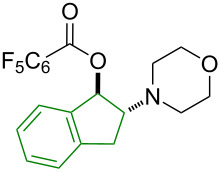	**6j**	89 (7:1 dr)^c^
11	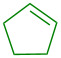	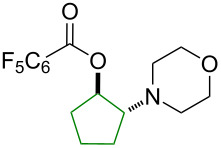	**6k**	35 (>20:1 dr)^c^
12	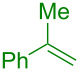	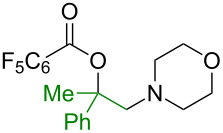	**6l**	<15%^d^

^a^Reaction conditions: **1a** (1.2 mmol, 3.0 equiv), **2** (3.0 equiv), **3a** (1.0 equiv), Cu(OAc)_2_ (20 mol %), DCE (2.0 mL), 80 °C, 15 min. ^b^Isolated yields. ^c^dr = diastereomeric ratio, determined by ^1^H NMR of the crude reaction mixture. Major diastereomer shown. Relative stereochemistry of **6j** determined by X-ray analysis. ^d^Product containing minor inseparable impurities.

Currently, the mechanistic details of this copper-catalyzed oxyamination reaction remain unclear. In the control experiment with the absence of *O*-benzoylhydroxylamine, both carboxylic acid and olefin substrates were fully recovered, suggesting the critical role of *O*-benzoylhydroxylamine in the activation of copper catalyst for the initiation of this reaction. Further investigations are underway for a better understanding of the reaction pathway.

## Conclusion

In summary, we have developed a copper-catalyzed three-component intermolecular oxyamination of olefins using carboxylic acids and *O*-benzoylhydroxylamines. This reaction allows for rapid access to a variety of 1,2-oxyamino products in a modular manner with excellent regioselectivity. It offers an appealing oxyamination method, especially for the construction of electron-rich tertiary amines. Future efforts will be undertaken for a better understanding of the reaction mechanism and for the development of an asymmetric version.

## Supporting Information

File 1 Full experimental details, characterization data and crystallographic data for **6j**.
