# Supplementary material for: Copper-catalyzed intermolecular oxyamination of olefins using carboxylic acids and O-benzoylhydroxylamines
Source: Beilstein J Org Chem. 2016 Jan 7;12:22–8. doi: 10.3762/bjoc.12.4 (PMC4734347; doi:10.3762/bjoc.12.4)
Supplement: File 1 — Full experimental details, characterization data and crystallographic data for 6j. [file Beilstein_J_Org_Chem-12-22-s001.pdf]

Supporting Information

for

Copper-catalyzed intermolecular oxyamination of olefins using carboxylic acids and *O*-benzoylhydroxylamines

Brett N. Hemric and Qiu Wang*

Address: Department of Chemistry, Duke University, Durham, NC

Email: Qiu Wang - qiu.wang@duke.edu

*Corresponding author

Full experimental details, characterization data and crystallographic data for 6j

Table of contents

I. General methods……………………………………………………………………………..….……..S1

II. Condition optimization for oxyamination reaction and experimental protocol……………..……….……...……..S2

III. Characterization data……………..……………………………………………...………...………...…….S6

IV. References………………………………………………………………………………………...….….S16

V. Spectra…………………………………………………………………………………………..……....….S17

VI. X-ray crystallography information………………………………………………………………...…..S68

I. General methods

General Procedures
Glassware and stir bars were dried either with a propane torch or in an oven at 140 ^{o}C overnight and cooled/stored in a dessicator filled with Drierite. Optimization and substrate screens were performed in Chemglass 1 Dram glass vials with Teflon-coated micro stir bar. All other reactions were performed in round-bottom flasks with rubber septa. Plastic syringes were used for the transfer of pure solvents, while glass pipets were used for transfer of crude reaction solutions. Analytical thin-layer chromatography (TLC) was performed using aluminum plates coated with a 0.25 mm layer of 230–400 mesh silica gel with fluorescent indicator (254 nm). TLC plates were visualized by exposure to ultraviolet light and treatment with either vanillin or $KMnO_4$ stain. Organic solutions were concentrated under reduced pressure using a Büchi rotary evaporator and flash chromatography performed using 60 Å silica gel and HPLC-grade solvents.

Materials
Commercial reagents were purchased from Sigma-Aldrich, Alfa Aesar, Acros Organics, Oakwood Chemicals, or Matrix Scientific and used as received. Commercial solvents were obtained exclusively from Sigma-Aldrich and used as received. Dry solvents (Et_2O, CH_2Cl_2, toluene, dioxane, and THF) were obtained from a departmentally-maintained Innovative Technologies solvent purification system. Activated, neutral, Brockmann Grade I (58–60 Å mesh powder).

Instrumentation
Proton and carbon nuclear magnetic resonance (^1H and ^{13}C NMR) spectra were recorded on a Varian INOVA 400 (400 MHz and 100 MHz, respectively) or Bruker 500 (500 MHz and 125 MHz, respectively) spectrophotometer at room temperature unless otherwise noted. Chemical shifts for ^1H NMR are reported in parts per million (ppm, δ) and referenced to residual protium in $CDCl_3$: (δ 7.26). Chemical shifts for ^{13}C NMR are reported in parts per million (ppm, δ) and referenced to the carbon resonances of $CDCl_3$: (δ 77.0). NMR values are reported as follows: chemical shift, multiplicity (s = singlet, d = doublet, t = triplet, q = quartet, quin = quintet, m = multiplet, br = broad), coupling constant (Hz), integration. Infrared spectroscopic data was obtained using a Thermo Scientific Nicolet 380 and reported in wavenumbers (cm^{-1}). High-resolution mass spectra were obtained through the Duke University Mass Spectrometry Facility using an Agilent 1100 Series liquid chromatography-electrospray ionization mass spectrometer.

II. Condition optimization for oxyamination reaction

General experimental procedure for optimization screening

To a 1 Dram vial with Teflon-coated micro stir bar was added pentafluorobenzoic acid (**1a**), 4-benzoyloxymorpholine (**3a**), and copper (II) acetate, followed by addition of anhydrous 1,2-dichloroethane (1.0 mL) and styrene (**2a**). The resulting solution was stirred at 80 °C until consumption of 4-benzoyloxymorpholine **3a** (monitored by TLC). The reaction mixture was cooled down to room temperature and filtered through a plug of activated, neutral Al_2O_3 (Brockman grade I, 58–60Å). The filtrate was concentrated under reduced pressure, providing the crude reaction mixture. Yields were determined by ^{1}H NMR spectroscopy of the crude reaction mixture with 0.1 mmol dibromomethane as quantitative internal standard.

For direct comparison, optimization screening in each table were run and analyzed simultaneously as one batch.

Table S1: Temperature screen[a]

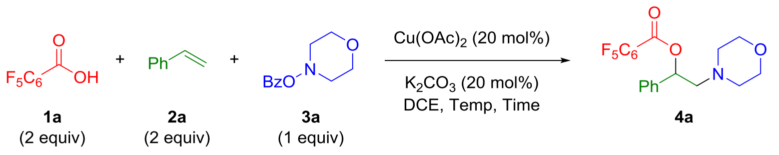

Entry	Temp (°C)	Time (h)	**4**[b] (%)
1	100	0.25	79
**2**	**80**	**0.25**	**84**
3	60	0.75	87
4	40	6.5	76
5	rt	40.75	49

[a]Reaction conditions: **1a** (0.4 mmol), **2a** (0.4 mmol), **3a** (0.2 mmol), $Cu(OAc)_2$ (0.04 mmol), and K_2CO_3 (0.04 mmol) in DCE (1.0 mL). [b]Yield determined by ^{1}H NMR spectroscopy with dibromomethane as a quantitative internal standard.

Table S2: K_2CO_3 Equivalents screen[a]

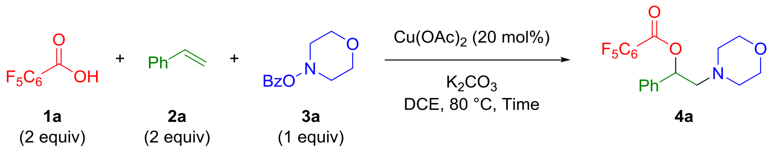

Entry	K_2CO_3 (equiv)	Time (h)	**4**[b] (%)
1	0	0.25	86
2	0.2	0.25	83
3	1.0	0.25	86
4	1.5	0.25	85
5	2.0	0.25	83

[a]Reaction conditions: **1a** (0.4 mmol), **2a** (0.4 mmol), **3a** (0.4 mmol), $Cu(OAc)_2$ (0.04 mmol), and K_2CO_3 in DCE (1.0 mL) at 80 °C. [b]Yield determined by ^{1}H NMR spectroscopy with dibromomethane as a quantitative internal standard.

Table S3: Solvent screen[a]

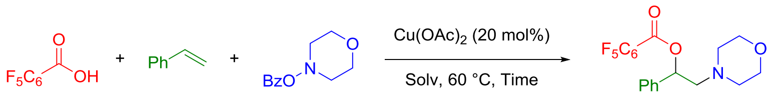

Entry	Solvent [M]	Time (h)	**4**[b] (%)
**1**	**DCE [0.2]**	**0.75**	**85**
2	DBE [0.2]	2.75	71
3	Dioxane [0.2]	2.75	47
4	DME [0.2]	1.25	36
5	Toluene [0.2]	0.75	68
6	CF_3Ph [0.2]	0.75	73
7	CH_3CN [0.2]	22	26
8	DMF [0.2]	22	2
9	EtOH [0.2]	0.75	10

[a]Reaction conditions: **1a** (0.4 mmol), **2a** (0.4 mmol), **3a** (0.2 mmol), and $Cu(OAc)_2$ (0.04 mmol) in solvent (1.0 mL) at 60 °C. [b]Yield determined by ^1H NMR spectroscopy with dibromomethane as a quantitative internal standard.

Table S4: Catalyst screen[a]

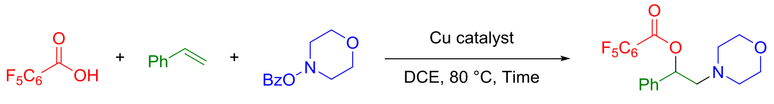

Entry	Catalyst (mol%)	Time (h)	**4**[b] (%)
1	$Cu(OTf)_2$ (20%)	0.25	45
**2**	**$Cu(OAc)_2$ (20%)**	**0.25**	**63**
3	$CuCl_2$ (20%)	0.25	60
4	$Cu(TFA)_2$ (20%)	0.25	47
5	CuF_2 (20%)	0.25	28
6	CuOAc (20%)	0.25	59
7	CuCl (20%)	0.25	65
8	CuI (20%)	1.0	52

[a]Reaction conditions: **1a** (0.4 mmol), **2a** (0.2 mmol), **3a** (0.4 mmol), and Catalyst (0.04 mmol) in DCE (1.0 mL) at 80 °C. [b]Yield determined by ^1H NMR spectroscopy with dibromomethane as a quantitative internal standard.

Table S: Catalyst loading screen[a]

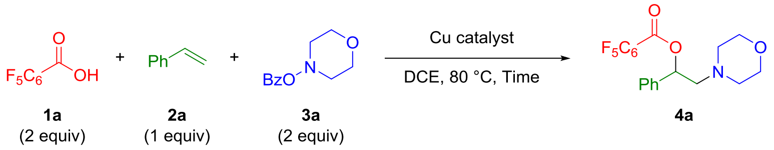

Entry	Catalyst (mol%)	Tim (h)	4[b] (%)
**1**	**$Cu(OAc)_2$ (20%)**	**0.25**	**75**
2	$Cu(OAc)_2$ (10%)	0.25	71
3	---	24	0

[a]Reaction conditions: **1a** (0.4 mmol), **2a** (0.2 mmol), **3a** (0.4 mmol), and $Cu(OAc)_2$ in DCE (1.0 mL) at 80 °C. [b]Yield determined by ^1H NMR spectroscopy with dibromomethane as a quantitative internal standard.

Table S6: Reactant equivalents screen[a]

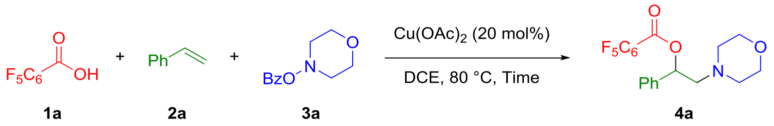

Entry	1a (equiv)	2a (equiv)	3a (equiv)	Time (h)	4[b] (%)
1	1	1	1	2.0	42
2	1	1	2	2.0	48
3	1	1	3	2.0	45
4	2	1	1	0.25	63
5	2	1	2	0.25	67
6	2	1	3	0.25	59
7	3	1	1	0.25	71
8	3	1	2	0.25	75
9	3	1	3	0.25	62
10	1	2	1	0.75	67
11	1	2	2	0.25	77
12	1	2	3	1.5	78
13	2	2	1	0.25	85
14	3	2	1	1.5	88
15	1	3	1	0.5	82
16	1	3	2	1.75	96
17	1	3	3	1.75	99
18	2	3	1	0.25	98
**19**	**3**	**3**	**1**	**0.25**	**99**

[a]Reaction conditions: **1a**, **2a**, **3a**, and $Cu(OAc)_2$ (0.04 mmol) in DCE (1.0 mL) at 80 °C. [b]Yield determined by ^1H NMR spectroscopy with dibromomethane as a quantitative internal standard.

Table S7: 1,1-Disubstituted olefins in the oxyamination reaction[a]

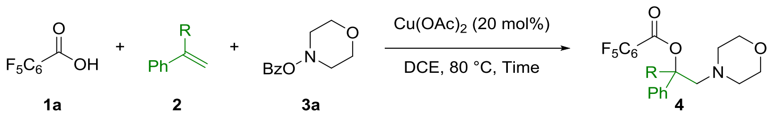

Entry	**2**	Time (h)	**4** (%)
1	R = Me	0.25	<15[b]
2	R = Ph	0.25	ND[c]

[a]Reaction conditions: **1a** (1.2 mmol, 3.0 equiv), **2** (3.0 equiv), **3a** (1.0 equiv), $Cu(OAc)_2$ (20 mol%), DCE (2.0 mL), 80 °C. [b]Contained some inseparable impurities. [c]Not detected by GCMS.

Established standard procedure for olefin oxyamination reaction

To a 1 Dram vial with Teflon-coated micro stir bar was added carboxylic acid **1** (1.2 mmol, 3 equiv), *O*-benzoylhydroxylamine **3** (0.4 mmol, 1 equiv), and copper(II) acetate (0.08 mmol, 0.2 equiv), followed by addition of anhydrous 1,2-dichloroethane (2.0 mL) and olefin **2** (1.2 mmol, 3 equiv). The resulting solution was stirred at 80 °C for 15 min until the consumption of *O*-benzoylhydroxylamine **3** (monitored by TLC). The resulting reaction mixture was cooled to room temperature and filtered through a plug of activated, neutral Al_2O_3 (Brockman grade I, 58–60Å). The filtrate was concentrated under reduced pressure, providing the crude reaction mixture. The crude reaction mixture was purified by silica column chromatography unless otherwise noted.

Preparation of starting materials

All carboxylic acids (**1a**–**f**) and olefins (**2a**–**k**) were purchased from commercial sources. Carboxylic acids were used without further purification. Olefins containing stabilizing agent (such as 4-*tert*-butylcatechol) were flushed neat through a short plug of activated, neutral Al_2O_3 (Brockman grade I, 58–60 Å) before use.

***O*-Benzoylhydroxylamines (3a–e)** were synthesized according to a previously reported method.[1]

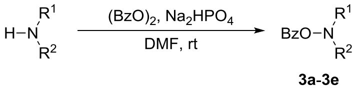

III. Characterization data

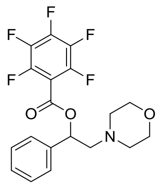

4a

2-Morpholino-1-phenylethyl 2,3,4,5,6-pentafluorobenzoate (4a).

Prepared using standard conditions. Purification by flash column chromatography (100% hexanes → 20% ethyl acetate–hexanes) gave **4a** as a white solid (126.0 mg, 78%).

R_f = 0.70 (50% ethyl acetate–hexanes)

^{1}H NMR ($CDCl_3$, 400 MHz): δ 7.42–7.31 (m, 5H), 6.25 (dd, *J* = 9.8, 3.5 Hz, 1H), 3.71–3.61 (m, 4H), 2.89 (dd, *J* = 13.5, 9.8 Hz, 1H), 2.72–2.60 (m, 3H), 2.50–2.42 (m, 2H)

^{13}C NMR ($CDCl_3$, 125 MHz): δ 158.0, 145.3 (dd, J_{C-F} = 258.1, 4.7 Hz, 2C), 143.0 (dt, J_{C-F} = 259.1, 13.0 Hz, 1C), 137.6 (dt, J_{C-F} = 254.8, 14.1 Hz, 2C), 137.5, 128.4 (2C), 128.3, 126.4 (2C), 108.4 (t, J_{C-F} = 16.1 Hz, 1C), 74.8, 66.9 (2C), 64.1, 53.7 (2C)

FTIR (thin film): cm^{-1} 2811, 1734, 1494, 1325, 1223, 1116, 993, 755, 698

HRMS-ESI (m/z) Calcd for ($C_{19}H_{17}F_5NO_3$) ($[M+H]^+$): 402.1123; found: 402.1124

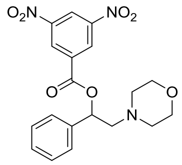

4b

2-Morpholino-1-phenylethyl 3,5-dinitrobenzoate (4b).

Prepared using standard conditions. Purification by flash column chromatography (100% hexanes → 20% ethyl acetate–hexanes) gave **4b** as a yellow foam (124.0 mg, 77%).

R_f = 0.18 (50% ethyl acetate–hexanes)

^{1}H NMR ($CDCl_3$, 400 MHz): δ 9.19 (t, *J* = 2.1 Hz, 1H), 9.16 (d, *J* = 2.1 Hz, 2H), 7.46 (d, *J* = 7.1 Hz, 2H), 7.39 (t, *J* = 7.1 Hz, 2H), 7.34 (d, *J* = 7.1 Hz, 1H), 6.30 (dd, *J* = 9.5, 3.6 Hz, 1H), 3.67–3.57 (m, 4H), 3.08 (dd, *J* = 13.6, 9.5 Hz, 1H), 2.78–2.65 (m, 3H), 2.55–2.47 (m, 2H)

^{13}C NMR ($CDCl_3$, 125 MHz): δ 161.7, 148.5 (2C), 137.5, 134.0, 129.3 (2C), 128.7 (3C), 126.5 (2C), 122.2, 75.0, 66.8 (2C), 63.8, 53.7 (2C)

FTIR (thin film): cm^{-1} 3101, 1728, 1628, 1541, 1454, 1343, 1269, 1164, 1115, 718, 699

HRMS-ESI (m/z) Calcd for ($C_{19}H_{20}N_3O_7$) ($[M+H]^+$): 402.1296; found: 402.1295

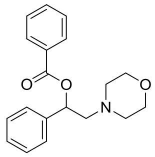

4c

2-Morpholino-1-phenylethyl benzoate (4c).

Prepared using standard conditions. Purification by flash column chromatography (100% hexanes → 20% ethyl acetate–hexanes) gave **4c** as a clear oil (50.3 mg, 40%).

R_f = 0.47 (50% ethyl acetate–hexanes)

^{1}H NMR ($CDCl_3$, 400 MHz): δ 8.11 (d, *J* = 7.1 Hz, 2H), 7.57 (t, *J* = 7.4 Hz, 1H), 7.50–7.42 (m, 4H), 7.40–7.28 (m, 3H), 6.25 (dd, *J* = 8.8, 3.9 Hz, 1H), 3.67 (t, *J* = 4.7 Hz, 4H), 3.04 (dd, *J* = 13.6, 8.8 Hz, 1H), 2.75 (dd, *J* = 13.6, 3.9 Hz, 1H), 2.70–2.63 (m, 2H), 2.61–2.54 (m, 2H)

^{13}C NMR ($CDCl_3$, 125 MHz): δ 165.6, 139.2, 132.9, 130.3, 129.6 (2C), 128.5 (2C), 128.3 (2C), 128.0, 126.4 (2C), 73.4, 66.9 (2C), 64.1, 53.7 (2C)

FTIR (thin film): cm^{-1} 2854, 1716, 1451, 1266, 1112, 710, 700

HRMS-ESI (m/z) Calcd for ($C_{19}H_{22}NO_3$) ([M+H]$^+$): 312.1594; found: 312.1594

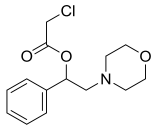

4d

2-Morpholino-1-phenylethyl 2-chloroacetate (4d).

Prepared using standard conditions. Purification by flash column chromatography (100% hexanes → 60% ethyl acetate–hexanes) gave **4d** as a clear oil (45.4 mg, 40%).

R_f = 0.14 (50% ethyl acetate–hexanes)

^{1}H NMR ($CDCl_3$, 400 MHz): δ 7.39–7.29 (m, 5H), 6.05 (dd, *J* = 9.5, 3.6 Hz, 1H), 4.13 (d, *J* = 14.8 Hz, 1H), 4.08 (d, *J* = 14.8, 1H), 3.73–3.63 (m, 4H), 2.88 (dd, *J* = 13.6, 9.5 Hz, 1H), 2.67–2.56 (m, 3H), 2.53–2.45 (m, 2H)

^{13}C NMR ($CDCl_3$, 125 MHz): δ 166.5, 137.6, 128.6 (2C), 128.4, 126.5 (2C), 74.2, 66.8 (2C), 63.8, 53.7 (2C), 41.1

FTIR (thin film): cm^{-1} 2855, 1757, 1454, 1299, 1171, 1115, 1010, 873, 700

HRMS-ESI (m/z) Calcd for ($C_{14}H_{19}ClNO_3$) ([M+H]$^+$): 284.1048; found: 284.1048

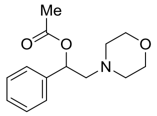

4e

2-Morpholino-1-phenylethyl acetate (4e).

Prepared using standard conditions. Purification by flash column chromatography (100% hexanes → 60% ethyl acetate–hexanes) gave **4e** as a clear oil (31.6 mg, 32%).

R_f = 0.23 (50% ethyl acetate–hexanes)

^{1}H NMR ($CDCl_3$, 400 MHz): δ 7.42–7.27 (m, 5H), 5.97 (dd, *J* = 9.1, 4.0 Hz, 1H), 3.72–3.63 (m, 4H), 2.84 (dd, *J* = 13.4, 9.1 Hz, 1H), 2.63–2.52 (m, 3H), 2.52–2.43 (m, 2H), 2.10 (s, 3H)

^{13}C NMR ($CDCl_3$, 125 MHz): δ 170.1, 139.2, 128.4 (2C), 128.0, 126.5 (2C), 72.4, 66.9 (2C), 64.0, 53.8 (2C), 21.3

FTIR (thin film): cm^{-1} 2853, 1733, 1495, 1454, 1231, 1116, 1027, 872, 700

HRMS-ESI (m/z) Calcd for ($C_{14}H_{20}NO_3$) ([M+H]$^+$): 250.1438; found: 250.1440

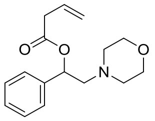

4f

2-Morpholino-1-phenylethyl but-3-enoate (4f).

Prepared using standard conditions. Purification by flash column chromatography (100% hexanes → 30% ethyl acetate–hexanes) gave **4f** as a clear oil (37.8 mg, 34%).

R_f = 0.31 (50% ethyl acetate–hexanes)

^{1}H NMR ($CDCl_3$, 400 MHz): δ 7.38–7.27 (m, 5H), 6.00 (dd, *J* = 9.2, 4.0 Hz, 1H), 5.93 (ddt, *J* = 17.1, 10.3, 6.9 Hz, 1H), 5.22–5.17 (m, 1H), 5.17–5.14 (m, 1H), 3.74–3.62 (m, 4H), 3.18–3.12 (m, 1H), 2.85 (dd, *J* = 13.5, 9.2 Hz, 1H), 2.66–2.54 (m, 3H), 2.53–2.44 (m, 2H)

^{13}C NMR ($CDCl_3$, 125 MHz): δ 170.6, 138.9, 130.2, 128.4 (2C), 128.1, 126.5 (2C), 118.5, 72.6, 66.9 (2C), 64.0, 53.7 (2C), 39.3

FTIR (thin film): cm^{-1} 2854, 1735, 1454, 1168, 1116, 1010, 917, 872, 757, 700

HRMS-ESI (m/z) Calcd for ($C_{16}H_{22}NO_3$) ($[M+H]^+$): 276.1594; found: 276.1594

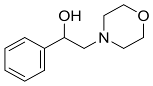

4g

2-Morpholino-1-phenylethan-1-ol (4g).[2,3]

Prepared using standard conditions. Purification by flash column chromatography (100% hexanes → 70% ethyl acetate–hexanes) gave **4g** as a white solid (44.7 mg, 55%).

R_f = 0.14 (50% ethyl acetate–hexanes)

^{1}H NMR ($CDCl_3$, 400 MHz): δ 7.41–7.23 (m, 5H), 4.77 (dd, *J* = 10.1, 3.8 Hz, 1H), 4.23–4.02 (s, broad, 1H), 3.85–3.68 (m, 4H), 2.81–2.69 (m, 2H), 2.58–2.42 (m, 4H)

^{13}C NMR ($CDCl_3$, 125 MHz): δ 141.8, 128.3 (2C), 127.5, 125.7 (2C), 68.5, 66.9 (2C), 66.6, 53.4 (2C)

FTIR (thin film): cm^{-1} 3421, 2812, 1494, 1453, 1115, 1006, 867, 755, 700

HRMS-ESI (m/z) Calcd for ($C_{12}H_{18}NO_2$) ($[M+H]^+$): 208.1332; found: 208.1333

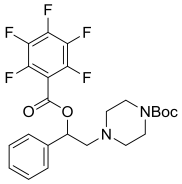

5a

***Tert*-Butyl 4-(2-((perfluorobenzoyl)oxy)-2-phenylethyl)piperazine-1-carboxylate (5a).**

Prepared using standard conditions. Purification by flash column chromatography (100% hexanes → 30% ethyl acetate–hexanes) gave **5a** as a white powder (146.1 mg, 73%).

R_f = 0.81 (50% ethyl acetate–hexanes)

^{1}H NMR ($CDCl_3$, 400 MHz): δ 7.41–7.3 (m, 5H), 6.25 (dd, *J* = 9.8, 3.3 Hz, 1H), 3.44–3.32 (m, 4H), 2.90 (dd, *J* = 13.6, 9.8 Hz, 1H), 2.67–2.59 (m, 3H), 2.45–2.36 (m, 2H), 1.44 (s, 3H)

^{13}C NMR ($CDCl_3$, 125 MHz): δ 158.1, 154.7, 145.3 (d, J_{C-F} = 258.5 Hz, 2C), 143.1 (d, J_{C-F} = 259.4 Hz, 1C), 137.6 (d, J_{C-F} = 254.4 Hz, 2C), 137.4, 128.6 (2C), 128.5, 126.5 (2C), 108.4 (t, J_{C-F} = 16.1 Hz, 1C), 79.5, 75.0, 63.7, 53.0 (2C), 44.0, 43.1, 28.3 (3C)

FTIR (thin film): cm^{-1} 2977, 1737, 1688, 1652, 1523, 1496, 1421, 1326, 1226, 1170, 1001, 947, 752, 698

HRMS-ESI (m/z) Calcd for ($C_{24}H_{26}F_5N_2O_4$) ($[M+H]^+$): 501.1807; found: 501.1808

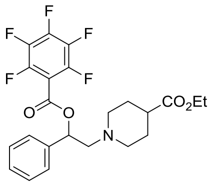

5b

Ethyl 1-(2-((perfluorobenzoyl)oxy)-2-phenylethyl)piperidine-4-carboxylate (5b).

Prepared using standard conditions. Purification by flash column chromatography (100% hexanes → 20% ethyl acetate–hexanes) gave **5b** as a clear oil (127.5 mg, 68%).

R_f = 0.30 (50% ethyl acetate–hexanes)

^1H NMR ($CDCl_3$, 400 MHz): δ 7.43–7.29 (m, 5H), 6.23 (dd, J = 9.5, 3.6 Hz, 1H), 4.12 (q, J = 7.1 Hz, 2H), 3.10–3.02 (m, 1H), 2.94–2.80 (m, 2H), 2.61 (dd, J = 13.7, 3.6 Hz, 1H), 2.32–2.20 (m, 2H), 2.1 (td, J = 11.0, 2.0 Hz, 1H), 1.91–1.81 (m, 2H), 1.77–1.62 (m, 2H), 1.24 (t, J = 7.1 Hz, 3H)

^{13}C NMR ($CDCl_3$, 125 MHz): δ 174.7, 157.9, 145.3 (d, J_{C-F} = 257.7 Hz, 2C), 142.9 (d, J_{C-F} = 258.7 Hz, 1C), 137.5, 137.5 (d, J_{C-F} = 253.9 Hz, 2C), 128.4 (2C), 128.3, 126.3 (2C), 108.4 (t, J_{C-F} = 15.8 Hz, 1C), 75.1, 63.5, 60.1, 53.5, 52.3, 40.5, 28.0, 27.8, 13.9

FTIR (thin film): cm^{-1} 2950, 1730, 1651, 1523, 1496, 1326, 1224, 995, 751, 698

HRMS-ESI (m/z) Calcd for ($C_{23}H_{23}F_5NO_4$) ($[M+H]^+$): 472.1542; found: 472.1540

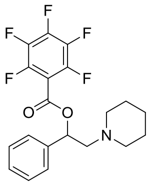

5c

1-Phenyl-2-(piperidin-1-yl)ethyl 2,3,4,5,6-pentafluorobenzoate (5c).

Prepared using standard conditions. Purification by flash column chromatography (100% hexanes → 60% ethyl acetate–hexanes) gave **5c** as a beige powder (77.6 mg, 49%).

R_f = 0.12 (50% ethyl acetate–hexanes)

^1H NMR ($CDCl_3$, 400 MHz): δ 7.45–7.28 (m, 5H), 6.26 (dd, J = 9.6, 3.4 Hz, 1H), 2.89 (dd, J = 13.7, 9.6 Hz, 1H), 2.68–2.54 (m, 3H), 2.48–2.36 (m, 2H), 1.63–1.47 (m, 4H), 1.47–1.33 (m, 2H)

^{13}C NMR ($CDCl_3$, 125 MHz): δ 158.0, 145.4 (d, J_{C-F} = 258.5 Hz, 2C), 143.0 (d, J_{C-F} = 261.0 Hz, 1C), 137.9, 137.6 (d, J_{C-F} = 254.6 Hz, 2C), 128.5 (2C), 128.4, 126.5 (2C), 108.4 (t, J_{C-F} = 15.5 Hz, 1C), 75.3, 64.1, 54.6 (2C), 25.6 (2C), 23.9

FTIR (thin film): cm^{-1} 2936, 1737, 1651, 1523, 1494, 1325, 1225, 993, 946, 756, 698

HRMS-ESI (m/z) Calcd for ($C_{20}H_{19}F_5NO_2$) ($[M+H]^+$): 400.1330; found: 400.1330

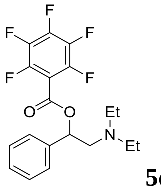

5d

2-(Diethylamino)-1-phenylethyl 2,3,4,5,6-pentafluorobenzoate (5d).

Prepared using standard conditions. Purification by flash column chromatography (100% hexanes → 20% ethyl acetate–hexanes) gave **5d** as a clear oil (34.5 mg, 22%).

R_f = 0.07 (50% ethyl acetate–hexanes)

^{1}H NMR ($CDCl_3$, 400 MHz): δ 7.44–7.30 (m, 5H), 6.13 (dd, *J* = 8.8, 4.2 Hz, 1H), 3.00 (dd, *J* = 14.1, 8.8 Hz, 1H), 2.74 (dd, *J* = 14.1, 4.2 Hz, 1H), 2.71–2.51 (m, 4H), 1.00 (t, *J* = 7.1, Hz)

^{13}C NMR ($CDCl_3$, 125 MHz): δ 158.1, 145.6 (d, J_{C-F} = 254.5 Hz, 2C), 143.1 (d, J_{C-F} = 259.9 Hz, 1C), 137.9, 137.6 (d, J_{C-F} = 250.4 Hz, 2C), 128.6 (2C), 128.5, 126.5 (2C), 108.2 (t, J_{C-F} = 15.2 Hz, 1C), 76.4, 58.4, 47.5 (2C), 11.3 (2C)

FTIR (thin film): cm^{-1} 2972, 1738, 1651, 1524, 1496, 1327, 1226, 999, 945, 699

HRMS-ESI (m/z) Calcd for ($C_{19}H_{19}F_5NO_2$) ($[M+H]^+$): 388.1330; found: 388.1330

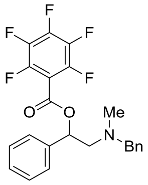

5e

2-(Benzyl(methyl)amino)-1-phenylethyl 2,3,4,5,6-pentafluorobenzoate (5e).

Prepared using standard conditions. Purification by flash column chromatography (100% hexanes → 20% ethyl acetate–hexanes) gave **5e** as a clear oil (40.5 mg, 23%).

R_f = 0.71 (50% ethyl acetate–hexanes)

^{1}H NMR ($CDCl_3$, 400 MHz): δ 7.44–7.31 (m, 5H), 7.30–7.20 (m, 5H), 6.26 (dd, *J* = 8.8, 4.2 Hz, 1H), 3.68 (d, *J* = 13.2 Hz, 1H), 3.58 (d, *J* = 13.2 Hz, 1H), 3.06 (dd, *J* = 13.6, 8.8 Hz, 1H), 2.75 (dd, *J* = 13.6, 4.2 Hz, 1H), 2.36 (s, 3H)

^{13}C NMR ($CDCl_3$, 125 MHz): δ 158.2, 145.6 (d, J_{C-F} = 261.9 Hz, 2C), 143.1 (d, J_{C-F} = 262.3 Hz, 1C), 138.4, 137.9, 137.6 (d, J_{C-F} = 248.9 Hz, 2C), 128.8 (2C), 128.5 (2C), 128.4, 128.1 (2C), 127.0, 126.7 (2C), 108.3 (t, J_{C-F} = 15.2 Hz, 1C), 76.2, 62.4, 62.4, 42.6

FTIR (thin film): cm^{-1} 2798, 1737, 1652, 1523, 1495, 1327, 1227, 998, 698

HRMS-ESI (m/z) Calcd for ($C_{23}H_{19}F_5NO_2$) ($[M+H]^+$): 436.1330; found: 436.1330

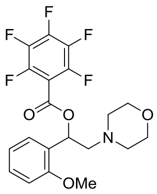

6a

1-(2-Methoxyphenyl)-2-morpholinoethyl 2,3,4,5,6-pentafluorobenzoate (6a).

Prepared using standard conditions. Purification by flash column chromatography (100% hexanes → 20% ethyl acetate–hexanes) gave **6a** as a white solid (143.4 mg, 83%).

R_f = 0.59 (50% ethyl acetate–hexanes)

^{1}H NMR ($CDCl_3$, 400 MHz): δ 7.40 (d, *J* = 7.5 Hz, 1H), 7.30 (t, *J* = 8.0 Hz, 1H), 6.98 (t, *J* = 7.5 Hz, 1H), 6.91 (d, *J* = 8.0 Hz, 1H), 6.72 (dd, *J* = 9.7, 2.5 Hz, 1H), 3.88 (s, 3H), 3.72–3.62 (m, 4H), 2.82–2.71 (m, 3H), 2.64 (dd, *J* = 13.4, 2.5 Hz, 1H), 2.48–2.40 (m, 2H)

^{13}C NMR ($CDCl_3$, 125 MHz): δ 157.8, 155.9, 145.2 (d, $J_{C\text{-}F}$ = 257.4 Hz, 2C), 142.8 (d, $J_{C\text{-}F}$ = 258.2 Hz, 1C), 137.5 (d, $J_{C\text{-}F}$ = 254.1 Hz, 2C), 129.1, 126.3, 125.9, 120.5, 110.3, 108.7 (t, $J_{C\text{-}F}$ = 16.1 Hz, 1C), 69.6, 66.9 (2C), 62.9, 55.2, 53.5 (2C)

FTIR (thin film): cm^{-1} 2810, 1737, 1651, 1523, 1494, 1329, 1222, 1116, 994, 752

HRMS-ESI (m/z) Calcd for ($C_{20}H_{19}F_5NO_4$) ($[M+H]^+$): 432.1229; found: 432.1228

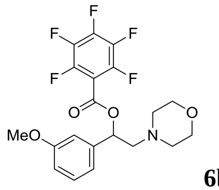

6b

1-(3-Methoxyphenyl)-2-morpholinoethyl 2,3,4,5,6-pentafluorobenzoate (6b).

Prepared using standard conditions. Purification by flash column chromatography (100% hexanes → 20% ethyl acetate–hexanes) gave **6b** as a white solid (121.7 mg, 71%).

R_f = 0.55 (50% ethyl acetate–hexanes)

^{1}H NMR ($CDCl_3$, 400 MHz): δ 7.29 (t, *J* = 8.0 Hz, 1H), 7.00–6.93 (m, 2H), 6.87 (dd, *J* = 8.2, 2.6 Hz, 2H), 6.22 (dd, *J* = 9.7, 3.4 Hz, 1H), 3.82 (s, 3H), 3.70–3.63 (m, 4H), 2.88 (dd, *J* = 13.6, 9.7 Hz, 1H), 2.73–2.60 (m, 3H), 2.50–2.42 (m, 2H)

^{13}C NMR ($CDCl_3$, 125 MHz): δ 159.6, 157.9, 145.3 (d, $J_{C\text{-}F}$ = 266.3 Hz, 2C), 143.0 (d, $J_{C\text{-}F}$ = 259.8 Hz, 1C), 139.0, 137.5 (d, $J_{C\text{-}F}$ = 252.8 Hz, 2C), 129.5, 118.6, 113.6, 112.0, 108.3 (t, $J_{C\text{-}F}$ = 15.7 Hz, 1C) 74.6, 66.8 (2C), 64.1, 55.0, 53.6 (2C)

FTIR (thin film): cm^{-1} 2813, 1737, 1652, 1524, 1495, 1325, 1225, 1117, 997, 871, 733

HRMS-ESI (m/z) Calcd for ($C_{20}H_{19}F_5NO_4$) ($[M+H]^+$): 432.1229; found: 432.1228

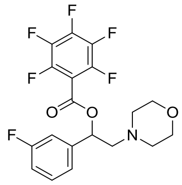

6c

1-(3-Fluorophenyl)-2-morpholinoethyl 2,3,4,5,6-pentafluorobenzoate (6c).

Prepared using standard conditions. Purification by flash column chromatography (100% hexanes → 20% ethyl acetate–hexanes) gave **6c** as a white solid (95.4 mg, 57%).

R_f = 0.59 (50% ethyl acetate–hexanes)

^{1}H NMR ($CDCl_3$, 400 MHz): δ 7.39– 7.30 (m, 1H), 7.17 (d, *J* = 7.2 Hz, 1H), 7.12 (d, *J* = 7.1 Hz, 1H), 7.07–6.99 (m, 1H), 6.22 (dd, *J* = 9.5, 3.7 Hz, 1H), 3.72–3.59 (m, 4H), 2.85 (dd, *J* = 13.6, 9.5 Hz, 1H), 2.71–2.60 (m, 3H), 2.52–2.42 (m, 2H)

^{13}C NMR ($CDCl_3$, 125 MHz): δ 162.7 (d, $J_{C\text{-}F}$ = 246.4 Hz, 1C), 158.0, 145.4 (d, $J_{C\text{-}F}$ = 263.5 Hz, 2C), 143.2 (d, $J_{C\text{-}F}$ = 259.3 Hz, 1C), 140.0 (d, $J_{C\text{-}F}$ = 7.1 Hz, 1C), 137.6 (d, $J_{C\text{-}F}$ = 255.0 Hz, 2C), 130.1 (d, $J_{C\text{-}F}$ = 8.1 Hz, 1C), 122.1, 115.3 (d, $J_{C\text{-}F}$ = 21.1 Hz, 1C), 113.4 (d, $J_{C\text{-}F}$ = 22.4 Hz, 1C), 108.3 (t, $J_{C\text{-}F}$ = 15.5 Hz, 1C), 74.1, 66.9 (2C), 64.0, 53.7 (2C)

FTIR (thin film): cm^{-1} 2814, 1737, 1651, 1592, 1523, 14941325, 1220, 1116, 996, 733

HRMS-ESI (m/z) Calcd for ($C_{19}H_{16}F_6NO_3$) ($[M+H]^+$): 420.1029; found: 420.1029

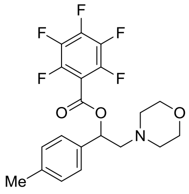

6d

2-Morpholino-1-(p-tolyl)ethyl 2,3,4,5,6-pentafluorobenzoate (6d).

Prepared using standard conditions. Purification by flash column chromatography (100% hexanes → 20% ethyl acetate–hexanes) gave **6d** as a white solid (134.5 mg, 81%).

R_f = 0.65 (50% ethyl acetate–hexanes)

^{1}H NMR ($CDCl_3$, 400 MHz): δ 7.30 (d, J = 8.1 Hz, 2H), 7.19 (d, J = 8.1 Hz, 2H), 6.24 (dd, J = 9.8, 3.4 Hz, 1H), 3.73–3.62 (m, 4H), 2.90 (dd, J = 13.6, 9.8 Hz, 1H), 2.73–2.60 (m, 3H), 2.52–2.41 (m, 2H), 2.36 (s, 3H)

^{13}C NMR ($CDCl_3$, 125 MHz): δ 158.0, 145.2 (d, J_{C-F} = 253.3 Hz, 2C), 143.0 (d, J_{C-F} = 260.0 Hz, 1C), 138.2, 137.6 (d, J_{C-F} = 251.4 Hz, 2C), 134.5, 129.1 (2C), 126.4 (2C), 108.4 (t, J_{C-F} = 15.7 Hz, 1C), 74.7, 66.8 (2C), 64.0, 53.6 (2C), 20.9

FTIR (thin film): cm^{-1} 2811, 1736, 1652, 1523, 1494, 1326, 1223, 1116, 993, 946, 541

HRMS-ESI (m/z) Calcd for ($C_{20}H_{19}F_5NO_3$) ($[M+H]^+$): 416.1280; found: 416.1280

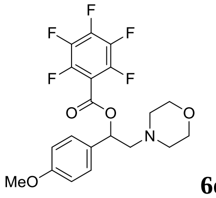

6e

1-(4-Methoxyphenyl)-2-morpholinoethyl 2,3,4,5,6-pentafluorobenzoate (6e).

Prepared using standard conditions. Purification by flash column chromatography (100% hexanes → 30% ethyl acetate–hexanes) gave **6e** as a beige powder (119.2 mg, 69%).

R_f = 0.45 (50% ethyl acetate–hexanes)

^{1}H NMR ($CDCl_3$, 400 MHz): δ 7.33 (d, J = 8.6 Hz, 2H), 6.90 (d, J = 8.6 Hz, 2H), 6.21 (dd, J = 9.7, 3.5 Hz, 1H), 3.80 (s, 3H), 3.71–3.61 (m, 4H), 2.90 (dd, J = 13.5, 9.7 Hz, 1H), 2.71–2.58 (m, 3H), 2.51–2.41 (m, 2H)

^{13}C NMR ($CDCl_3$, 125 MHz): δ 159.6, 157.9, 145.2 (d, J_{C-F} = 258.1 Hz, 2C), 142.8 (d, J_{C-F} = 258.3 Hz, 1C), 137.5 (d, J_{C-F} = 253.8 Hz, 2C), 129.4, 127.9 (2C), 113.7 (2C), 108.4 (t, J_{C-F} = 15.4 Hz, 1C), 74.5, 66.8 (2C), 63.8, 54.9, 53.6 (2C)

FTIR (thin film): cm^{-1} 2811, 1734, 1652, 1614, 1495, 1327, 1223, 1177, 1116, 993, 946, 831

HRMS-ESI (m/z) Calcd for ($C_{20}H_{19}F_5NO_4$) ($[M+H]^+$): 432.1229; found: 432.1229

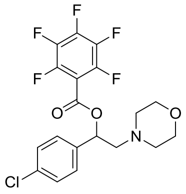

6f

1-(4-Chlorophenyl)-2-morpholinoethyl 2,3,4,5,6-pentafluorobenzoate (6f).

Prepared using standard conditions. Purification by flash column chromatography (100% hexanes → 25% ethyl acetate–hexanes) gave **6f** as a white solid (121.8 mg, 70%).

R_f = 0.69 (50% ethyl acetate–hexanes)

^{1}H NMR ($CDCl_3$, 400 MHz): δ 7.33 (s, 4H), 6.18 (dd, *J* = 9.4, 3.8 Hz, 1H), 3.68–3.59 (m, 4H), 2.84 (dd, 13.6, 9.4 Hz, 1H), 2.68–2.58 (m, 3H), 2.49–2.41 (m, 2H)

^{13}C NMR ($CDCl_3$, 125 MHz): δ 157.9, 145.3 (d, $J_{C\text{-}F}$ = 263.7 Hz, 2C), 143.1 (d, $J_{C\text{-}F}$ = 255.6 Hz, 1C), 137.6 (d, $J_{C\text{-}F}$ = 252.4 Hz, 2C), 136.0, 134.2, 128.7 (2C), 127.9 (2C), 108.1 (t, $J_{C\text{-}F}$ = 15.4 Hz, 1C), 74.1, 66.8 (2C), 63.8, 53.6 (2C)

FTIR (thin film): cm^{-1} 2811, 1737, 1652, 1523, 1494, 1325, 1223, 1117, 1009

HRMS-ESI (m/z) Calcd for ($C_{19}H_{16}ClF_5NO_3$) ($[M+H]^+$): 436.0733; found: 436.0734

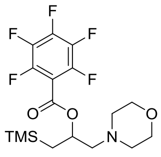

6g

1-Morpholino-3-(trimethylsilyl)propan-2-yl 2,3,4,5,6-pentafluorobenzoate (6g).

Prepared using standard conditions. Purification by flash column chromatography (100% hexanes → 15% ethyl acetate–hexanes) gave **6g** as a clear oil (31.7 mg, 19%).

R_f = 0.78 (50% ethyl acetate–hexanes)

^{1}H NMR ($CDCl_3$, 400 MHz): δ 5.51–5.43 (m, 1H), 3.63 (t, *J* = 4.5 Hz, 4H), 2.67–2.59 (m, 2H), 2.54 (dd, *J* = 13.3, 9.0 Hz, 1H), 2.44–2.33 (m, 3H), 1.10–0.96 (m, 2H), 0.06 (s, 9H)

^{13}C NMR ($CDCl_3$, 125 MHz): δ 158.5, 145.1 (d, $J_{C\text{-}F}$ = 258.5 Hz, 2C), 142.9 (d, $J_{C\text{-}F}$ = 281.9 Hz, 1C), 137.6 (d, $J_{C\text{-}F}$ = 248.0 Hz, 2C), 109.0 (t, $J_{C\text{-}F}$ = 15.0 Hz, 1C), 72.4, 66.9 (2C), 64.7, 53.8 (2C), 21.0, –1.0 (3C)

FTIR (thin film): cm^{-1} 2957, 1735, 1652, 1523, 1497, 1323, 1233, 1118, 1004, 859, 840

HRMS-ESI (m/z) Calcd for ($C_{17}H_{23}F_5NO_3Si$) ($[M+H]^+$): 412.1362; found: 412.1360

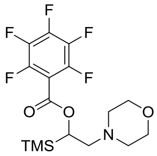

6h

2-Morpholino-1-(trimethylsilyl)ethyl 2,3,4,5,6-pentafluorobenzoate (6h).

Prepared using standard conditions. Purification by flash column chromatography (100% hexanes → 15% ethyl acetate–hexanes) gave **6h** as a clear oil (14.2 mg, 9%).

R_f = 0.38 (50% ethyl acetate–hexanes)

^{1}H NMR ($CDCl_3$, 400 MHz): δ 5.28 (dd, *J* = 10.8, 3.2 Hz, 1H), 3.70–3.60 (m, 4H), 2.73 (dd, 13.6, 10.8 Hz, 1H), 2.68–2.60 (m, 2H), 2.46 (dd, *J* = 13.6, 3.2 Hz, 1H), 2.36–2.27 (m, 2H), 0.12 (s, 9H)

^{13}C NMR ($CDCl_3$, 125 MHz): δ (note: sample mass was too small to accurately determine C_{aryl}-F peak/coupling) 159.1, 68.3, 67.0 (2C), 59.2, 53.5 (2C), –3.6 (3C)

FTIR (thin film): cm^{-1} 2958, 1734, 1652, 1523, 1499, 1336, 1229, 1118, 1004, 867, 844

HRMS-ESI (m/z) Calcd for ($C_{16}H_{21}F_5NO_3Si$) ($[M+H]^+$): 398.1205; found: 398.1205

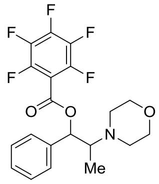

6i

2-Morpholino-1-phenylpropyl 2,3,4,5,6-pentafluorobenzoate (6i).

Prepared using standard conditions. Purification by flash column chromatography (100% hexanes → 30% ethyl acetate–hexanes) gave two diastereomers of **6i** (total, 114.9 mg, 69%).

First diastereomer

A clear oil (71.9 mg, 43%)

R_f = 0.74 (50% ethyl acetate–hexanes)

^{1}H NMR ($CDCl_3$, 400 MHz): δ 7.42–7.33 (m, 5H), 5.99 (d, *J* = 9.6 Hz, 1H), 3.70–3.57 (m, 4H), 3.03–2.94 (m, 1H), 2.81–2.73 (m, 2H), 2.54–2.45 (m, 2H), 0.78 (d, *J* = 6.9 Hz, 3H)

^{13}C NMR ($CDCl_3$, 125 MHz): δ 157.8, 145.5 (d, $J_{C\text{-}F}$ = 257.9 Hz, 2C), 143.0 (d, $J_{C\text{-}F}$ = 259.7 Hz, 1C), 137.6 (d, $J_{C\text{-}F}$ = 254.4 Hz, 2C), 137.0, 128.6, 128.5 (2C), 127.5 (2C), 108.3 (t, $J_{C\text{-}F}$ = 15.9 Hz, 1C), 78.4, 67.2 (2C), 64.0, 49.0 (2C), 9.2

FTIR (thin film): cm^{-1} 2963, 1736, 1652, 1524, 1497, 1338, 1233, 1118, 1004, 701

HRMS-ESI (m/z) Calcd for ($C_{20}H_{19}F_5NO_3$) ($[M+H]^+$): 416.1280; found: 416.1279

Second diastereomer

A clear oil (43.0 mg, 26%)

R_f = 0.51 (50% ethyl acetate–hexanes)

^{1}H NMR ($CDCl_3$, 400 MHz): δ 7.40–7.27 (m, 5H), 6.20 (d, *J* = 5.1 Hz, 1H), 3.70–3.55 (m, 4H), 2.98–2.90 (m, 1H), 2.67–2.54 (m, 4H), 1.13 (d, *J* = 6.8 Hz, 3H)

^{13}C NMR ($CDCl_3$, 125 MHz): δ 158.0, 145.4 (d, $J_{C\text{-}F}$ = 252.8 Hz, 2C), 143.3 (d, $J_{C\text{-}F}$ = 263.5 Hz, 1C), 138.2, 137.7 (d, $J_{C\text{-}F}$ = 258.5 Hz, 2C), 128.3 (2C), 128.0, 126.3 (2C), 108.0 (t, $J_{C\text{-}F}$ = 14.8 Hz, 1C), 78.2, 67.0 (2C), 64.2, 49.8 (2C), 9.6

FTIR (thin film): cm^{-1} 2965, 1739, 1652, 1524, 1497, 1327, 1228, 1119, 1004, 952, 701

HRMS-ESI (m/z) Calcd for ($C_{20}H_{19}F_5NO_3$) ($[M+H]^+$): 416.1280; found: 416.1281

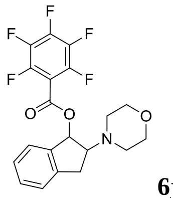

6j

2-Morpholino-2,3-dihydro-1H-inden-1-yl 2,3,4,5,6-pentafluorobenzoate (6j).

Prepared using standard conditions. Purification by flash column chromatography (100% hexanes → 20% ethyl acetate–hexanes) gave two diastereomers of **6j** (total, 146.7 mg, 89%)..

Minor diastereomer

A white solid (18.4 mg, 11%)

R_f = 0.37 (50% ethyl acetate–hexanes)

^{1}H NMR ($CDCl_3$, 400 MHz): δ 7.54 (d, J = 7.4 Hz, 1H), 7.39–7.33 (m, 1H), 7.33–7.23 (m, 2H), 6.42 (d, J = 4.5 Hz, 1H), 3.75 (t, J = 4.6 Hz, 4H), 3.26–3.16 (m, 2H), 3.06 (dd, J = 18.3, 10.5 Hz, 1H), 2.78–2.62 (m, 4H)

^{13}C NMR ($CDCl_3$, 125 MHz): δ (note: sample mass was too small to accurately determine C_{aryl}-F peaks/coupling) 158.8, 142.4, 138.3, 130.1, 127.3, 126.3, 124.9, 78.0, 68.8, 66.7 (2C), 52.2 (2C), 33.6

FTIR (thin film): cm^{-1} 2959, 1735, 1653, 1523, 1501, 1348, 1324, 1229, 1118, 1000, 760

HRMS-ESI (m/z) Calcd for ($C_{20}H_{17}F_5NO_3$) ($[M+H]^+$): 414.1123; found: 414.1122

Major diastereomer

A white solid (128.3 mg, 78%)

R_f = 0.41 (50% ethyl acetate–hexanes)

^{1}H NMR ($CDCl_3$, 400 MHz): δ 7.34–7.27 (m, 2H), 7.27–7.20 (m, 2H), 6.67 (d, J = 5.6 Hz, 1H), 3.72 (t, J = 4.5 Hz, 4H), 3.47 (ddd, J = 8.1, 7.4, 5.6 Hz, 1H), 3.24 (dd, J = 15.9, 8.1 Hz, 1H), 2.94 (dd, J = 15.9, 7.4 Hz, 1H), 2.60 (t, J = 4.5 Hz, 4H)

^{13}C NMR ($CDCl_3$, 125 MHz): δ 158.8, 145.0 (d, J_{C-F} = 252.5 Hz, 2C), 143.0 (d, J_{C-F} = 253.8 Hz, 1C), 140.7, 138.4, 137.6 (d, J_{C-F} = 255.3 Hz, 2C), 129.3, 127.3, 124.7 (2C), 108.3 (t, J_{C-F} = 17.0 Hz, 1C), 81.6, 71.6, 66.8 (2C), 51.1 (2C), 33.5

FTIR (thin film): cm^{-1} 2955, 1734, 1653, 1523, 1497, 1346, 1322, 1228, 1118, 1002, 759

HRMS-ESI (m/z) Calcd for ($C_{20}H_{17}F_5NO_3$) ($[M+H]^+$): 414.1123; found: 414.1122

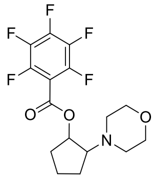

6k

2-Morpholinocyclopentyl 2,3,4,5,6-pentafluorobenzoate (6k).

Prepared using standard conditions. Purification by flash column chromatography (100% hexanes → 65% ethyl acetate–hexanes) gave **6k** as a clear oil (51.7 mg, 35%).

R_f = 0.19 (50% ethyl acetate–hexanes)

^{1}H NMR ($CDCl_3$, 400 MHz): δ 5.42–5.36 (m, 1H), 3.70 (t, J = 4.5 Hz, 4H), 2.83 (td, J = 8.0, 4.2 Hz, 1H), 2.58–2.47 (m, 4H), 2.20–2.08 (m, 1H), 2.05–1.95 (m, 1H), 1.83 (m, 3H), 1.60–1.48 (m, 1H)

^{13}C NMR ($CDCl_3$, 125 MHz): δ 158.2, 145.1 (d, J_{C-F} = 261.4 Hz, 2C), 143.0 (d, J_{C-F} = 270.3 Hz, 1C), 137.5 (d, J_{C-F} = 255.2 Hz, 2C), 108.4 (t, J_{C-F} = 15.0 Hz, 1C), 80.7, 71.9, 66.7 (2C), 51.9 (2C), 32.4, 28.8, 22.4

FTIR (thin film): cm^{-1} 2963, 1734, 1652, 1523, 1497, 1329, 1231, 1119, 998, 761

HRMS-ESI (m/z) Calcd for ($C_{16}H_{17}F_5NO_3$) ($[M+H]^+$): 366.1123; found: 366.1123

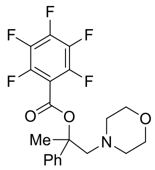

6l

1-Morpholino-2-phenylpropan-2-yl 2,3,4,5,6-pentafluorobenzoate (6l).

Prepared using standard conditions. Purification by flash column chromatography (100% hexanes → 50% ethyl acetate–hexanes) gave **6l** as a clear oil (containing impurities, 22.2 mg, <15%).

R_f = 0.20 (25% ethyl acetate–hexanes)

^{1}H NMR ($CDCl_3$, 400 MHz): δ 7.45–7.21 (m, 5H), 3.56 (t, *J* = 4.7 Hz, 4H), 2.87 (d, *J* = 14.1 Hz, 1H), 2.80 (d, *J* = 14.1 Hz, 1H), 2.47–2.39 (m, 2H), 2.32–2.25 (m, 2H), 2.06 (s, 3H)

IV. References

(1) Berman, A. M.; Johnson, J. S. *J. Org. Chem.* **2006**, *71*, 219–224.
(2) Samaddar, A. K.; Konar, S. K.; Nasipuri, D. *J. Chem. Soc., Perkin Trans. 1* **1983**, 1449–1451.
(3) Watson, A. J. A.; Maxwell, A. C.; Williams, J. M. J. *J. Org. Chem.* **2011**, *76*, 2328–2331.

V. Spectra

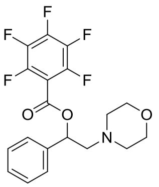
F
F
F
F
F
O
O
N
O

4a

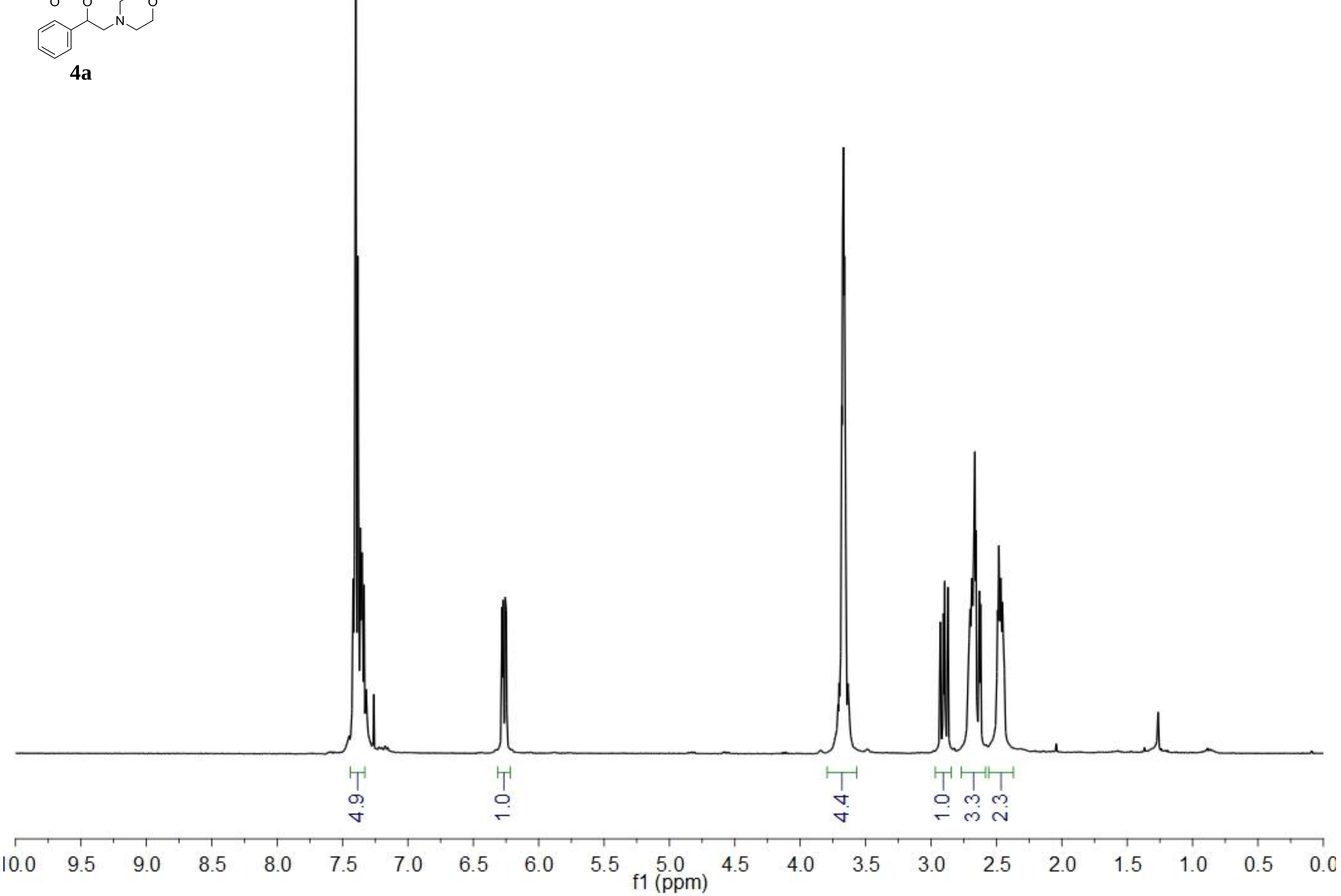
4.9
1.0
4.4
1.0
3.3
2.3
10.0 9.5 9.0 8.5 8.0 7.5 7.0 6.5 6.0 5.5 5.0 4.5 4.0 3.5 3.0 2.5 2.0 1.5 1.0 0.5 0.0
f1 (ppm)

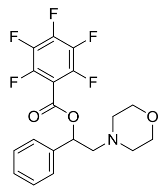
F
F
F
F
F
O
O
N
O

4a

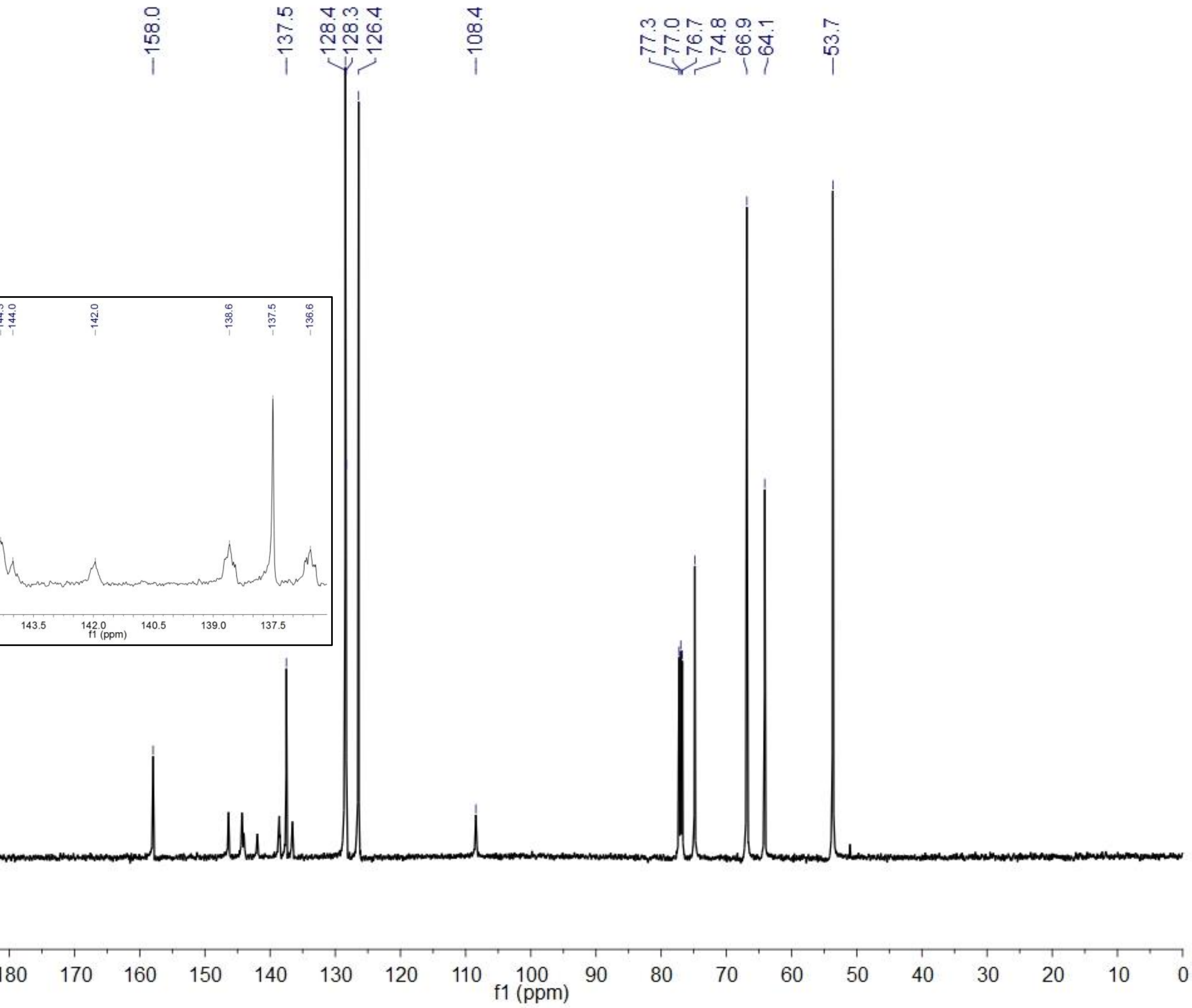
158.0
137.5
128.4
128.3
126.4
108.4
77.3
77.0
76.7
74.8
66.9
64.1
53.7
146.4
144.3
144.0
142.0
138.6
137.5
136.6
146.5 145.0 143.5 142.0 140.5 139.0 137.5
f1 (ppm)
200 190 180 170 160 150 140 130 120 110 100 90 80 70 60 50 40 30 20 10 0
f1 (ppm)

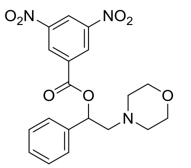
O2N
NO2
O
O
N
O

4b

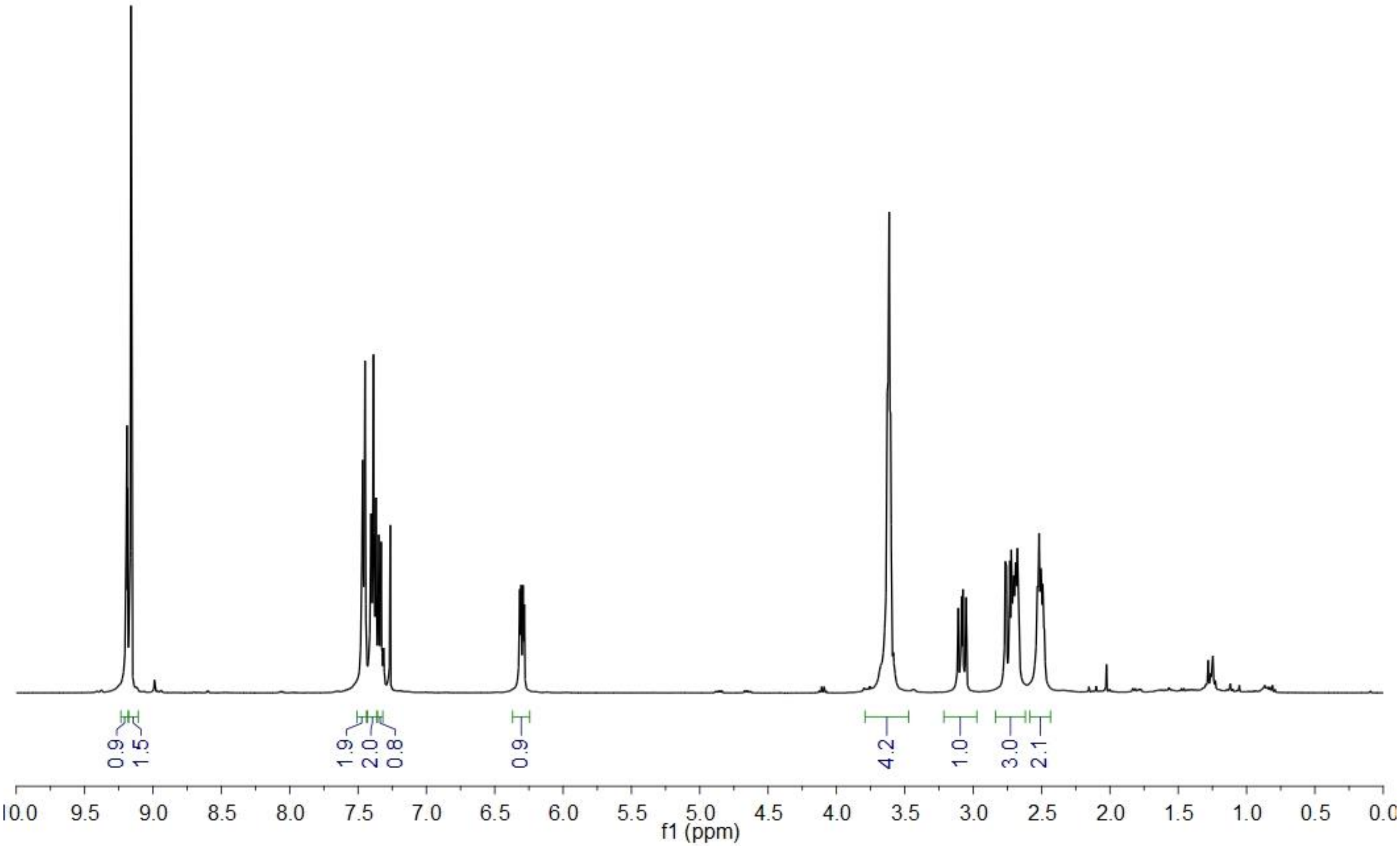
0.9
1.5
1.9
2.0
0.8
0.9
4.2
1.0
3.0
2.1
10.0 9.5 9.0 8.5 8.0 7.5 7.0 6.5 6.0 5.5 5.0 4.5 4.0 3.5 3.0 2.5 2.0 1.5 1.0 0.5 0.0
f1 (ppm)

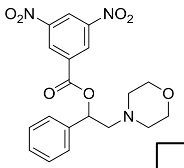

O_2N
NO_2
O
O
N
O

4b

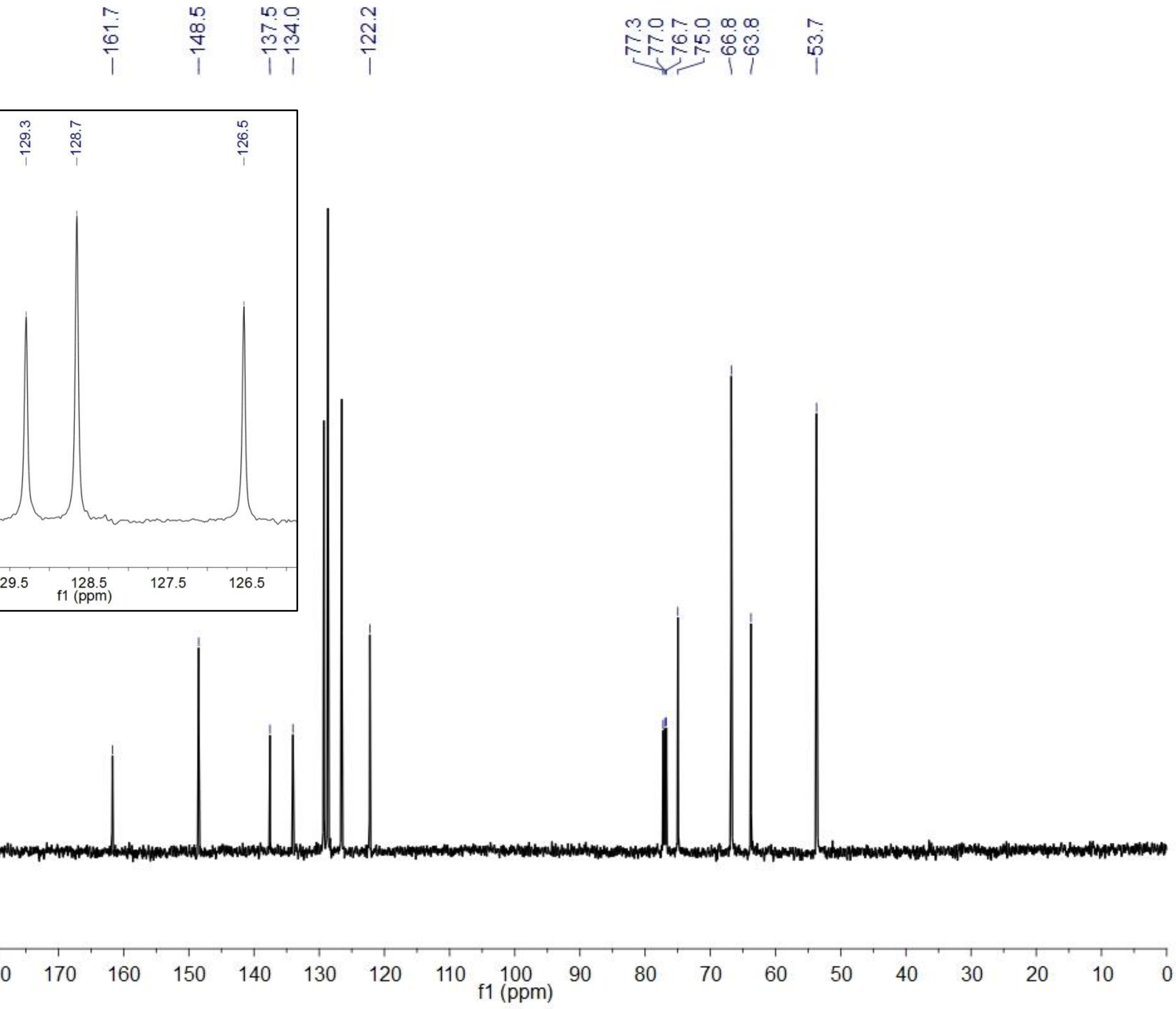

161.7
148.5
137.5
134.0
122.2
77.3
77.0
76.7
75.0
66.8
63.8
53.7
129.3
128.7
126.5
129.5
128.5
127.5
126.5
f1 (ppm)
200
190
180
170
160
150
140
130
120
110
100
90
80
70
60
50
40
30
20
10
0
f1 (ppm)

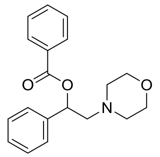

4c

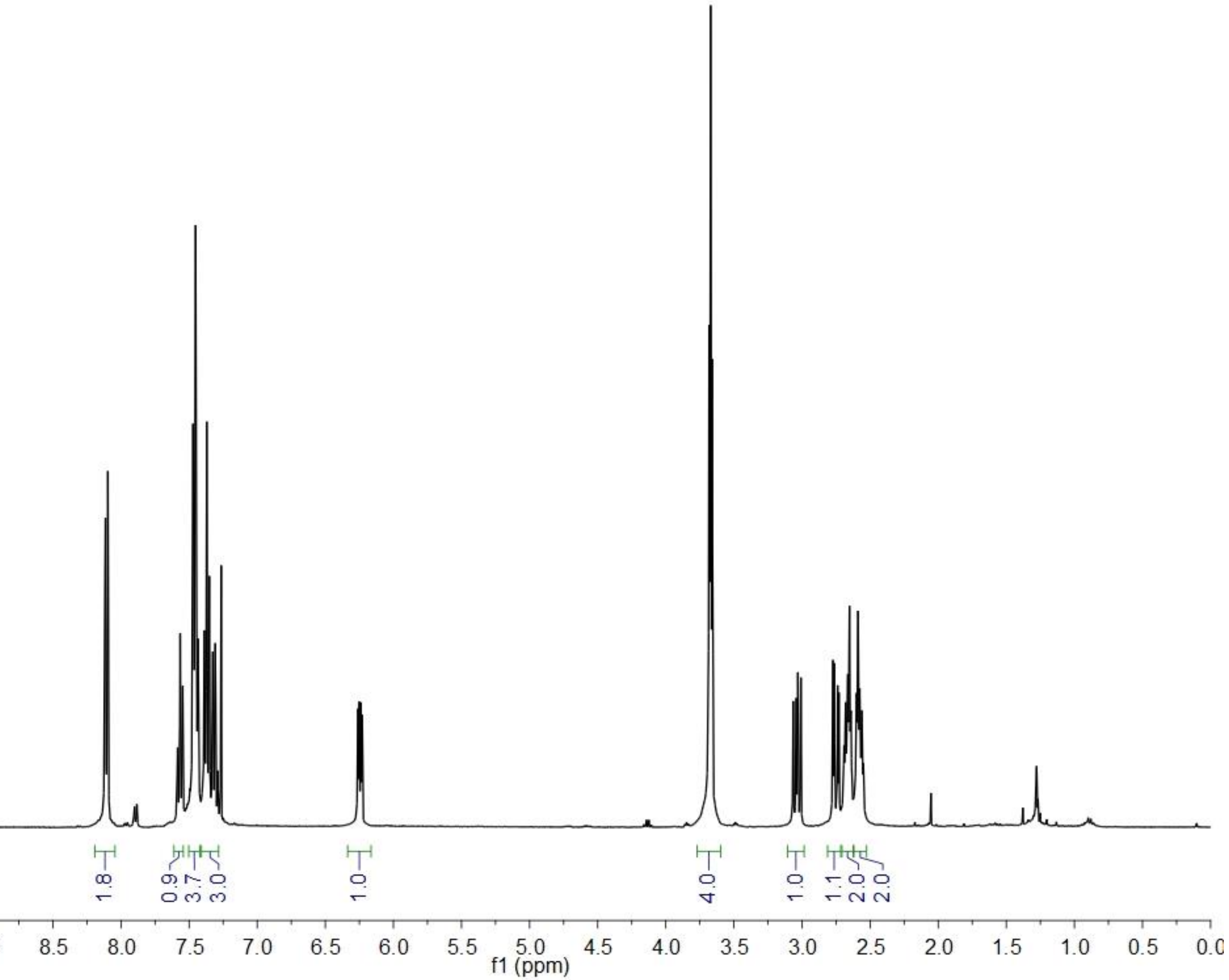
1.8
0.9
3.7
3.0
1.0
4.0
1.0
1.1
2.0
2.0
10.0 9.5 9.0 8.5 8.0 7.5 7.0 6.5 6.0 5.5 5.0 4.5 4.0 3.5 3.0 2.5 2.0 1.5 1.0 0.5 0.0
f1 (ppm)

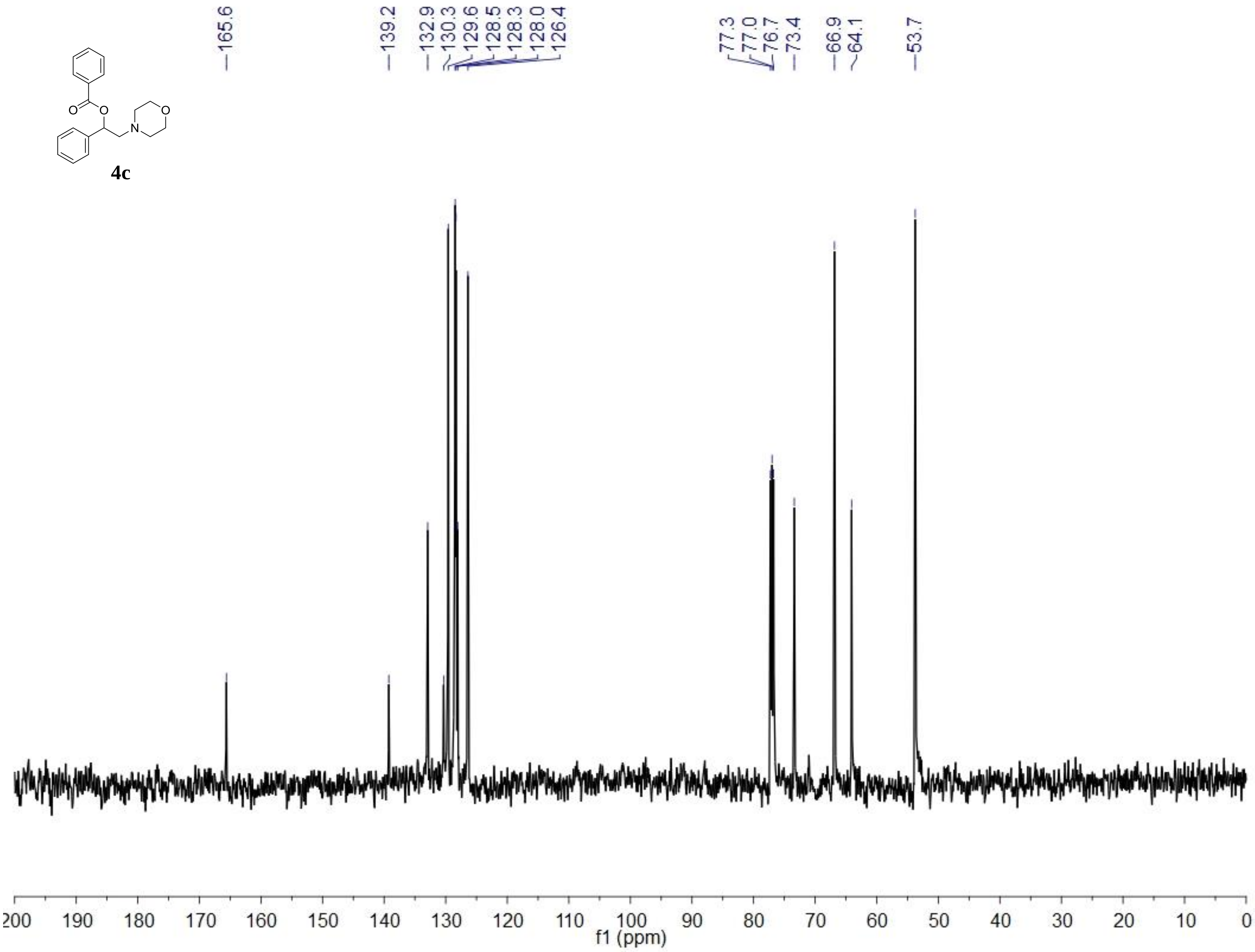
4c
165.6
139.2
132.9
130.3
129.6
128.5
128.3
128.0
126.4
77.3
77.0
76.7
73.4
66.9
64.1
53.7
200 190 180 170 160 150 140 130 120 110 100 90 80 70 60 50 40 30 20 10 0
f1 (ppm)

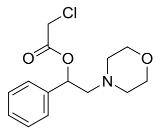
Cl
O
O
N
O

4d

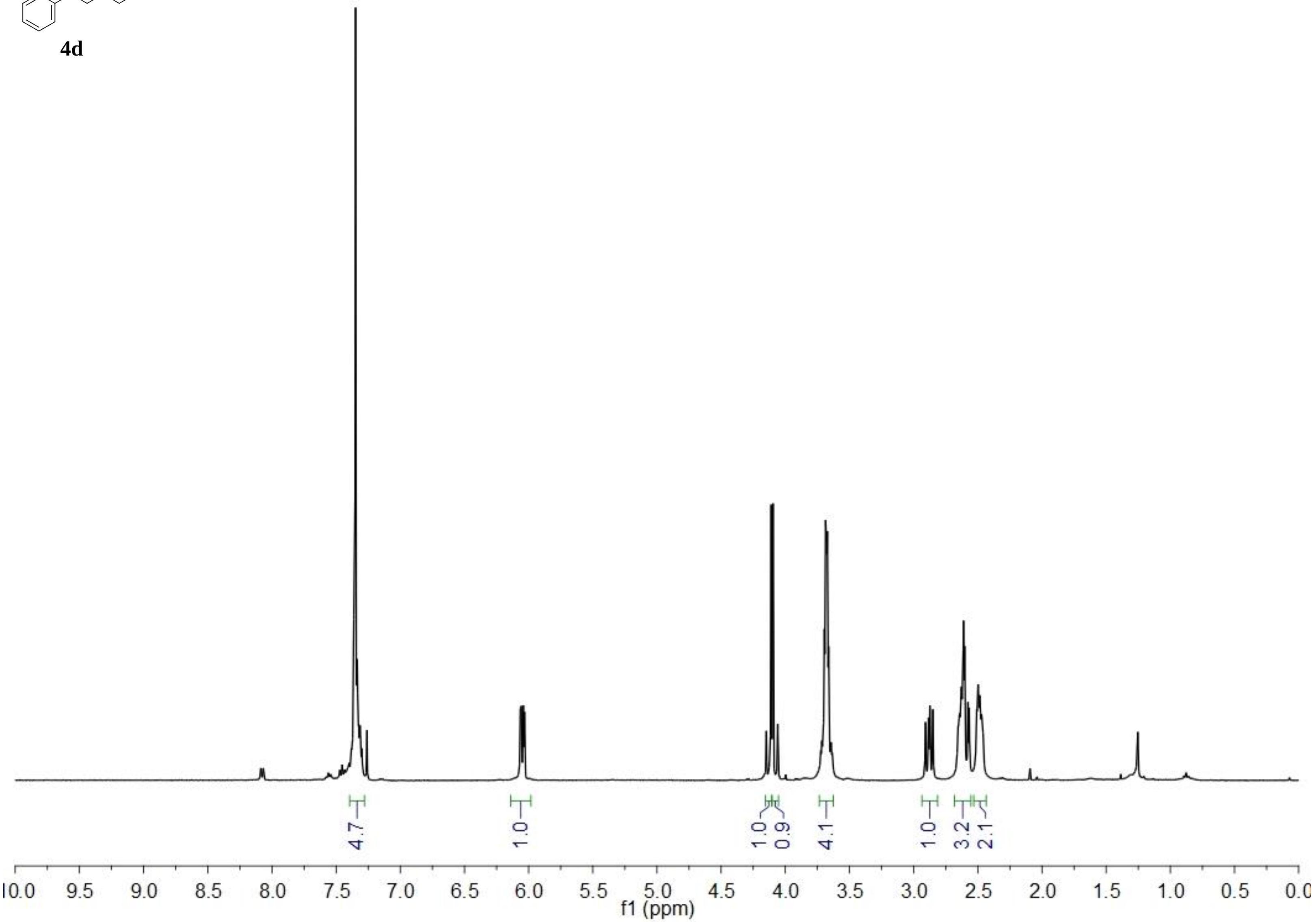
4.7
1.0
1.0
0.9
4.1
1.0
3.2
2.1
10.0
9.5
9.0
8.5
8.0
7.5
7.0
6.5
6.0
5.5
5.0
4.5
4.0
3.5
3.0
2.5
2.0
1.5
1.0
0.5
0.0
f1 (ppm)

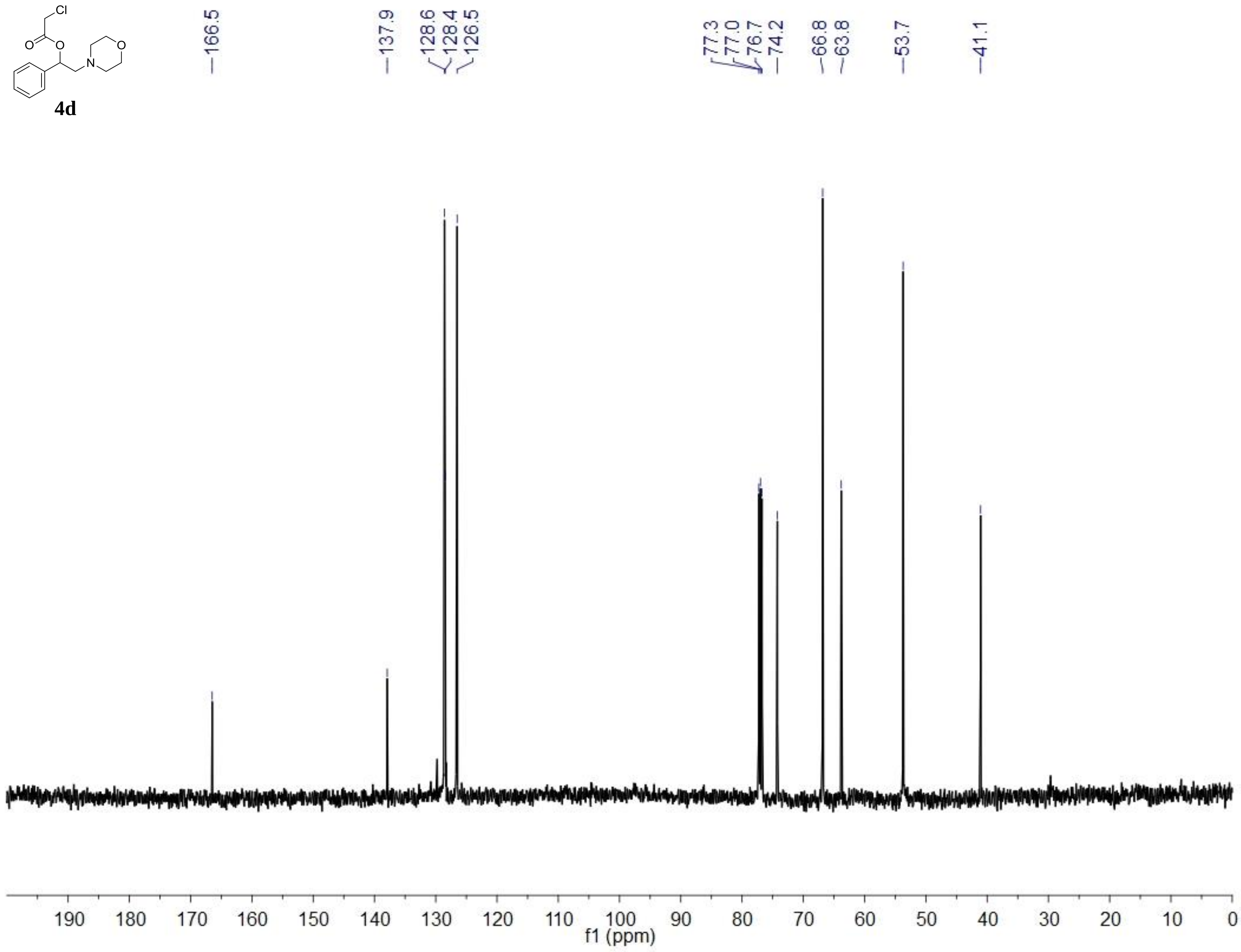
Cl
O
O
N
O
4d
166.5
137.9
128.6
128.4
126.5
77.3
77.0
76.7
74.2
66.8
63.8
53.7
41.1
190 180 170 160 150 140 130 120 110 100 90 80 70 60 50 40 30 20 10 0
f1 (ppm)

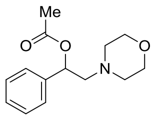
Me
O
O
N
O

4e

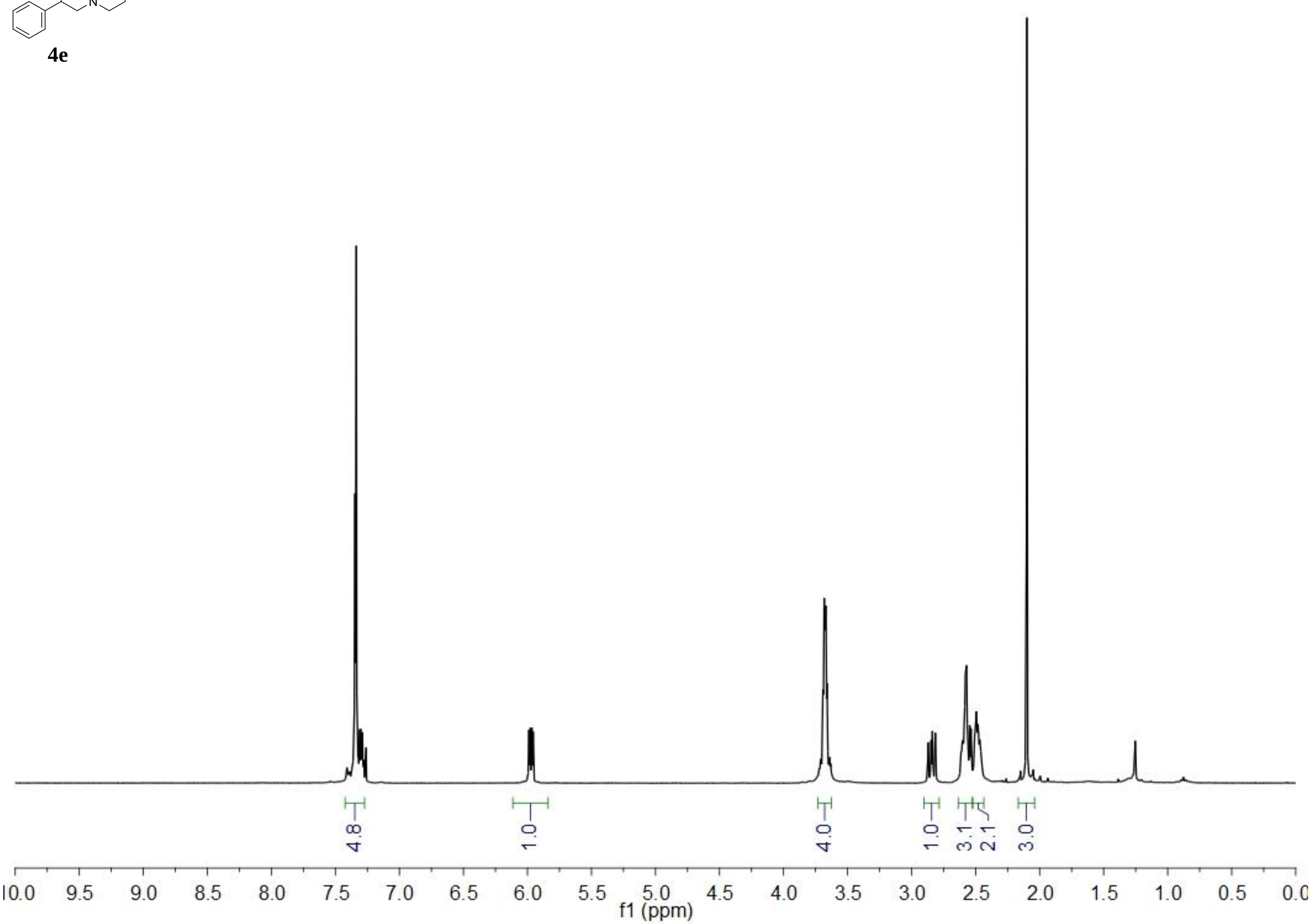
4.8
1.0
4.0
1.0
3.1
2.1
3.0
10.0
9.5
9.0
8.5
8.0
7.5
7.0
6.5
6.0
5.5
5.0
4.5
4.0
3.5
3.0
2.5
2.0
1.5
1.0
0.5
0.0
f1 (ppm)

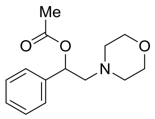
Me
O
O
N
O

4e

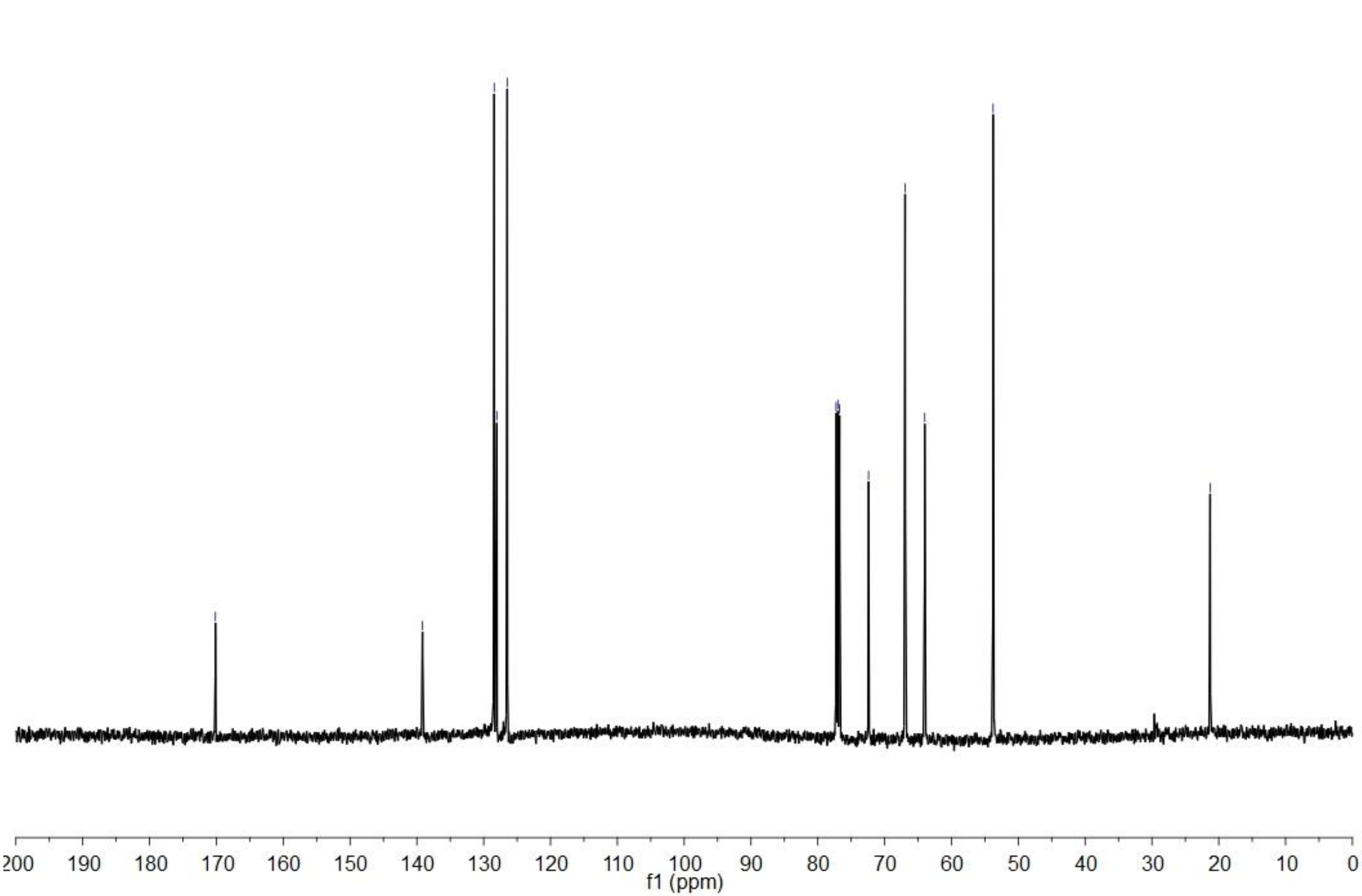
170.1
139.2
128.4
128.0
126.5
77.3
77.0
76.7
72.4
66.9
64.0
53.8
21.3
200 190 180 170 160 150 140 130 120 110 100 90 80 70 60 50 40 30 20 10 0
f1 (ppm)

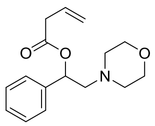
O
O
N
O

4f

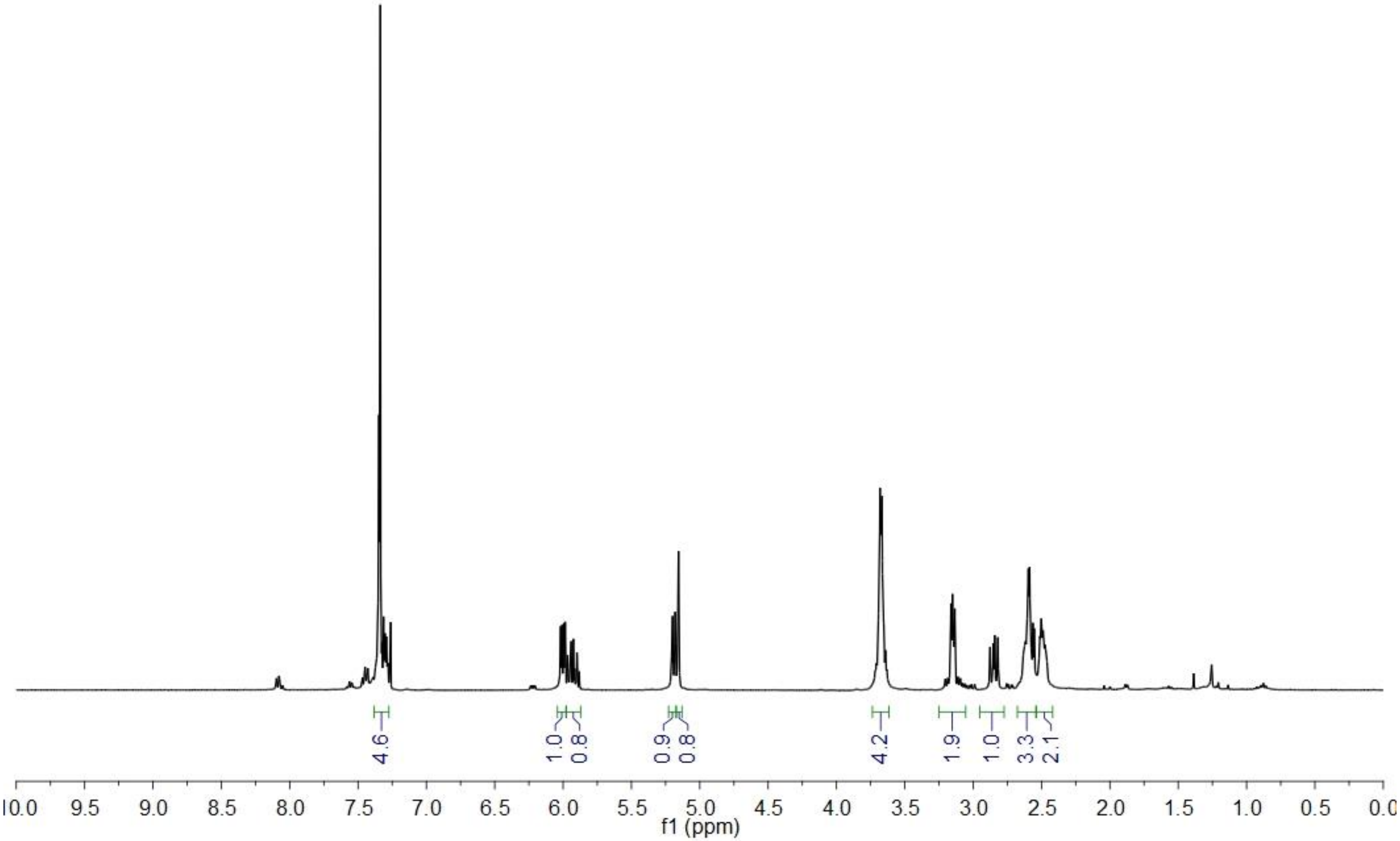
4.6
1.0
0.8
0.9
0.8
4.2
1.9
1.0
3.3
2.1
10.0 9.5 9.0 8.5 8.0 7.5 7.0 6.5 6.0 5.5 5.0 4.5 4.0 3.5 3.0 2.5 2.0 1.5 1.0 0.5 0.0
f1 (ppm)

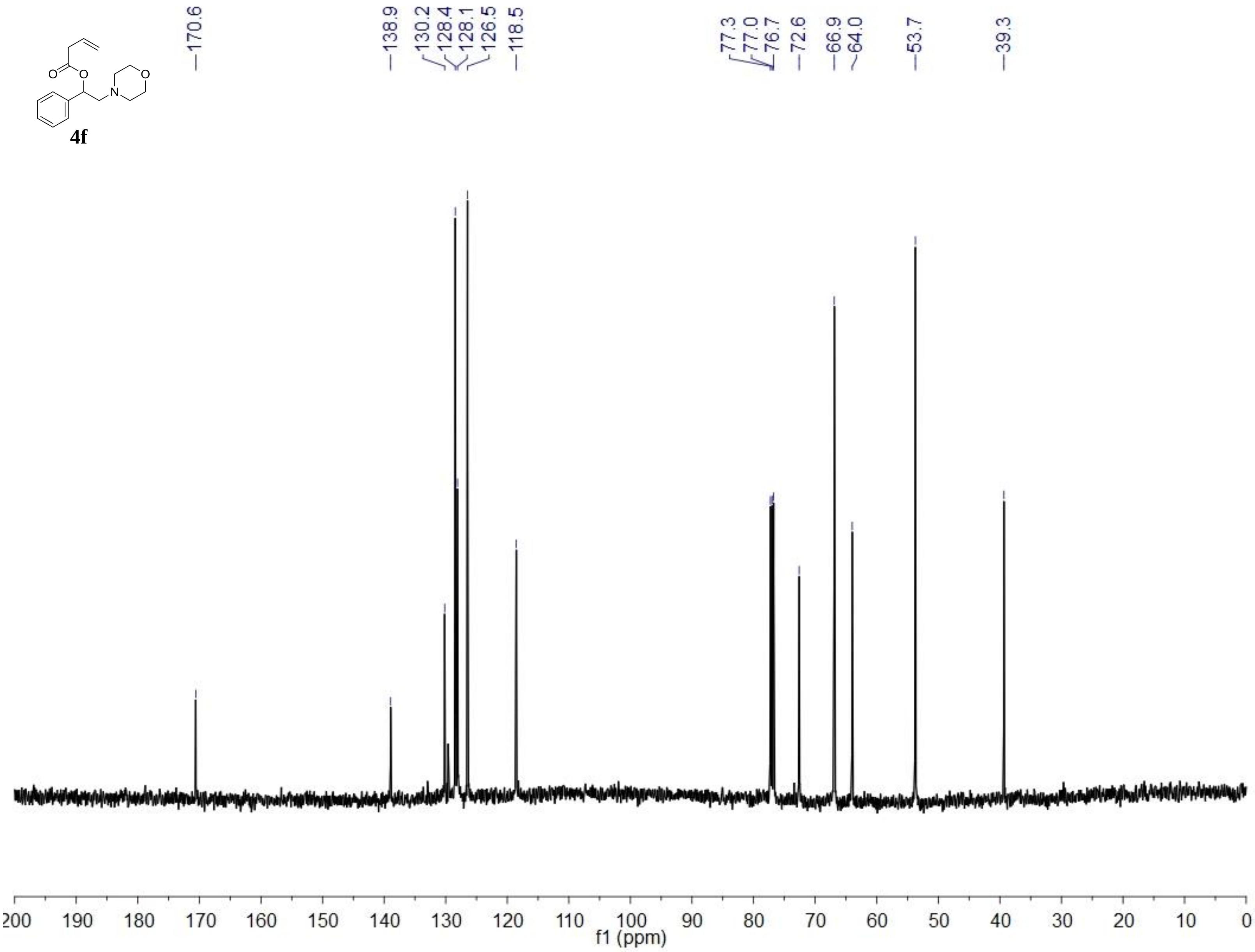
4f
170.6
138.9
130.2
128.4
128.1
126.5
118.5
77.3
77.0
76.7
72.6
66.9
64.0
53.7
39.3
200 190 180 170 160 150 140 130 120 110 100 90 80 70 60 50 40 30 20 10 0
f1 (ppm)

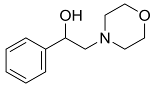
OH
N
O

4g

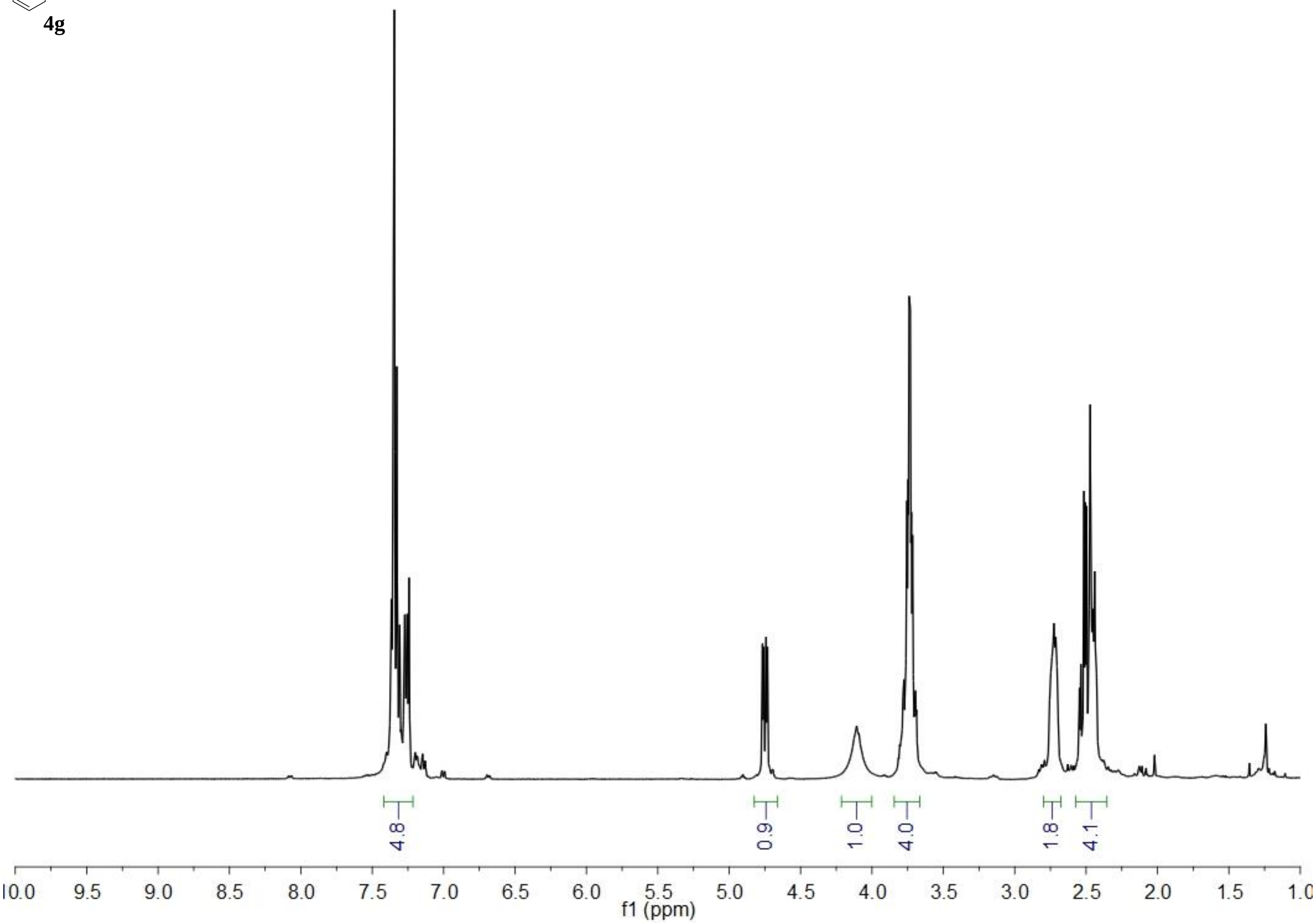
4.8
0.9
1.0
4.0
1.8
4.1
10.0 9.5 9.0 8.5 8.0 7.5 7.0 6.5 6.0 5.5 5.0 4.5 4.0 3.5 3.0 2.5 2.0 1.5 1.0
f1 (ppm)

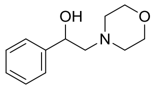
OH
N
O

4g

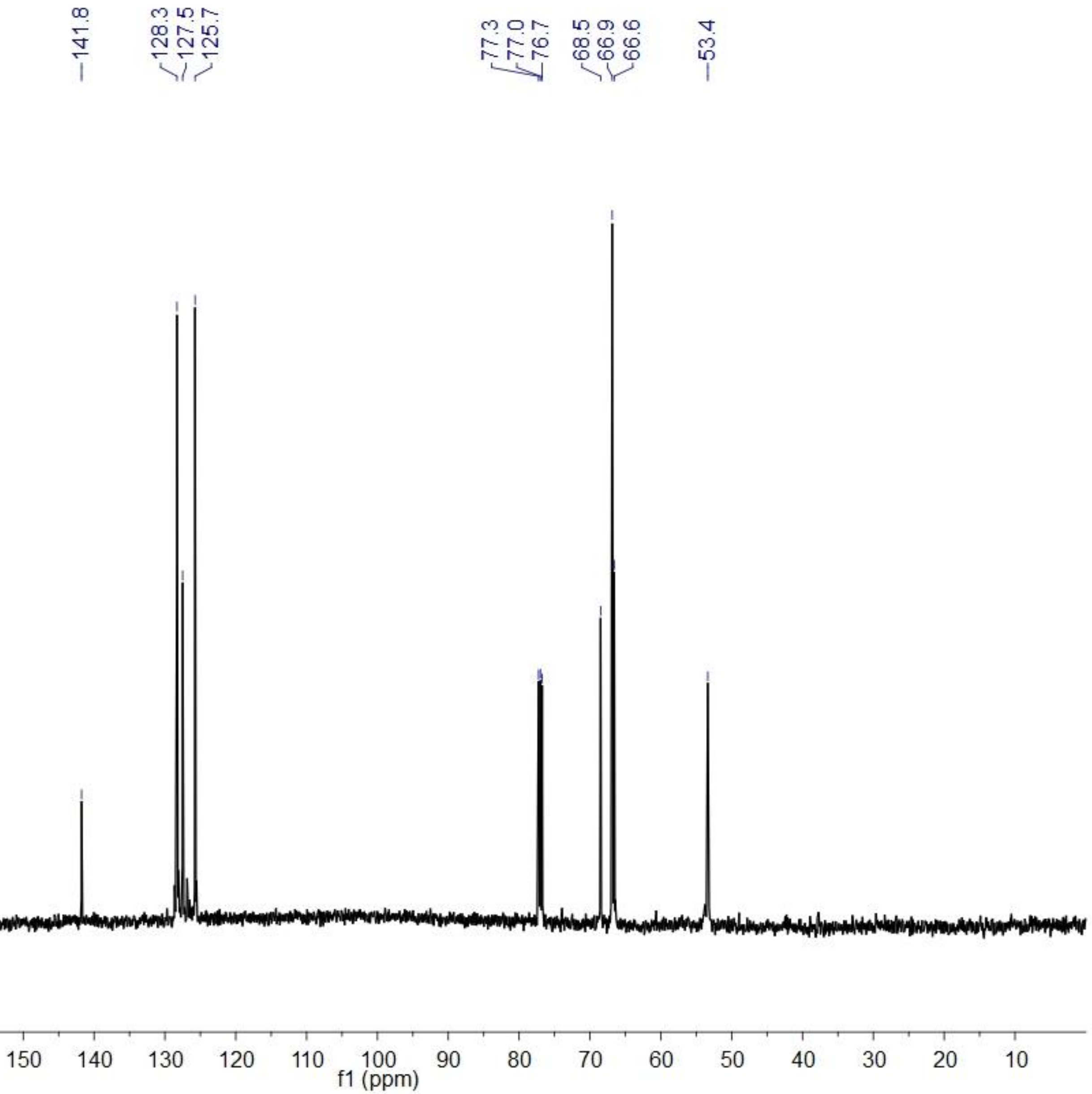
141.8
128.3
127.5
125.7
77.3
77.0
76.7
68.5
66.9
66.6
53.4
200
190
180
170
160
150
140
130
120
110
100
90
80
70
60
50
40
30
20
10
f1 (ppm)

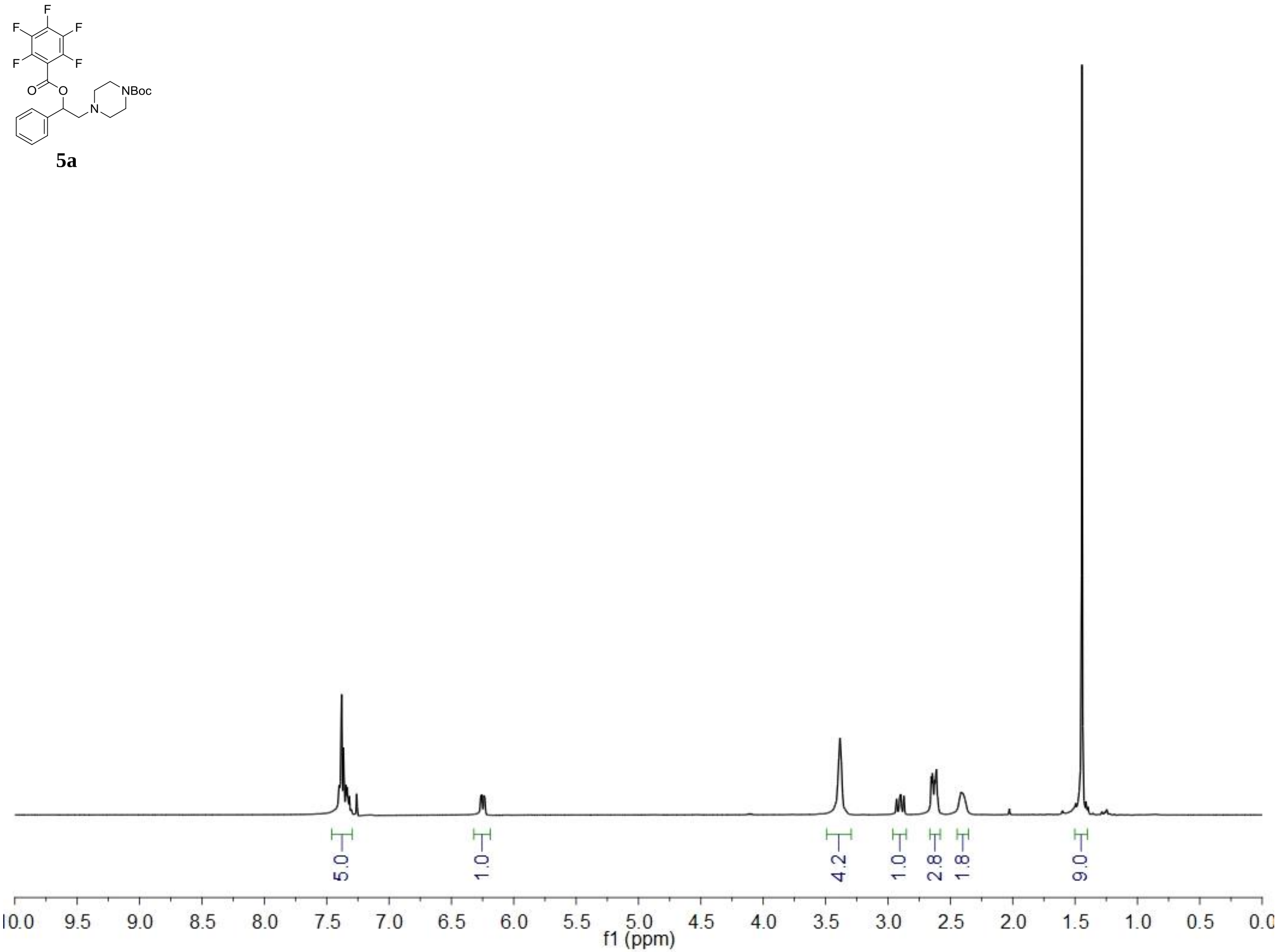
F
F
F
F
F
O
O
N
NBoc
5.0
1.0
4.2
1.0
2.8
1.8
9.0
10.0 9.5 9.0 8.5 8.0 7.5 7.0 6.5 6.0 5.5 5.0 4.5 4.0 3.5 3.0 2.5 2.0 1.5 1.0 0.5 0.0
f1 (ppm)

5a

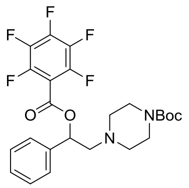

5a

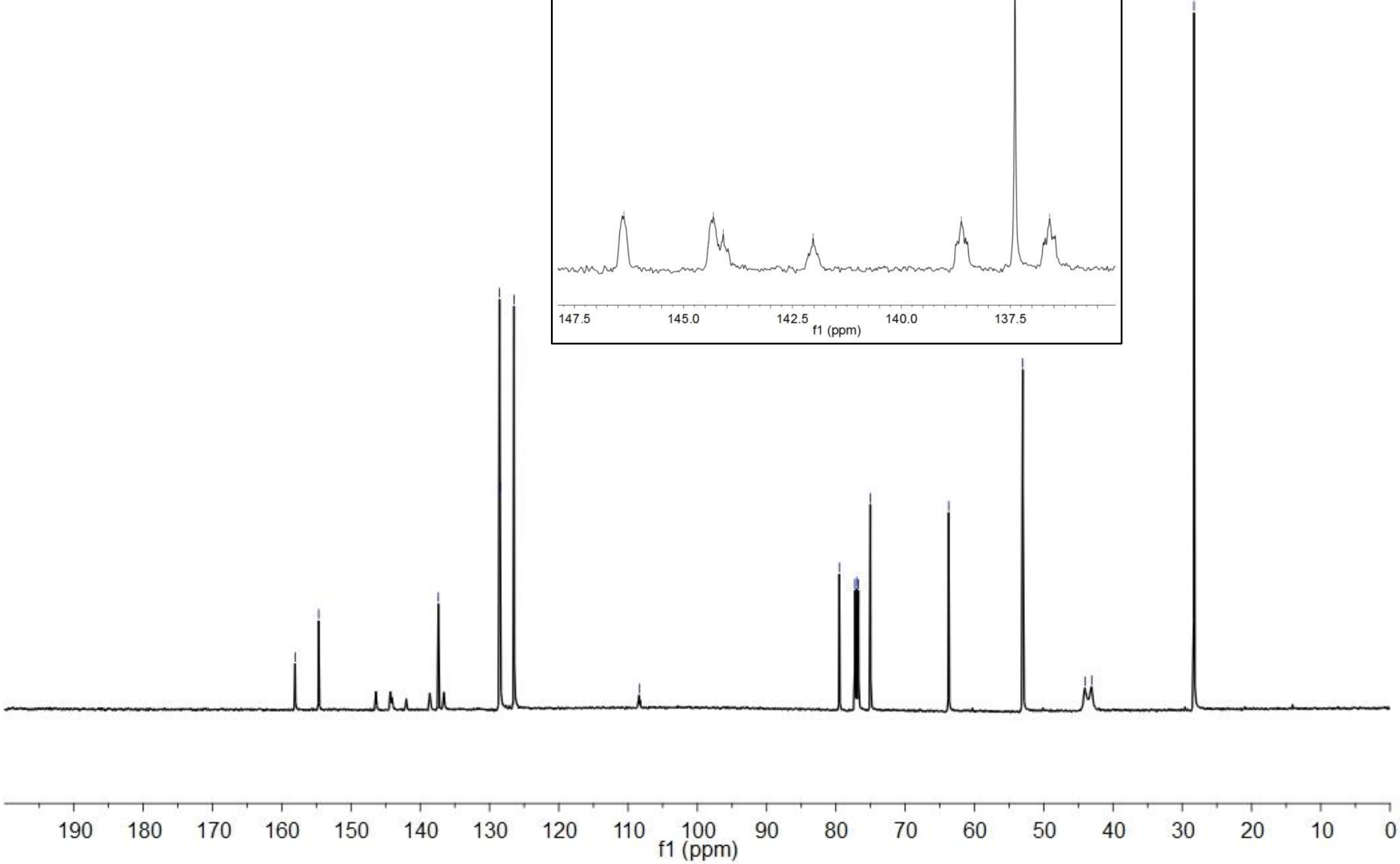

158.1
154.7
137.4
128.6
128.5
126.5
108.4
79.5
77.3
77.0
76.7
75.0
63.7
53.0
44.0
43.1
28.3
146.4
144.3
144.1
142.0
138.6
137.4
136.6
147.5 145.0 142.5 140.0 137.5
f1 (ppm)
190 180 170 160 150 140 130 120 110 100 90 80 70 60 50 40 30 20 10 0
f1 (ppm)

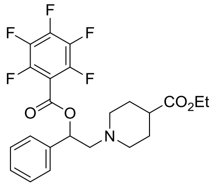
F
F
F
F
F
O
O
N
CO_2Et

5b

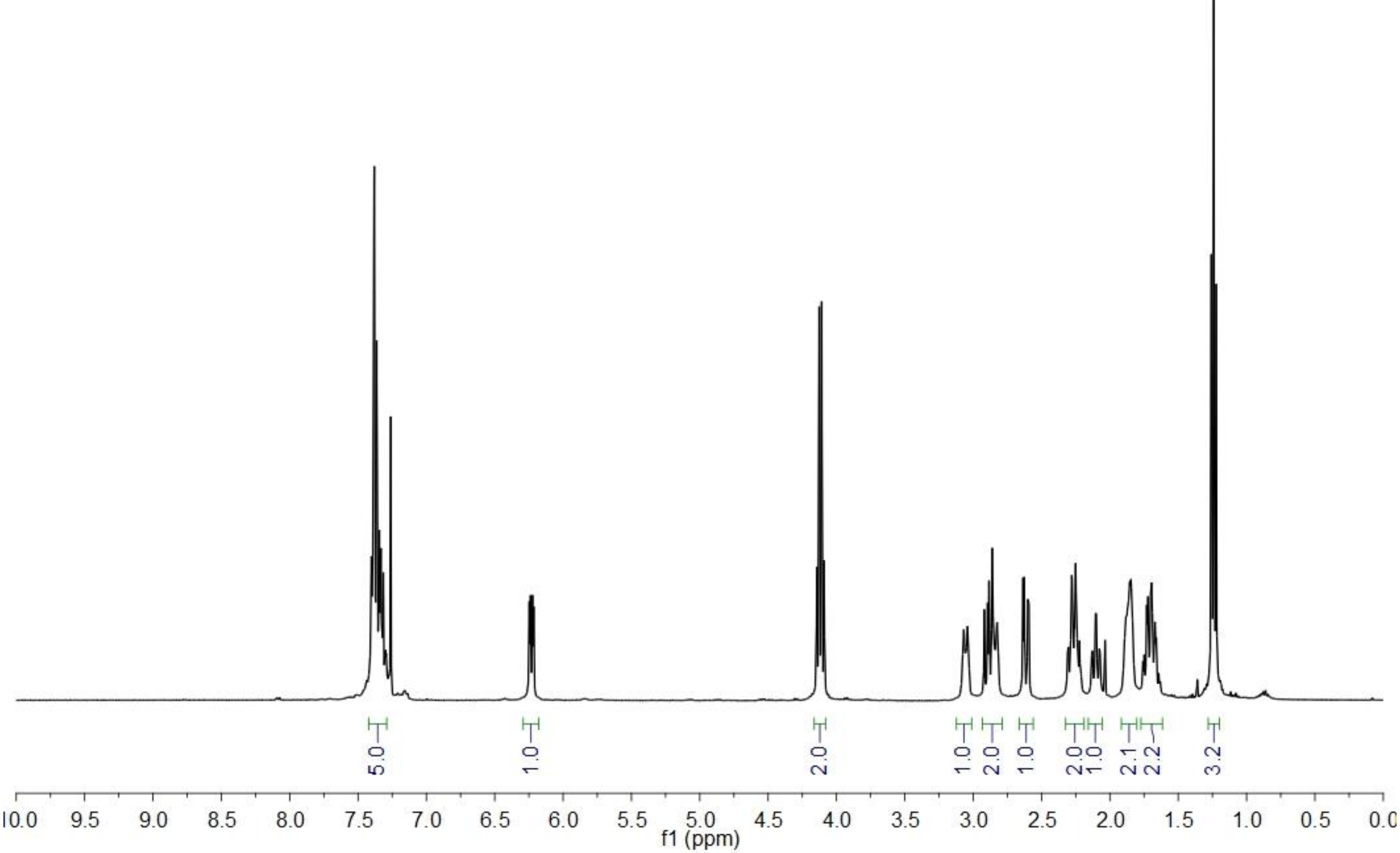
5.0
1.0
2.0
1.0
2.0
1.0
2.0
1.0
2.1
2.2
3.2
10.0 9.5 9.0 8.5 8.0 7.5 7.0 6.5 6.0 5.5 5.0 4.5 4.0 3.5 3.0 2.5 2.0 1.5 1.0 0.5 0.0
f1 (ppm)

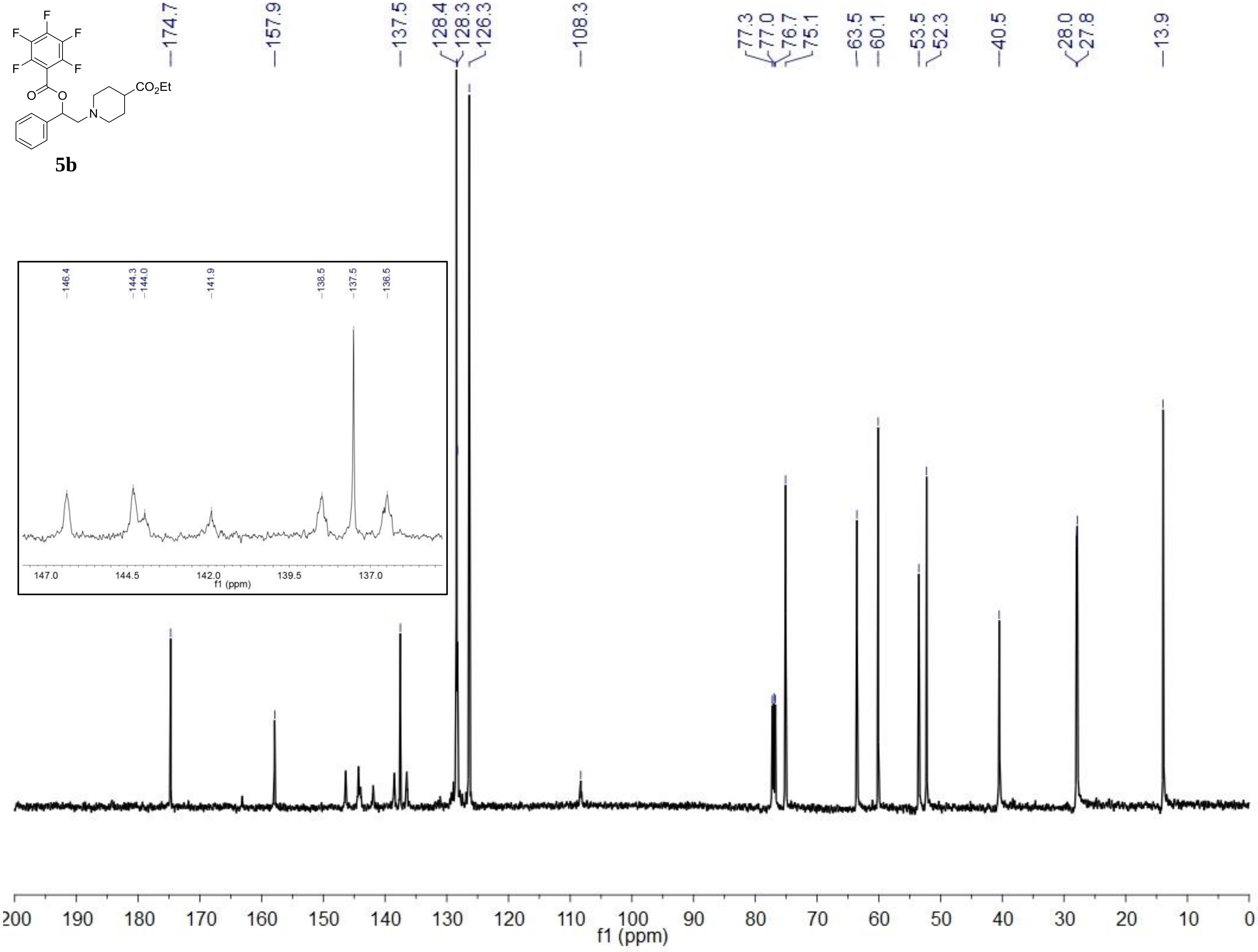
174.7
157.9
137.5
128.4
128.3
126.3
108.3
77.3
77.0
76.7
75.1
63.5
60.1
53.5
52.3
40.5
28.0
27.8
13.9
5b
CO2Et
146.4
144.3
144.0
141.9
138.5
137.5
136.5
f1 (ppm)
f1 (ppm)

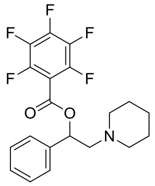
F
F
F
F
F
O
O
N

5c

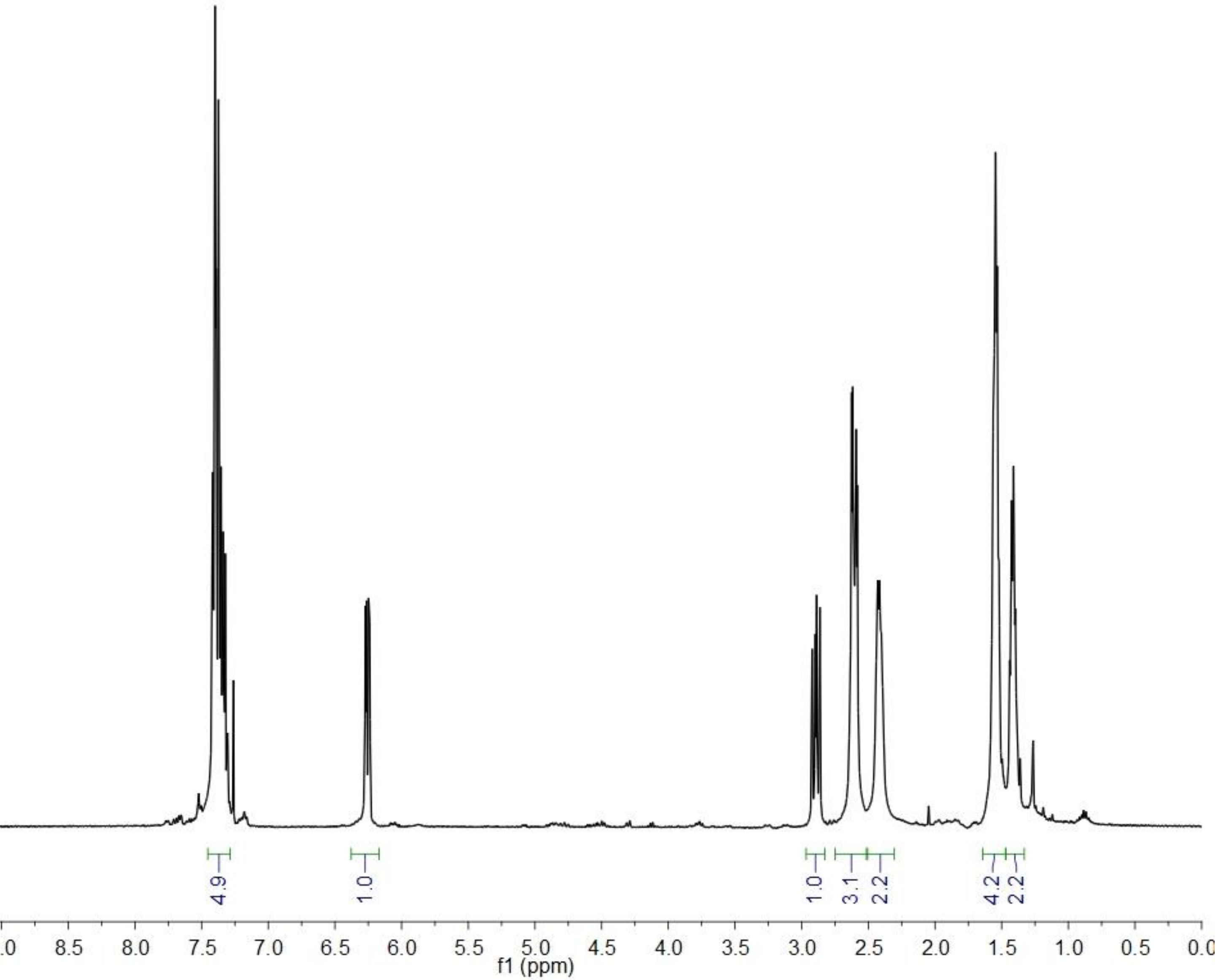
4.9
1.0
1.0
3.1
2.2
4.2
2.2
10.0
9.5
9.0
8.5
8.0
7.5
7.0
6.5
6.0
5.5
5.0
4.5
4.0
3.5
3.0
2.5
2.0
1.5
1.0
0.5
0.0
f1 (ppm)

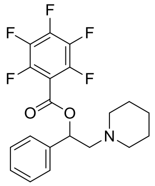

5c

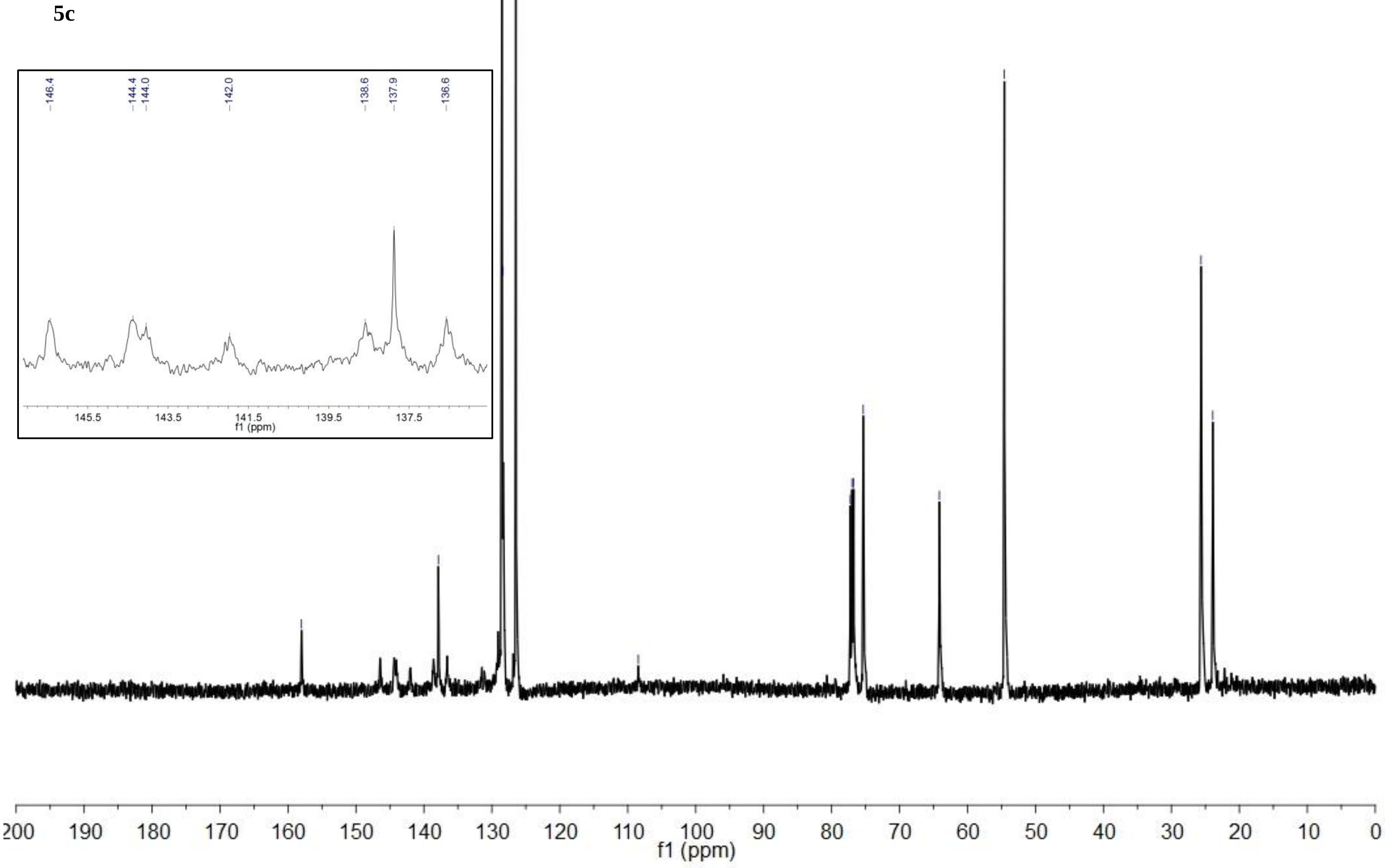

158.0
137.9
128.5
128.4
126.5
108.4
77.3
77.0
76.7
75.3
64.1
54.6
25.6
23.9
146.4
144.4
144.0
142.0
138.6
137.9
136.6
145.5
143.5
141.5
139.5
137.5
f1 (ppm)
200 190 180 170 160 150 140 130 120 110 100 90 80 70 60 50 40 30 20 10 0
f1 (ppm)

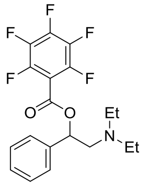

5d

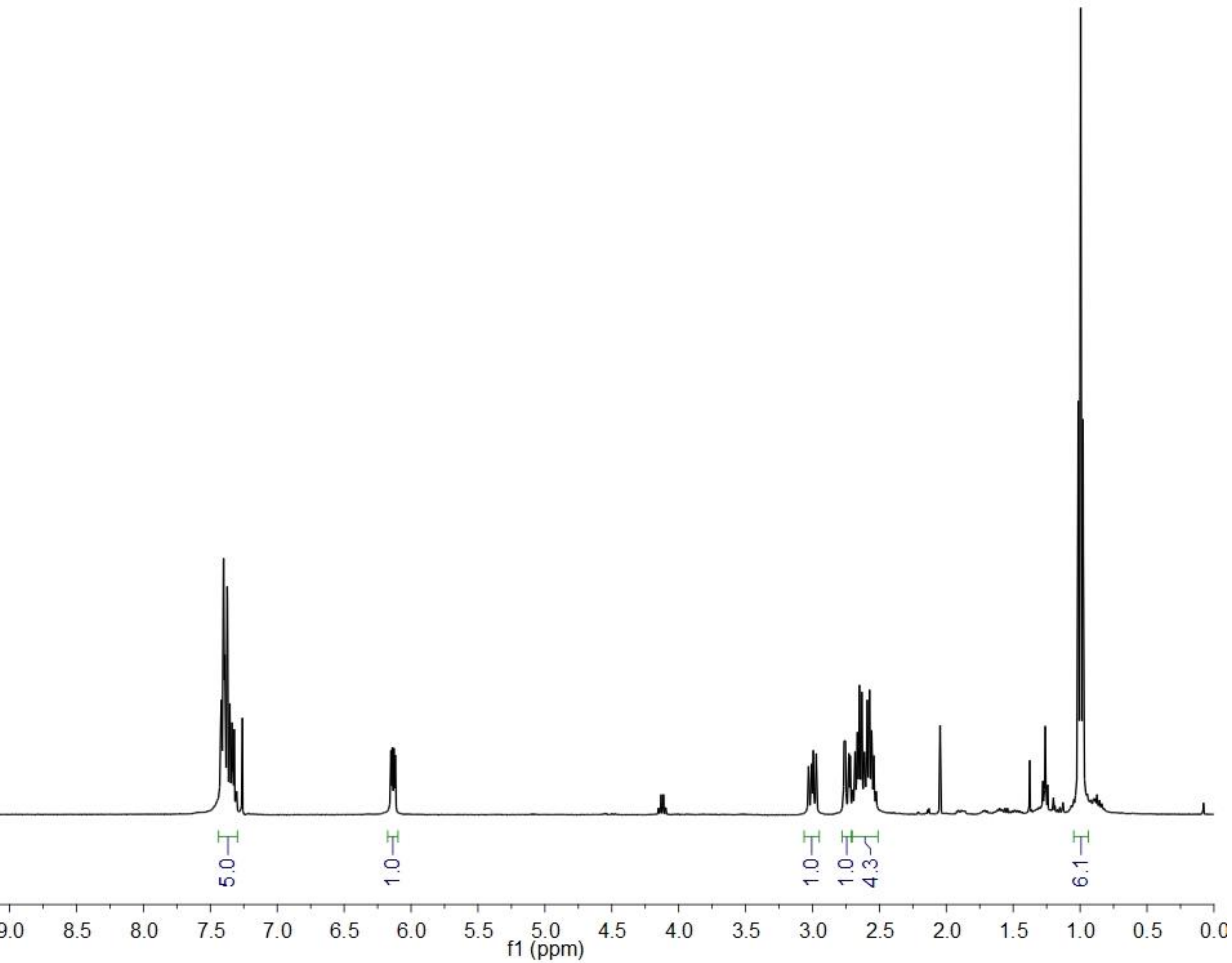
5.0
1.0
1.0
1.0
4.3
6.1
10.0 9.5 9.0 8.5 8.0 7.5 7.0 6.5 6.0 5.5 5.0 4.5 4.0 3.5 3.0 2.5 2.0 1.5 1.0 0.5 0.0
f1 (ppm)

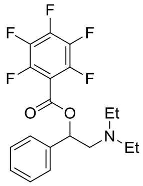
F
F
F
F
F
O
O
N
Et
Et

5d

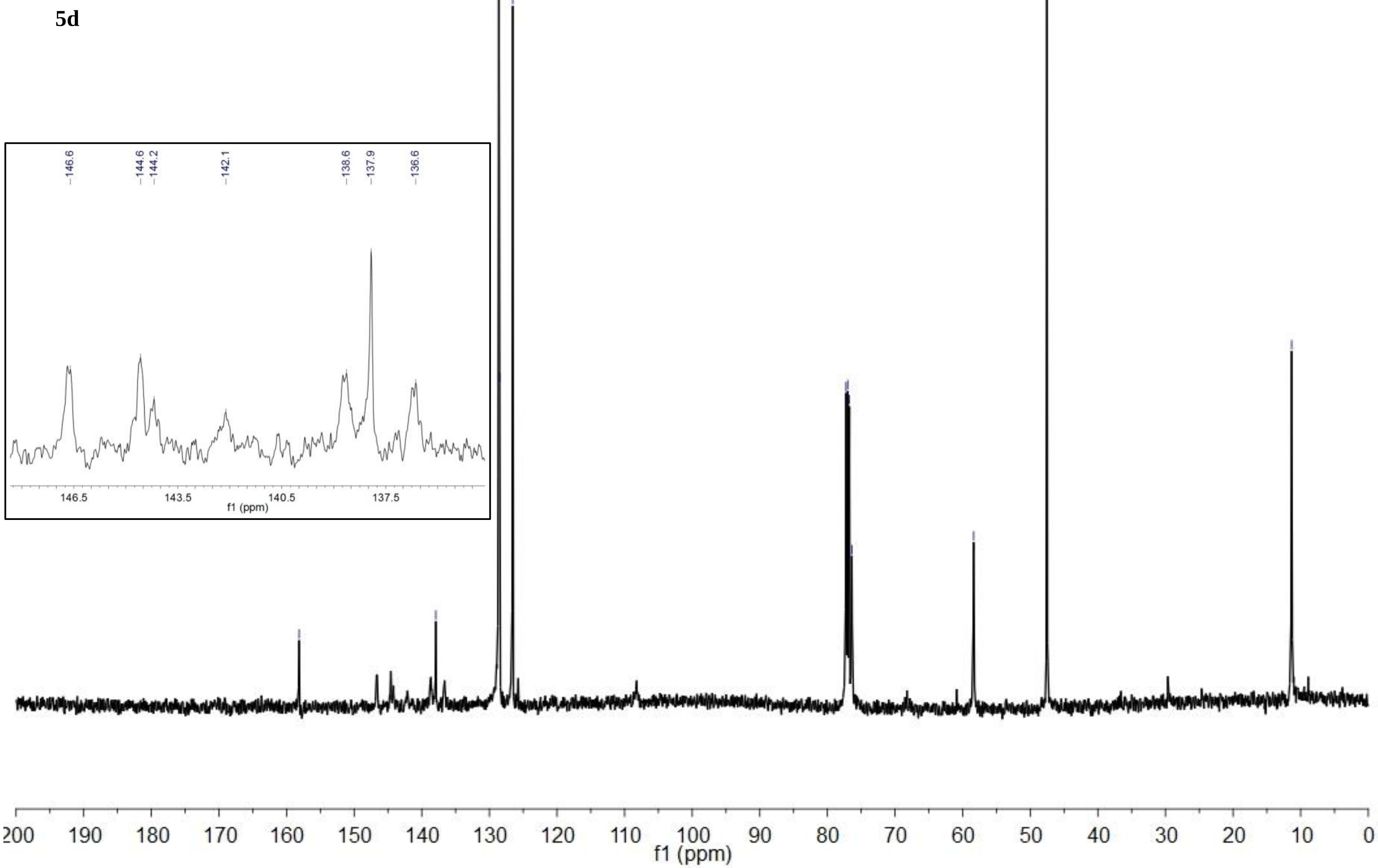
158.1
137.9
128.6
128.5
126.5
77.3
77.0
76.7
76.4
58.4
47.5
11.3
146.6
144.6
144.2
142.1
138.6
137.9
136.6
146.5
143.5
140.5
137.5
f1 (ppm)
200
190
180
170
160
150
140
130
120
110
100
90
80
70
60
50
40
30
20
10
0
f1 (ppm)

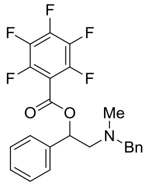
F
F
F
F
F
O
O
Me
N
Bn

5e

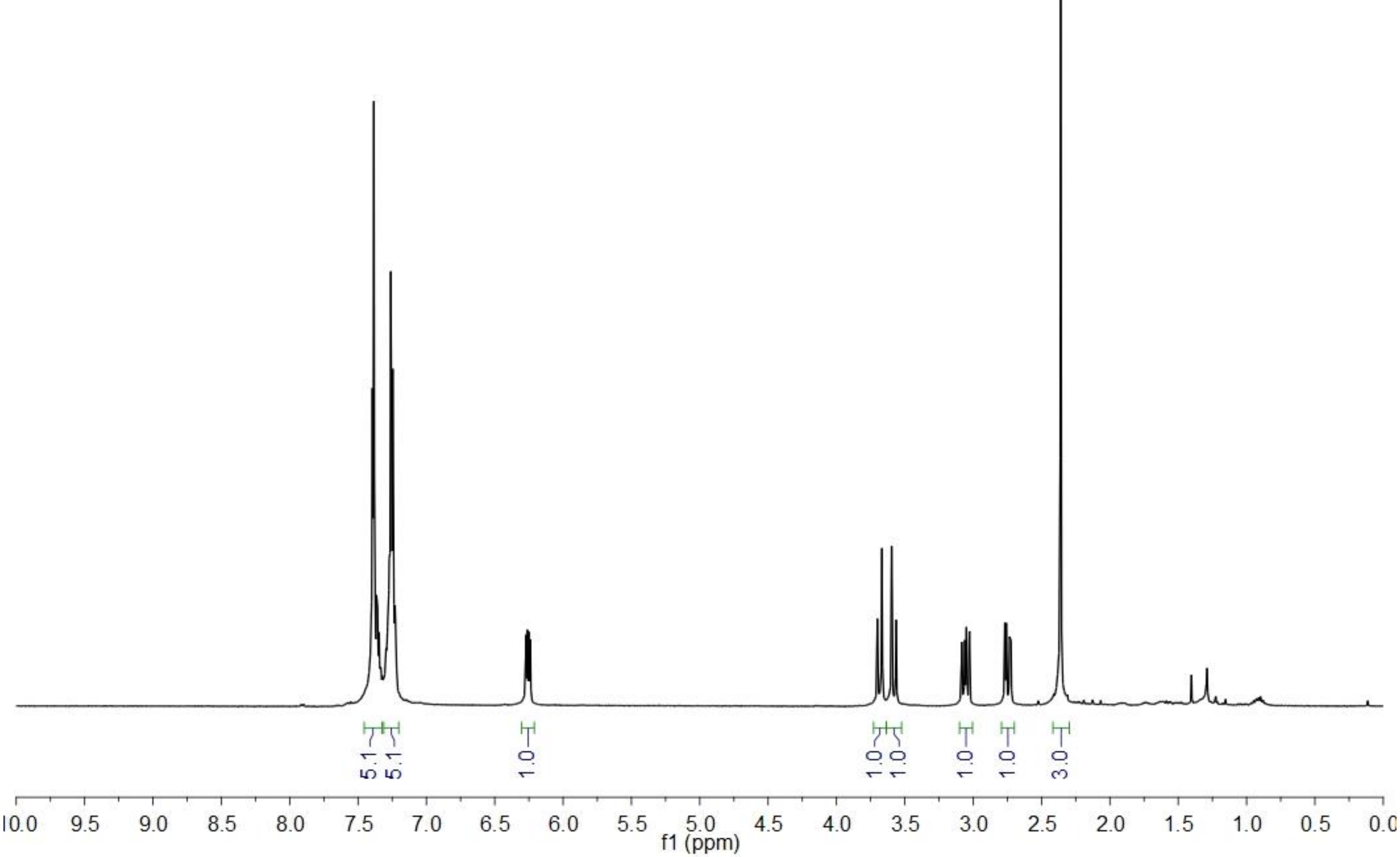
5.1
5.1
1.0
1.0
1.0
1.0
1.0
3.0
10.0 9.5 9.0 8.5 8.0 7.5 7.0 6.5 6.0 5.5 5.0 4.5 4.0 3.5 3.0 2.5 2.0 1.5 1.0 0.5 0.0
f1 (ppm)

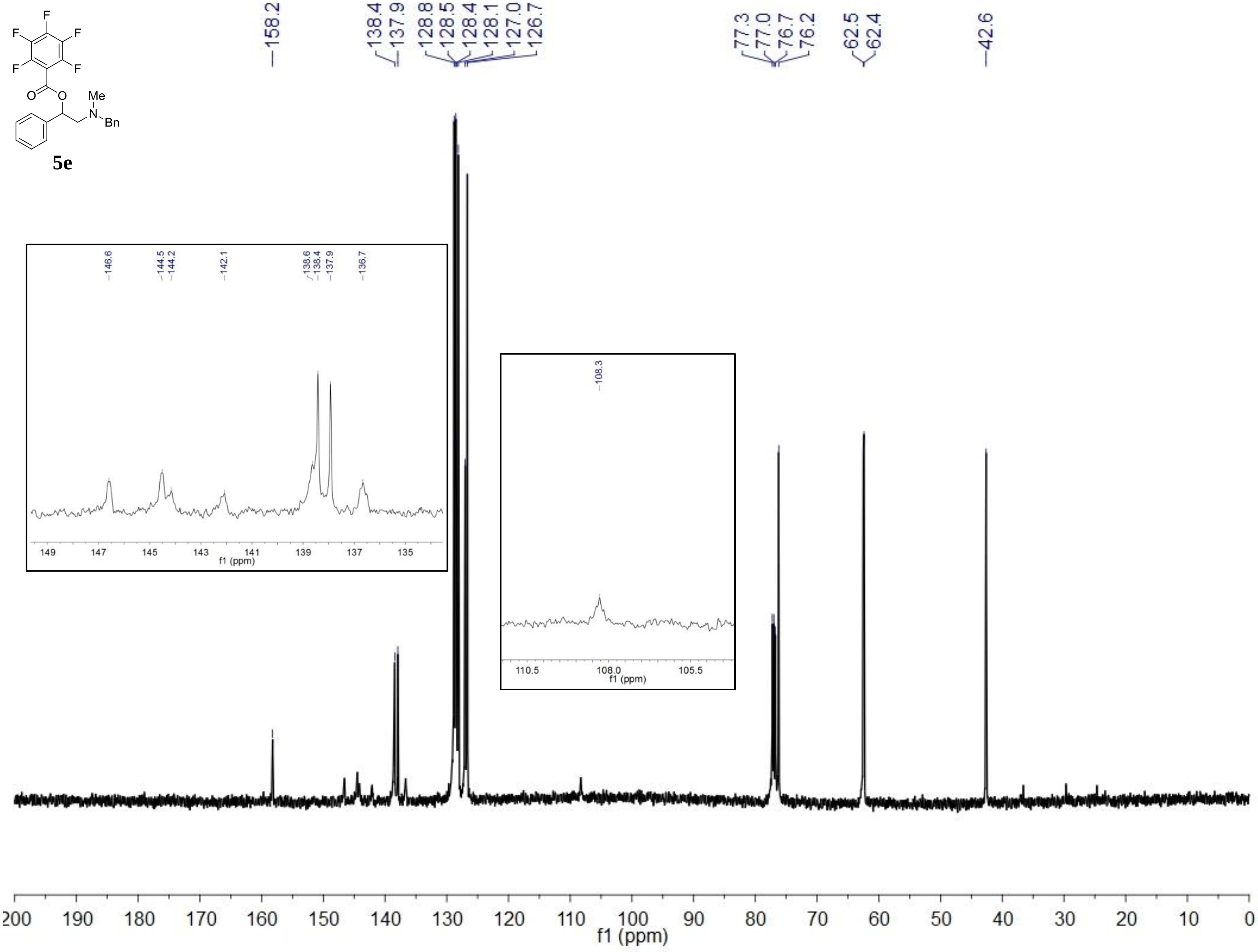

F
F
F
F
F
O
O
Me
N
Bn
5e
158.2
138.4
137.9
128.8
128.5
128.4
128.1
127.0
126.7
77.3
77.0
76.7
76.2
62.5
62.4
42.6
146.6
144.5
144.2
142.1
138.6
138.4
137.9
136.7
149 147 145 143 141 139 137 135
f1 (ppm)
108.3
110.5 108.0 105.5
f1 (ppm)
200 190 180 170 160 150 140 130 120 110 100 90 80 70 60 50 40 30 20 10 0
f1 (ppm)

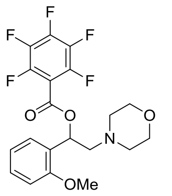
F
F
F
F
F
O
O
N
O
OMe

6a

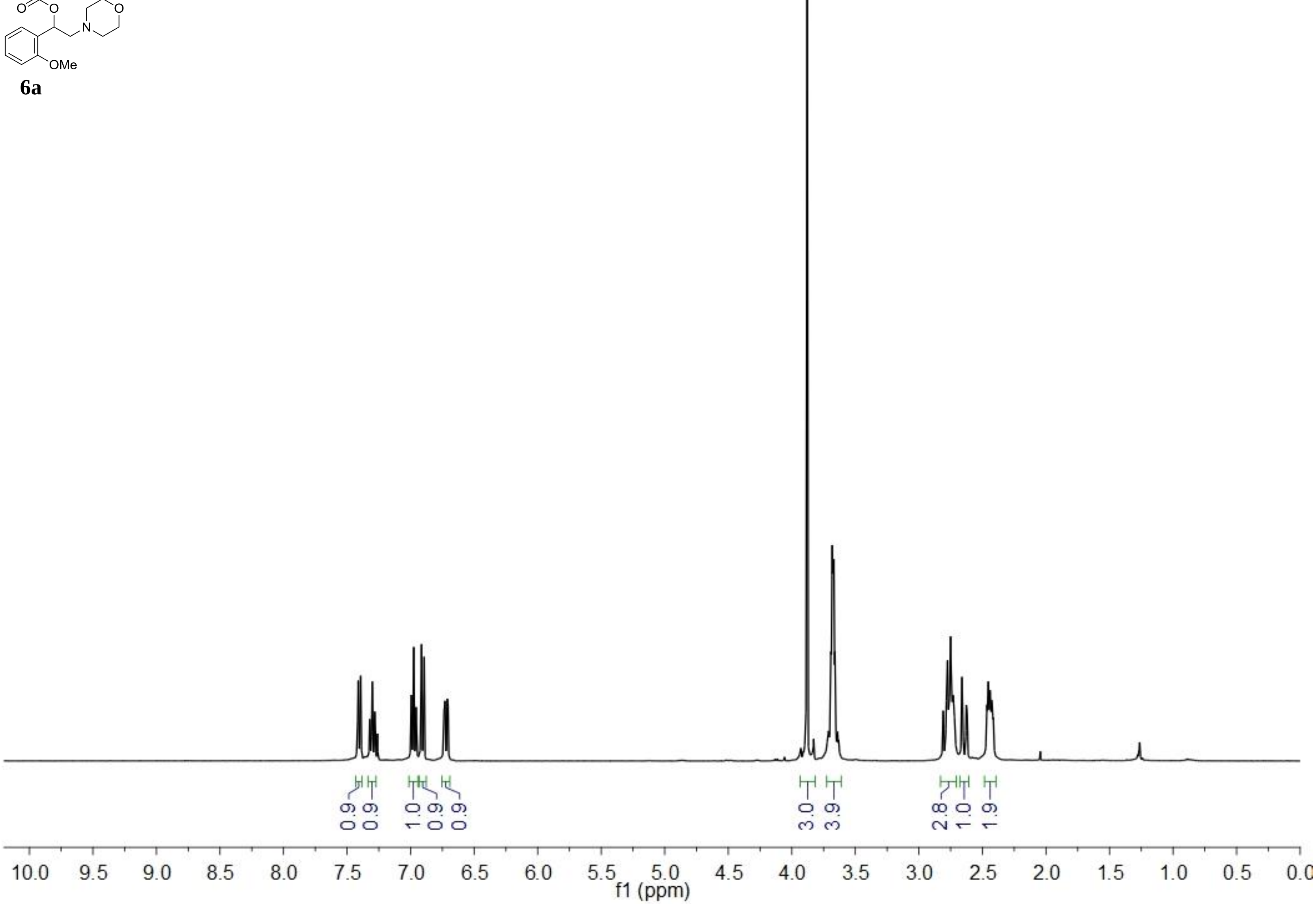
0.9
0.9
1.0
0.9
0.9
3.0
3.9
2.8
1.0
1.9
10.0 9.5 9.0 8.5 8.0 7.5 7.0 6.5 6.0 5.5 5.0 4.5 4.0 3.5 3.0 2.5 2.0 1.5 1.0 0.5 0.0
f1 (ppm)

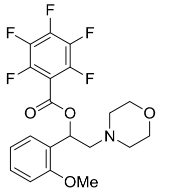

F
F
F
F
F
O
O
N
O
OMe

6a

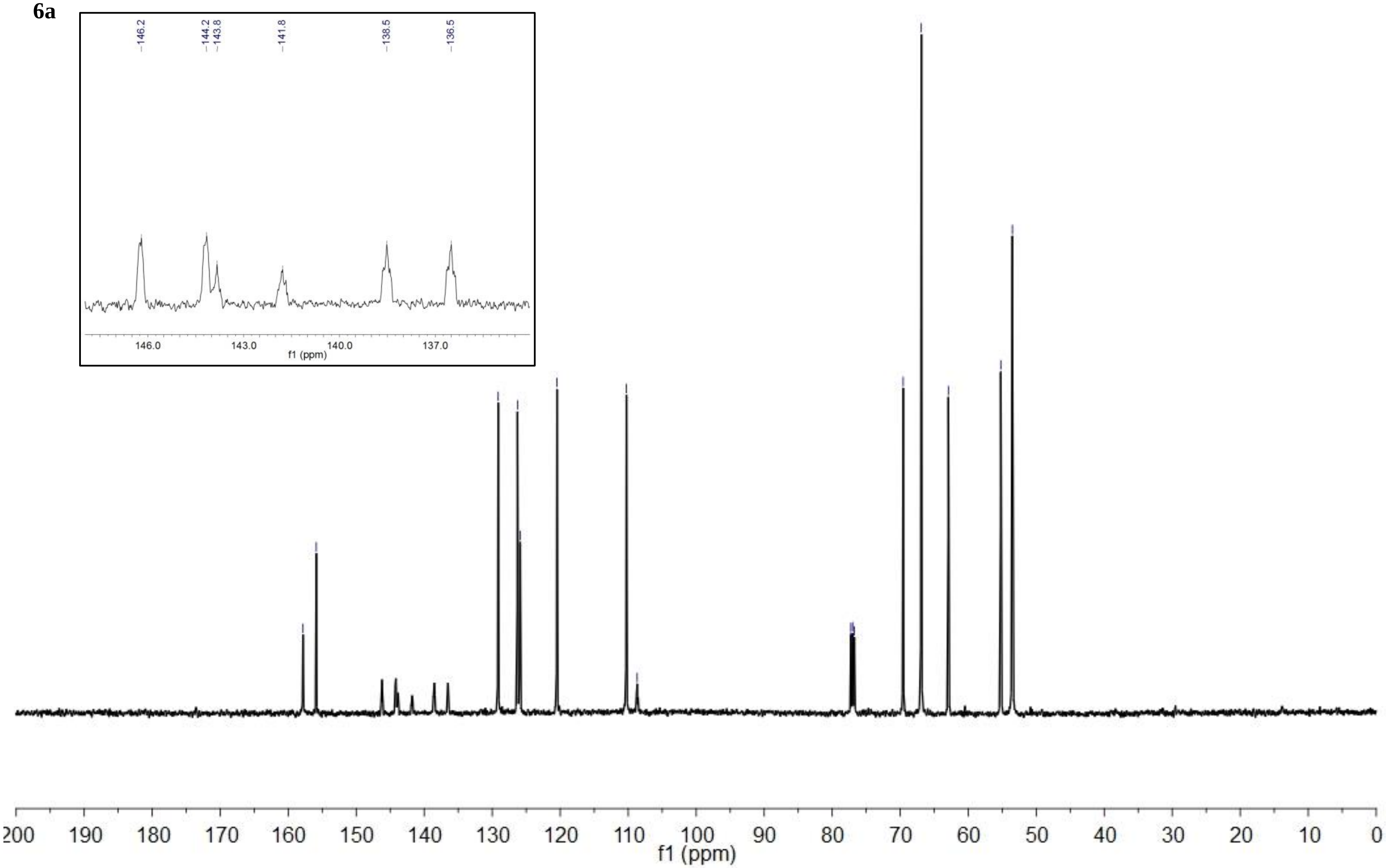

157.8
155.9
129.1
126.3
125.9
120.5
110.3
108.7
77.3
77.0
76.7
69.6
66.9
62.9
55.2
53.5
146.2
144.2
143.8
141.8
138.5
136.5
146.0
143.0
140.0
137.0
f1 (ppm)
200
190
180
170
160
150
140
130
120
110
100
90
80
70
60
50
40
30
20
10
0
f1 (ppm)

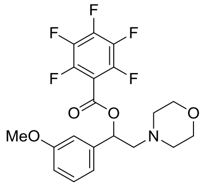

F
F
F
F
F
O
O
MeO
N
O

6b

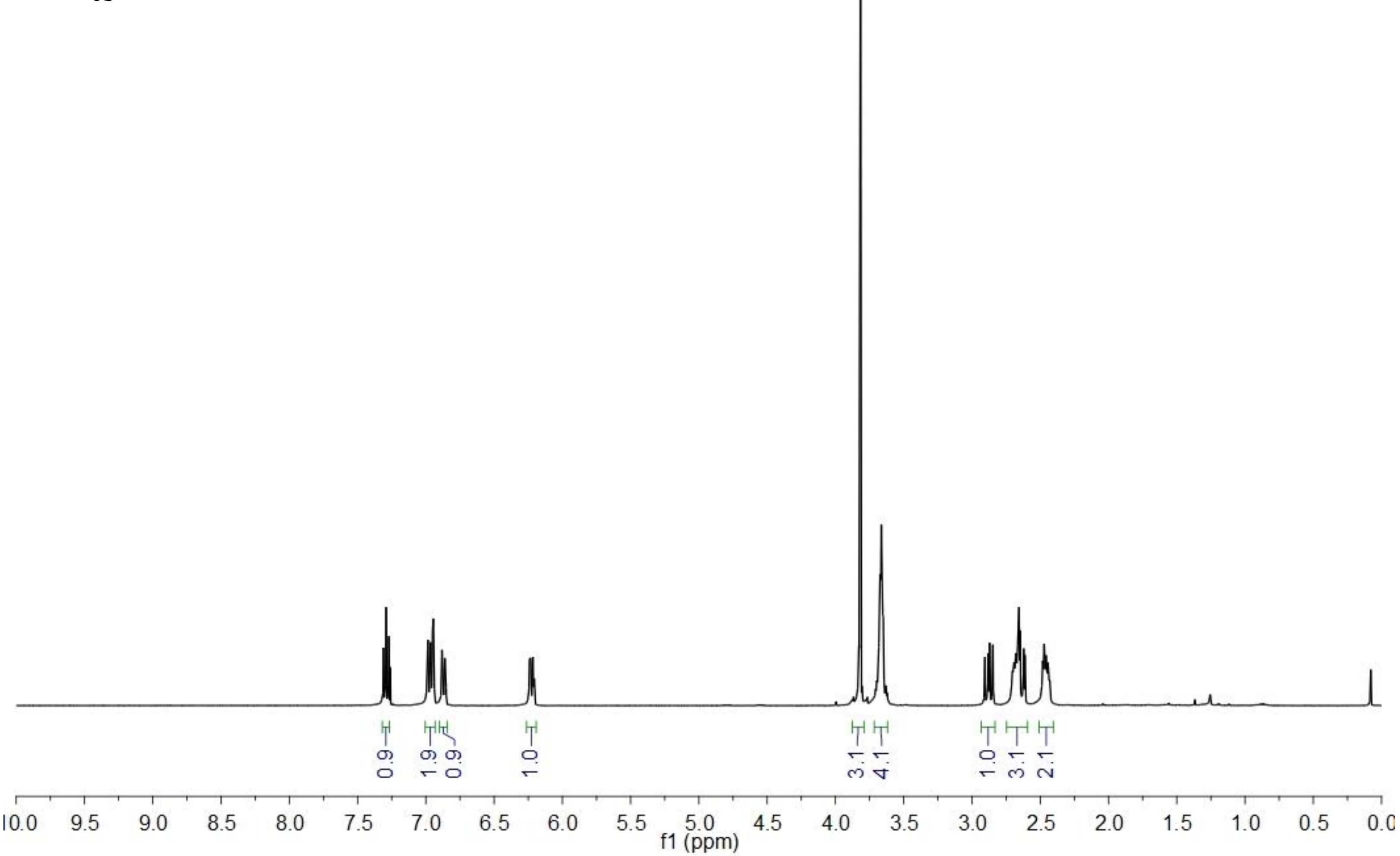

0.9
1.9
0.9
1.0
3.1
4.1
1.0
3.1
2.1
10.0 9.5 9.0 8.5 8.0 7.5 7.0 6.5 6.0 5.5 5.0 4.5 4.0 3.5 3.0 2.5 2.0 1.5 1.0 0.5 0.0
f1 (ppm)

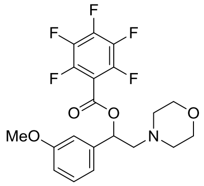
F
F
F
F
F
O
O
N
O
MeO

6b

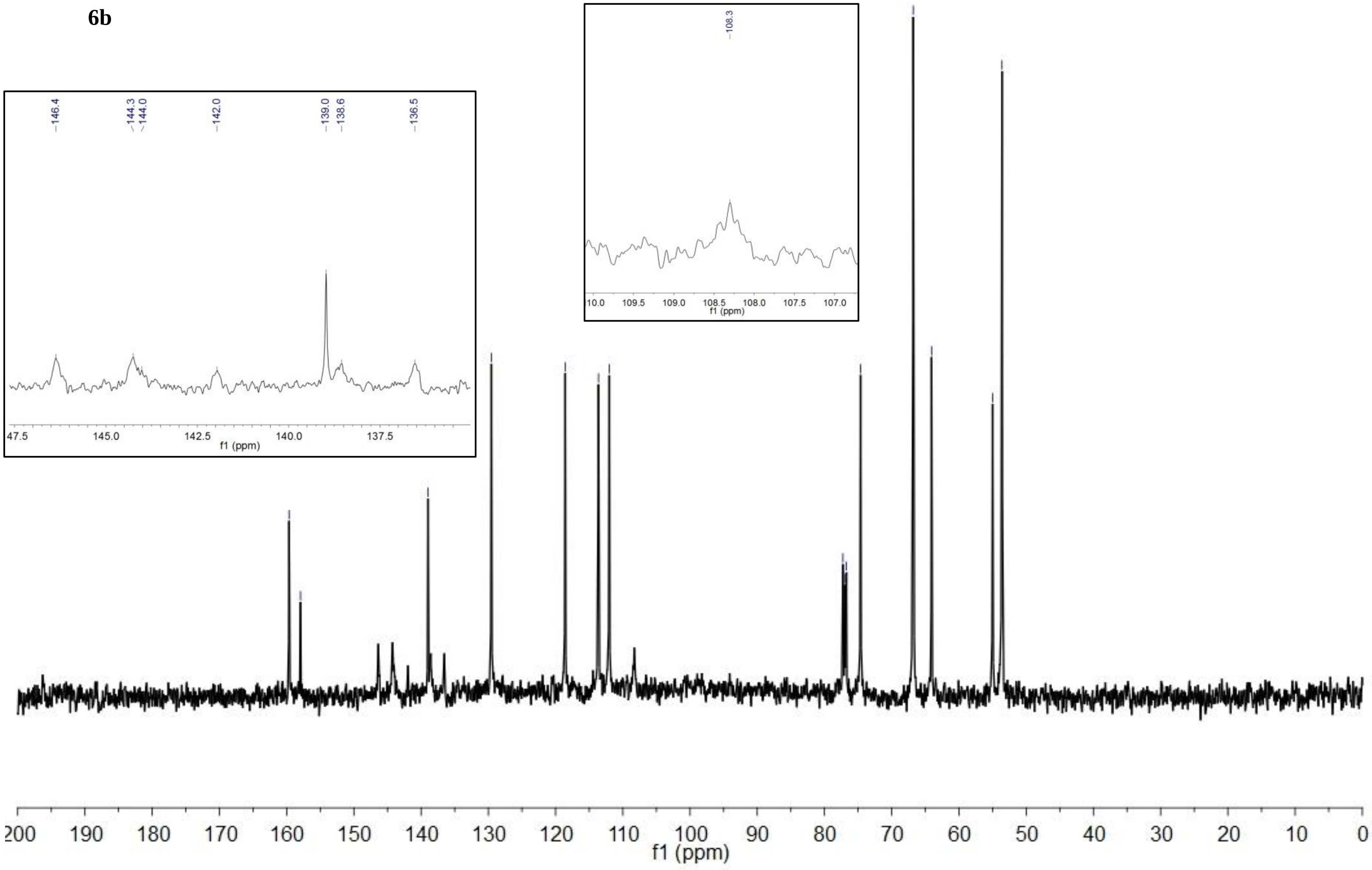
159.6
157.9
139.0
129.5
118.6
113.6
112.0
77.3
77.0
76.7
74.6
66.8
64.1
55.0
53.6
146.4
144.3
144.0
142.0
139.0
138.6
136.5
108.3
f1 (ppm)

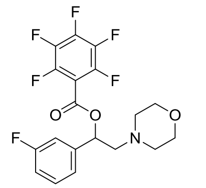

6c

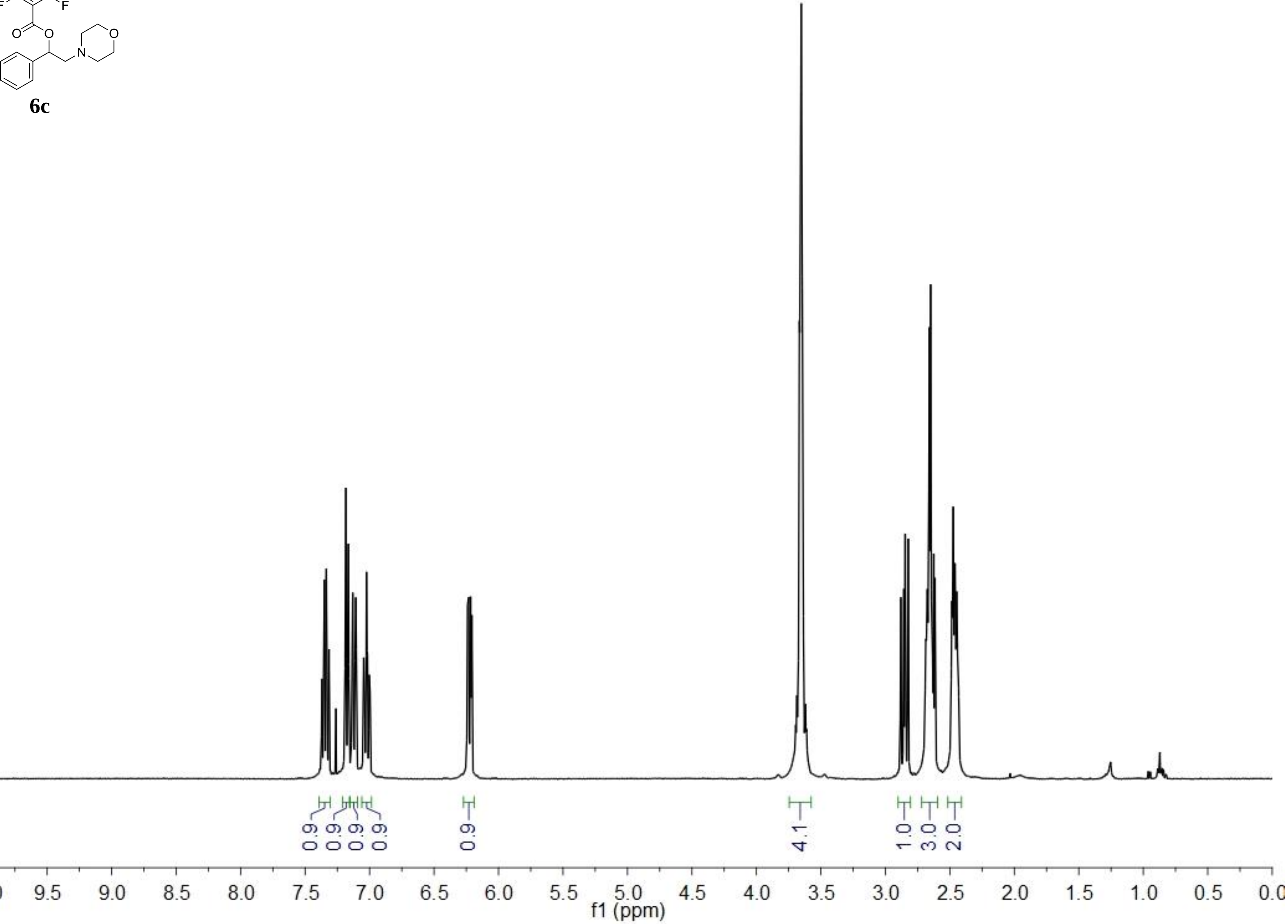
0.9
0.9
0.9
0.9
0.9
4.1
1.0
3.0
2.0
10.0 9.5 9.0 8.5 8.0 7.5 7.0 6.5 6.0 5.5 5.0 4.5 4.0 3.5 3.0 2.5 2.0 1.5 1.0 0.5 0.0
f1 (ppm)

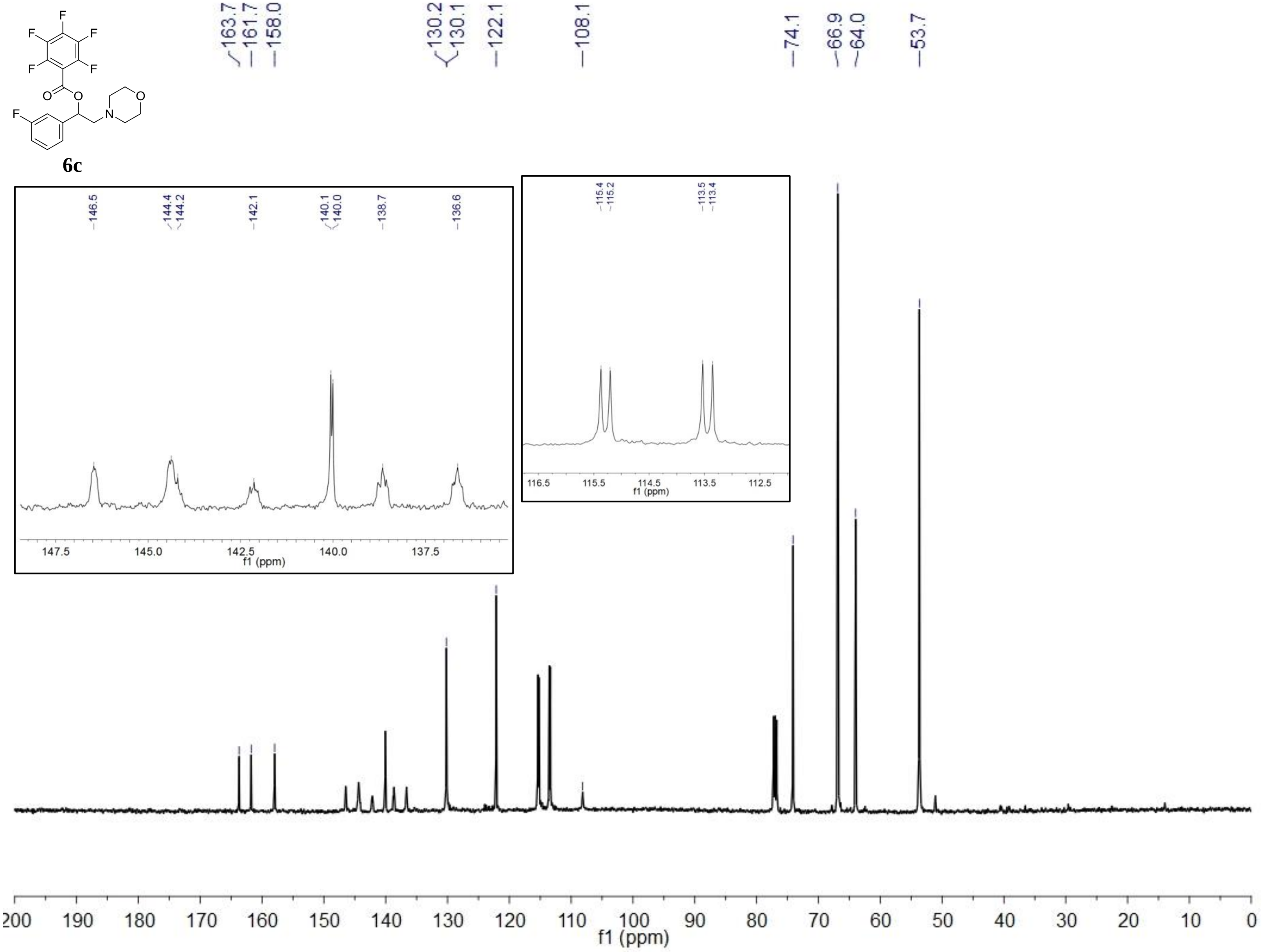
6c
163.7
161.7
158.0
130.2
130.1
122.1
108.1
74.1
66.9
64.0
53.7
146.5
144.4
144.2
142.1
140.1
140.0
138.7
136.6
147.5
145.0
142.5
140.0
137.5
f1 (ppm)
115.4
115.2
113.5
113.4
116.5
115.5
114.5
113.5
112.5
f1 (ppm)
200 190 180 170 160 150 140 130 120 110 100 90 80 70 60 50 40 30 20 10 0
f1 (ppm)

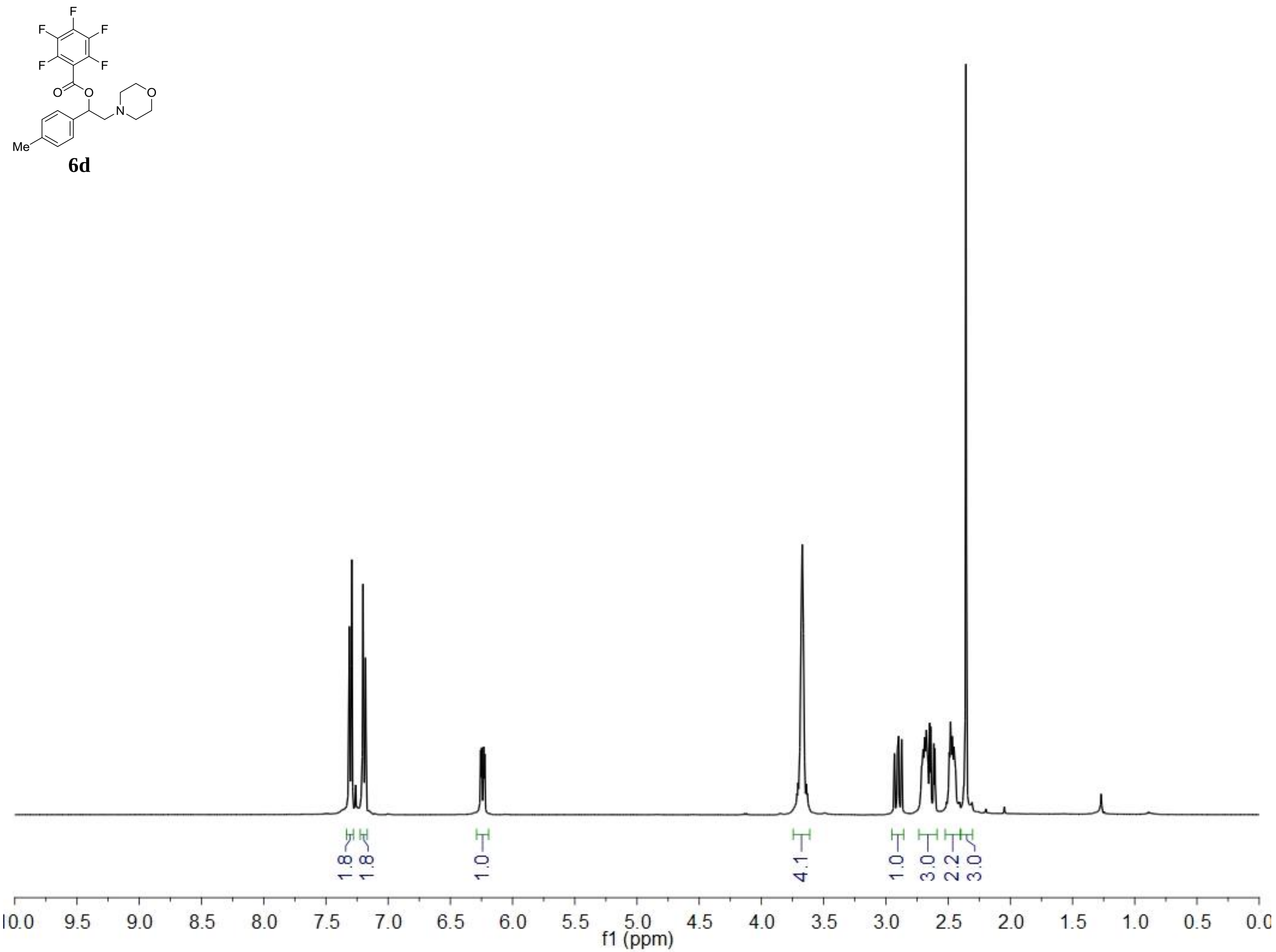
F
F
F
F
F
O
O
N
O
Me
6d
1.8
1.8
1.0
4.1
1.0
3.0
2.2
3.0
10.0
9.5
9.0
8.5
8.0
7.5
7.0
6.5
6.0
5.5
5.0
4.5
4.0
3.5
3.0
2.5
2.0
1.5
1.0
0.5
0.0
f1 (ppm)

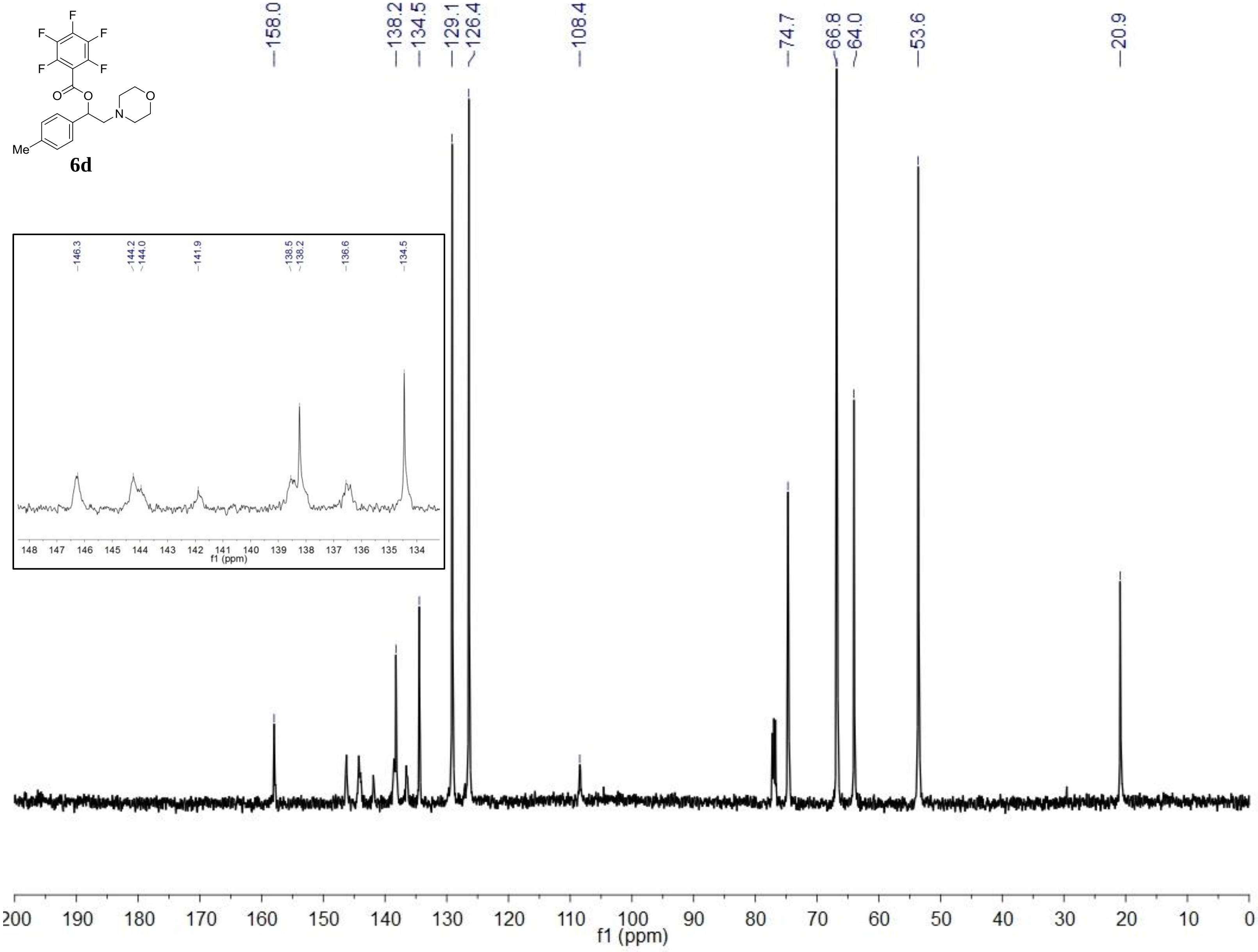
6d
158.0
138.2
134.5
129.1
126.4
108.4
74.7
66.8
64.0
53.6
20.9
146.3
144.2
144.0
141.9
138.5
138.2
136.6
134.5
f1 (ppm)

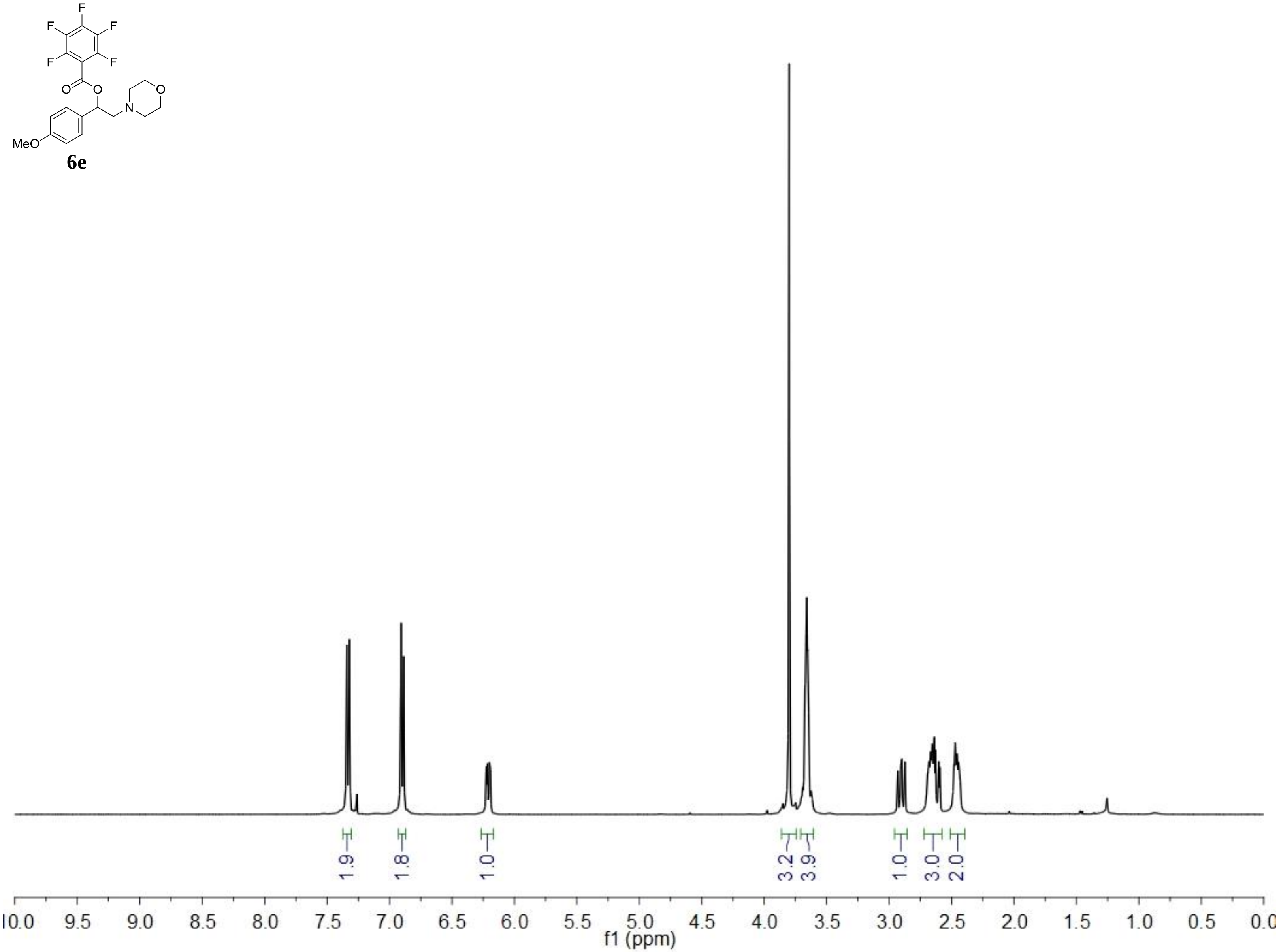
F
F
F
F
F
O
O
N
O
MeO
6e
1.9
1.8
1.0
3.2
3.9
1.0
3.0
2.0
10.0 9.5 9.0 8.5 8.0 7.5 7.0 6.5 6.0 5.5 5.0 4.5 4.0 3.5 3.0 2.5 2.0 1.5 1.0 0.5 0.0
f1 (ppm)

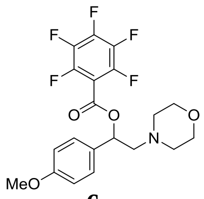

6e

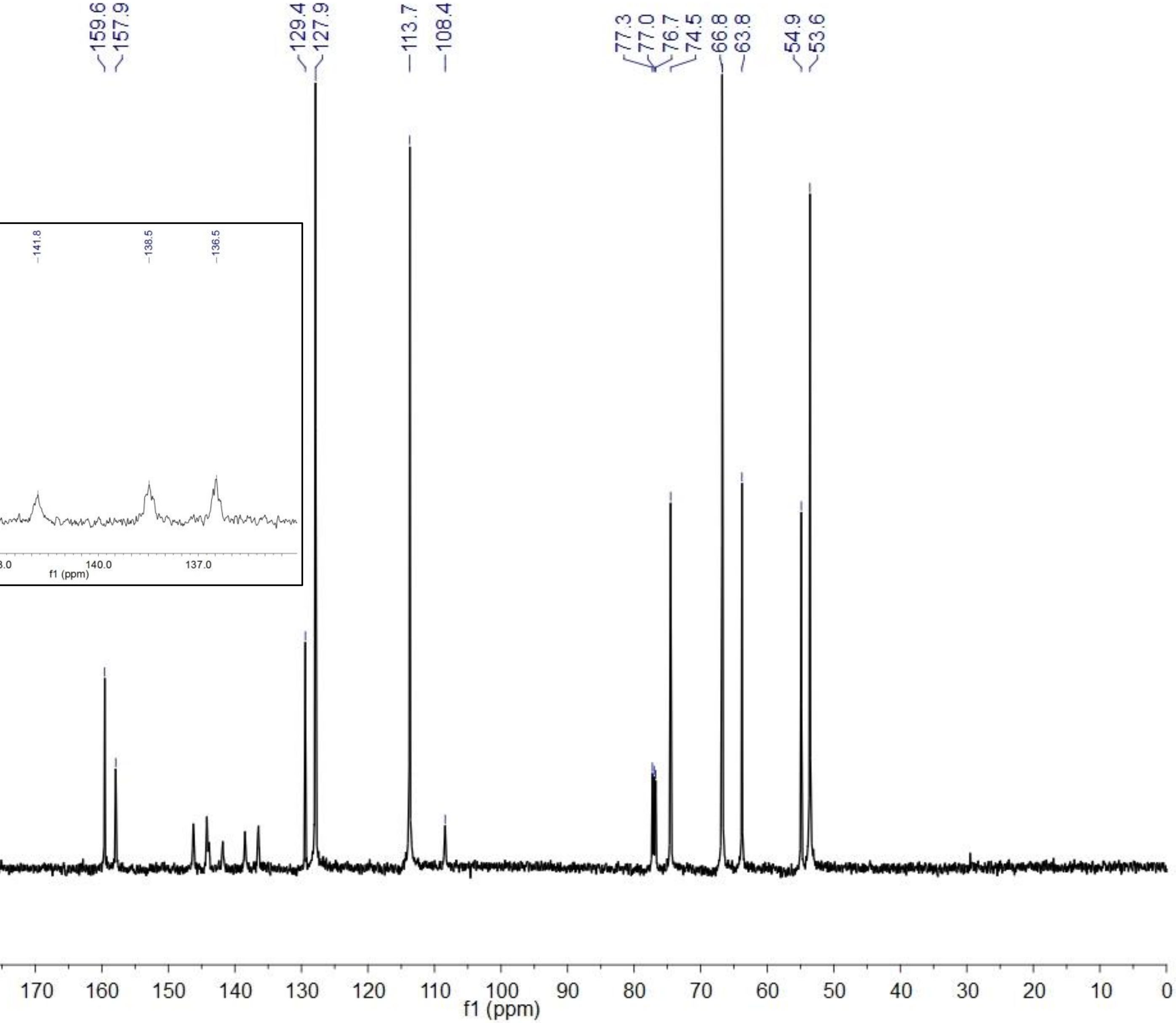

159.6
157.9
129.4
127.9
113.7
108.4
77.3
77.0
76.7
74.5
66.8
63.8
54.9
53.6
146.3
144.2
143.9
141.8
138.5
136.5
146.0
143.0
140.0
137.0
f1 (ppm)
200
190
180
170
160
150
140
130
120
110
100
90
80
70
60
50
40
30
20
10
0
f1 (ppm)

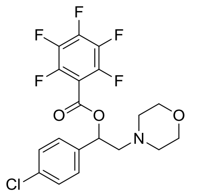
F
F
F
F
F
O
O
N
O
Cl

6f

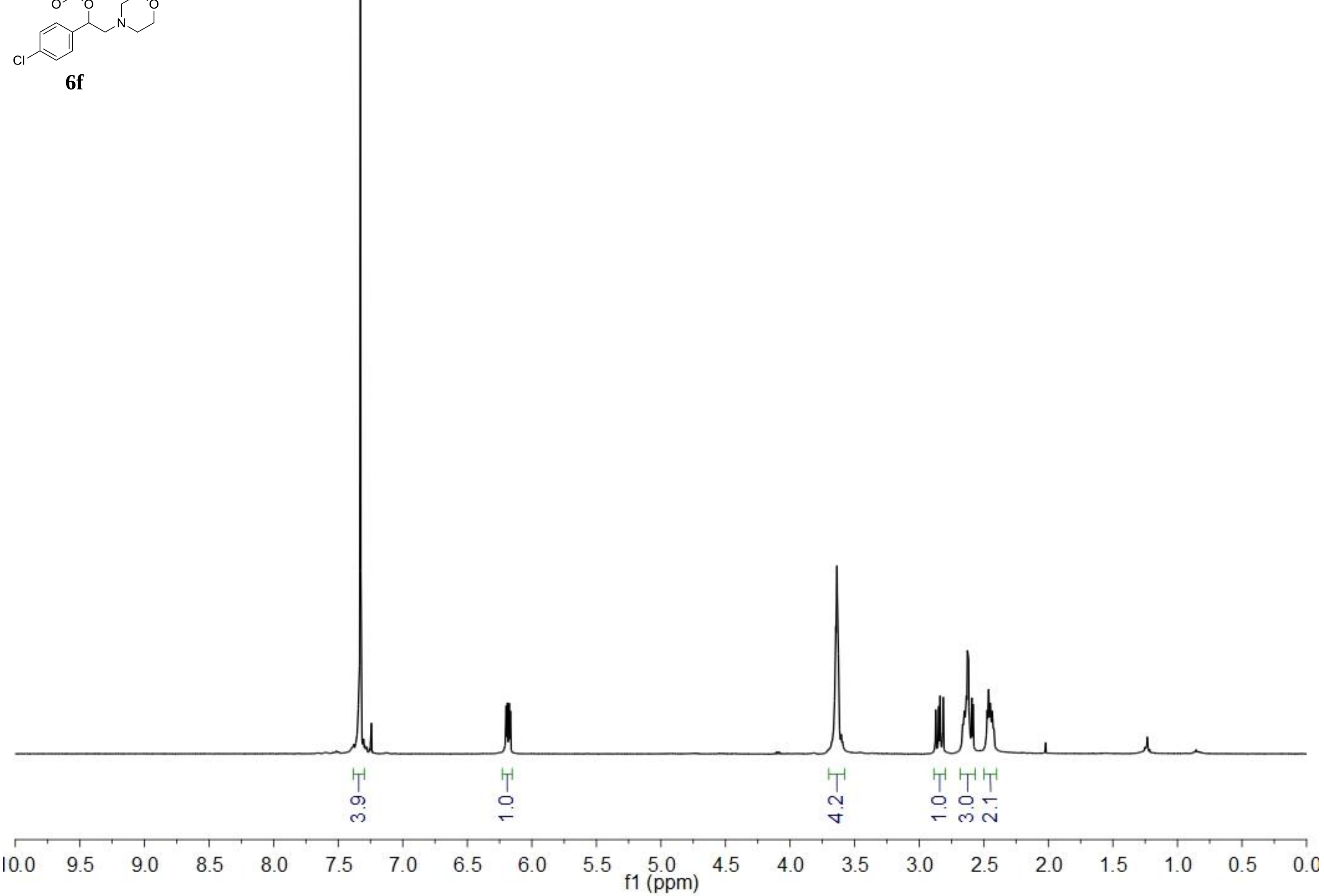
3.9
1.0
4.2
1.0
3.0
2.1
10.0 9.5 9.0 8.5 8.0 7.5 7.0 6.5 6.0 5.5 5.0 4.5 4.0 3.5 3.0 2.5 2.0 1.5 1.0 0.5 0.0
f1 (ppm)

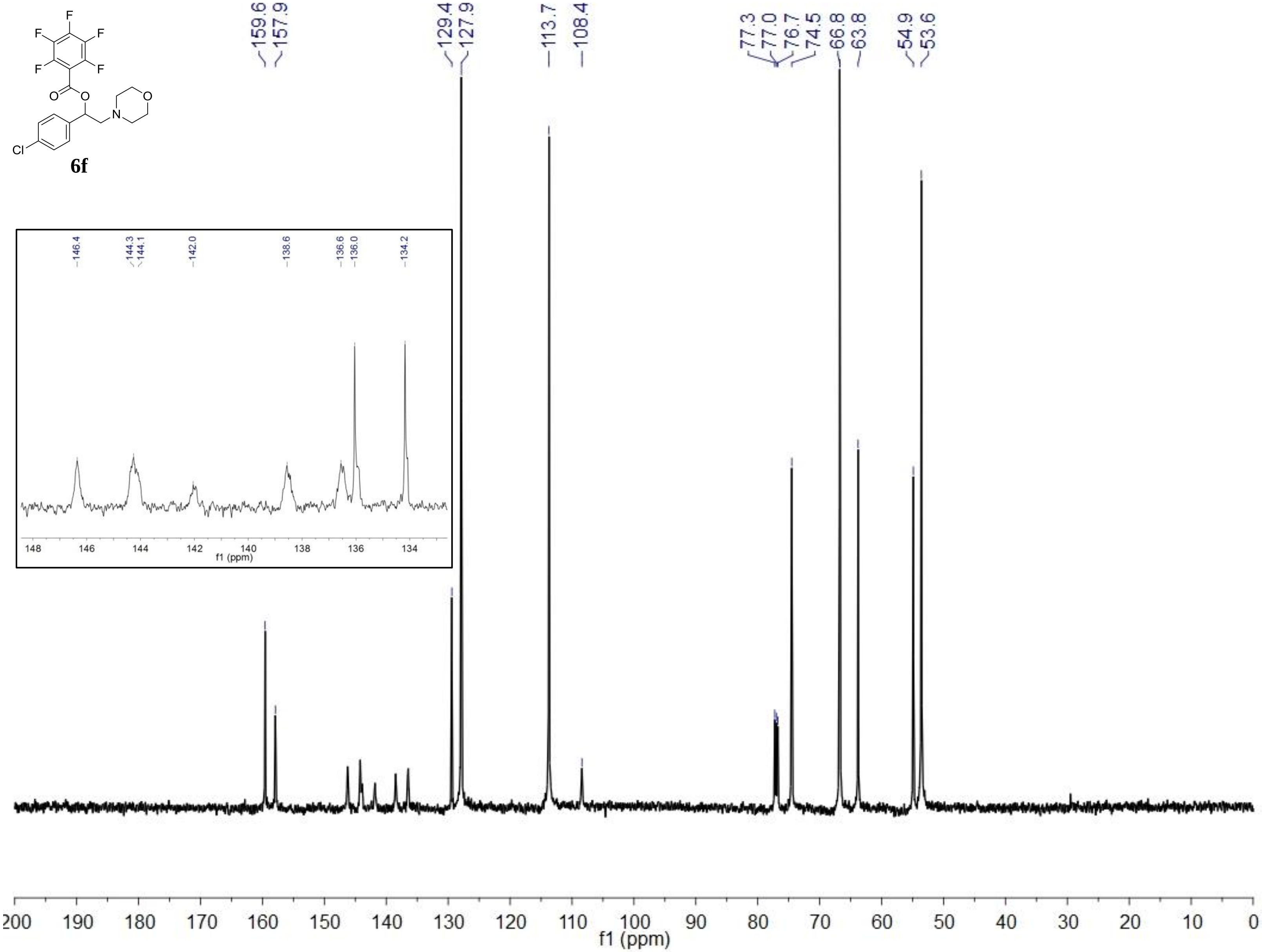
F
F
F
F
F
O
O
N
O
Cl

6f

159.6
157.9
129.4
127.9
113.7
108.4
77.3
77.0
76.7
74.5
66.8
63.8
54.9
53.6
146.4
144.3
144.1
142.0
138.6
136.6
136.0
134.2
148 146 144 142 140 138 136 134
f1 (ppm)
200 190 180 170 160 150 140 130 120 110 100 90 80 70 60 50 40 30 20 10 0
f1 (ppm)

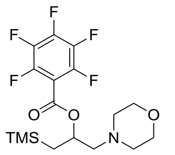

6g

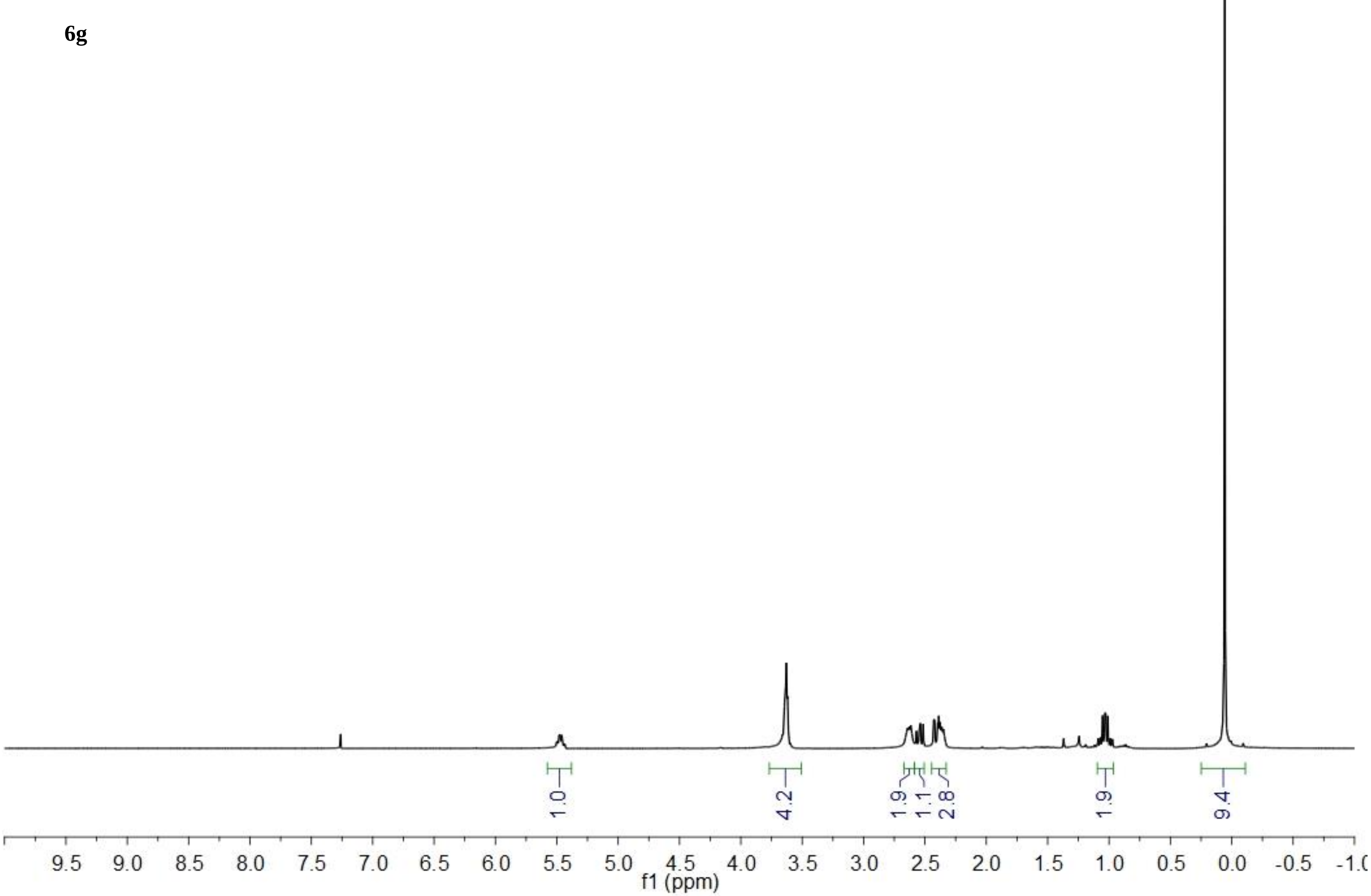

1.0
4.2
1.9
1.1
2.8
1.9
9.4
9.5 9.0 8.5 8.0 7.5 7.0 6.5 6.0 5.5 5.0 4.5 4.0 3.5 3.0 2.5 2.0 1.5 1.0 0.5 0.0 -0.5
f1 (ppm)

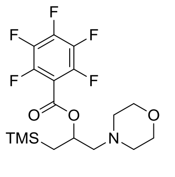
F
F
F
F
F
O
O
TMS
N
O

6g

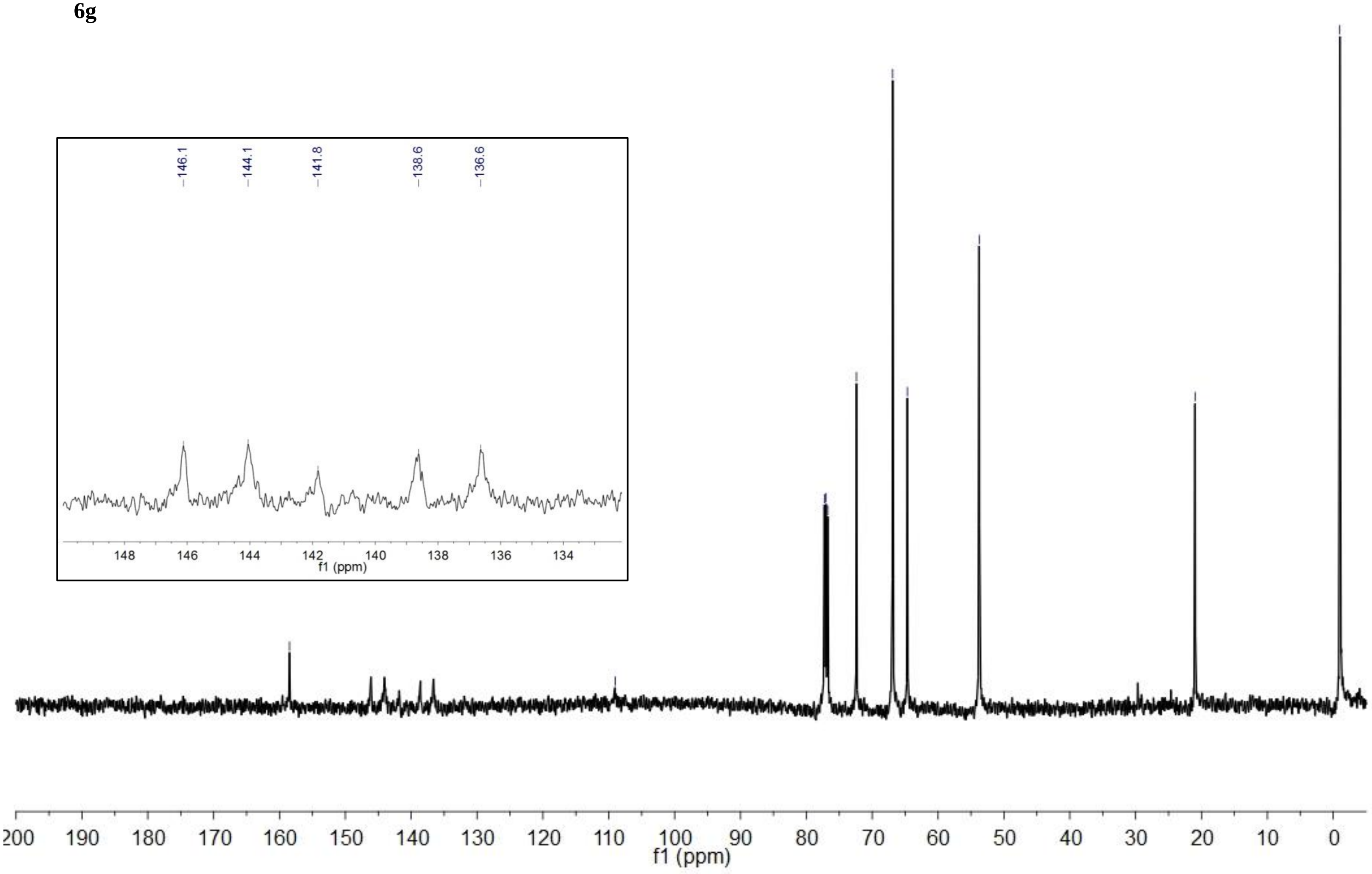
158.5
109.0
77.3
77.0
76.7
72.4
66.9
64.7
53.8
21.0
-1.0
146.1
144.1
141.8
138.6
136.6
148
146
144
142
140
138
136
134
f1 (ppm)
200
190
180
170
160
150
140
130
120
110
100
90
80
70
60
50
40
30
20
10
0
f1 (ppm)

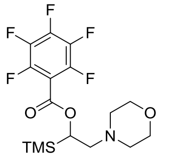
F
F
F
F
F
O
O
O
N
TMS

6h

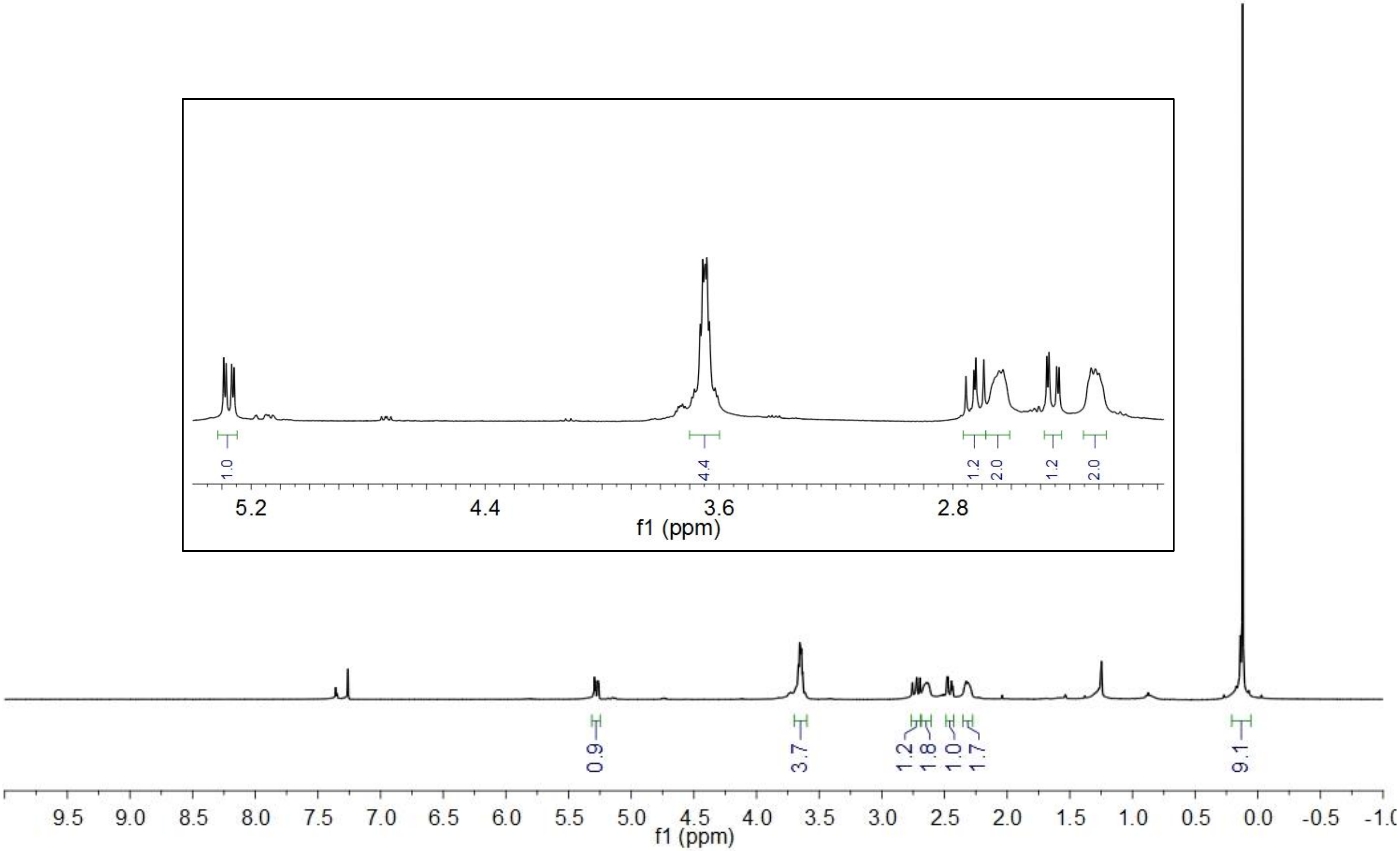
1.0
4.4
1.2
2.0
1.2
2.0
5.2
4.4
3.6
2.8
f1 (ppm)
0.9
3.7
1.2
1.8
1.0
1.7
9.1
9.5
9.0
8.5
8.0
7.5
7.0
6.5
6.0
5.5
5.0
4.5
4.0
3.5
3.0
2.5
2.0
1.5
1.0
0.5
0.0
-0.5
f1 (ppm)

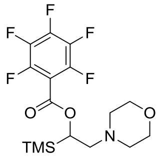

6h

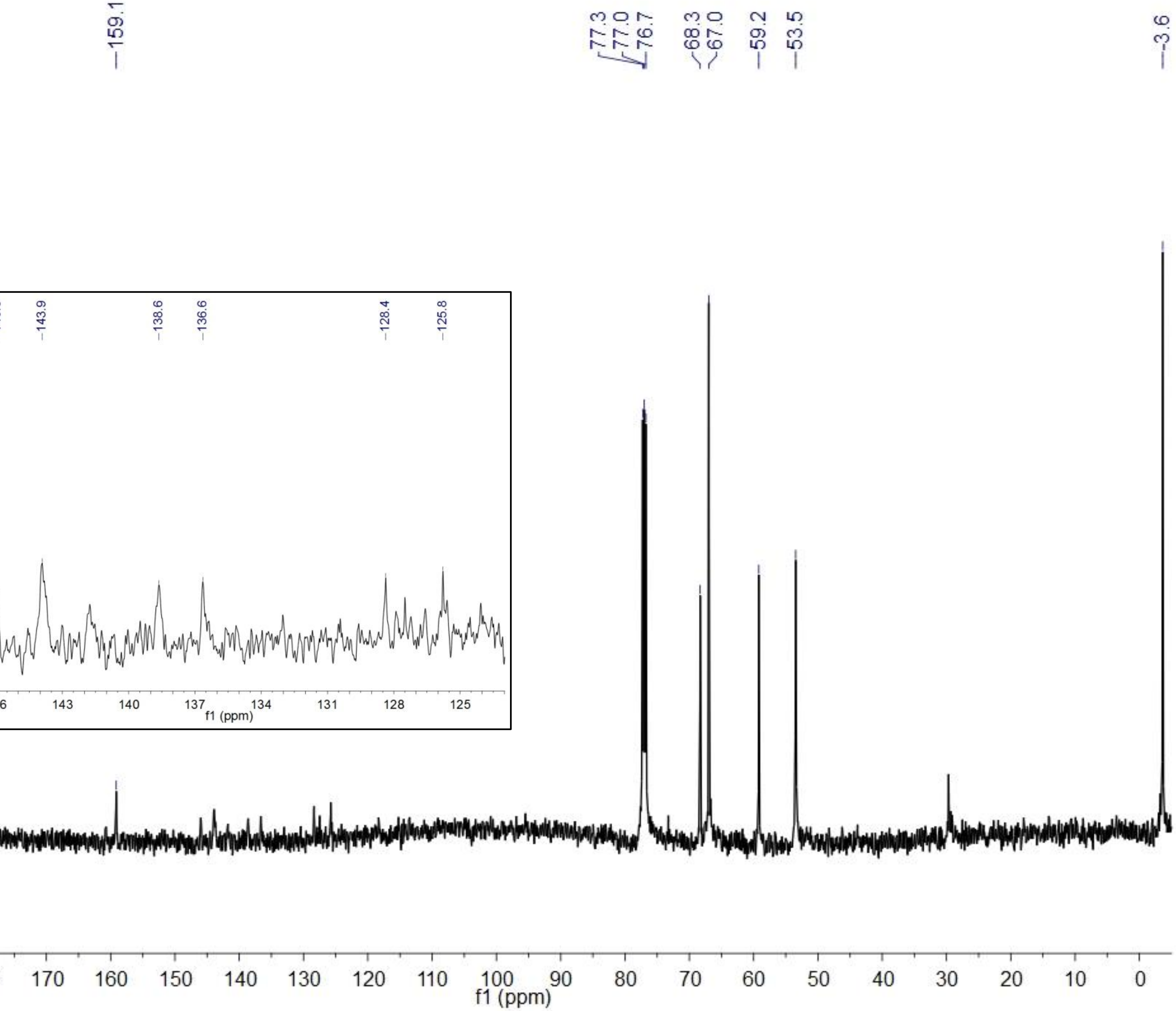

159.1
77.3
77.0
76.7
68.3
67.0
59.2
53.5
-3.6
146.0
143.9
138.6
136.6
128.4
125.8
146
143
140
137
134
131
128
125
f1 (ppm)
190
180
170
160
150
140
130
120
110
100
90
80
70
60
50
40
30
20
10
0
f1 (ppm)

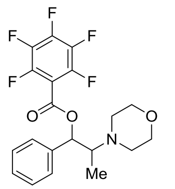

First diastereomer

6i

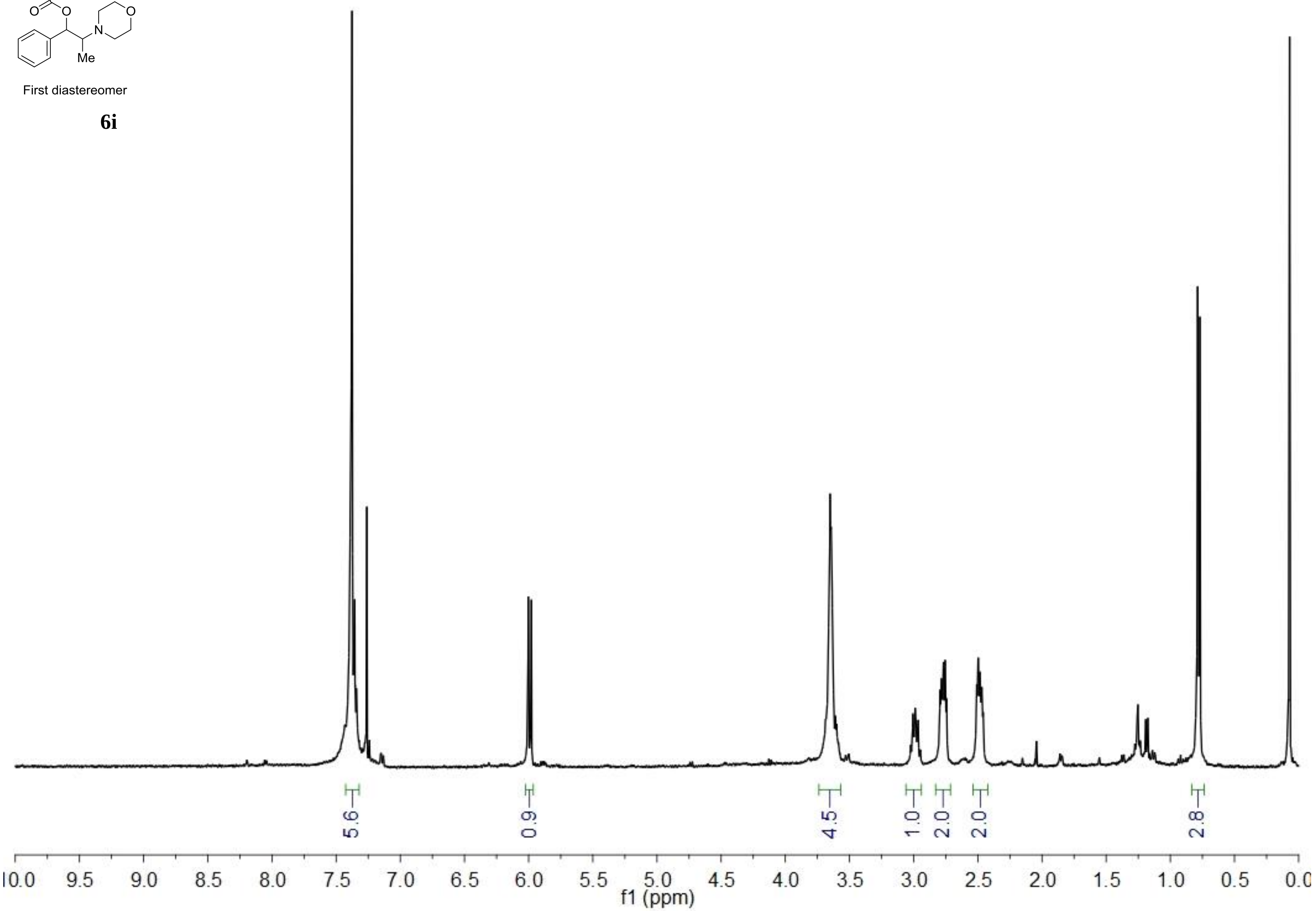

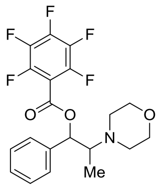

First diastereomer

6i

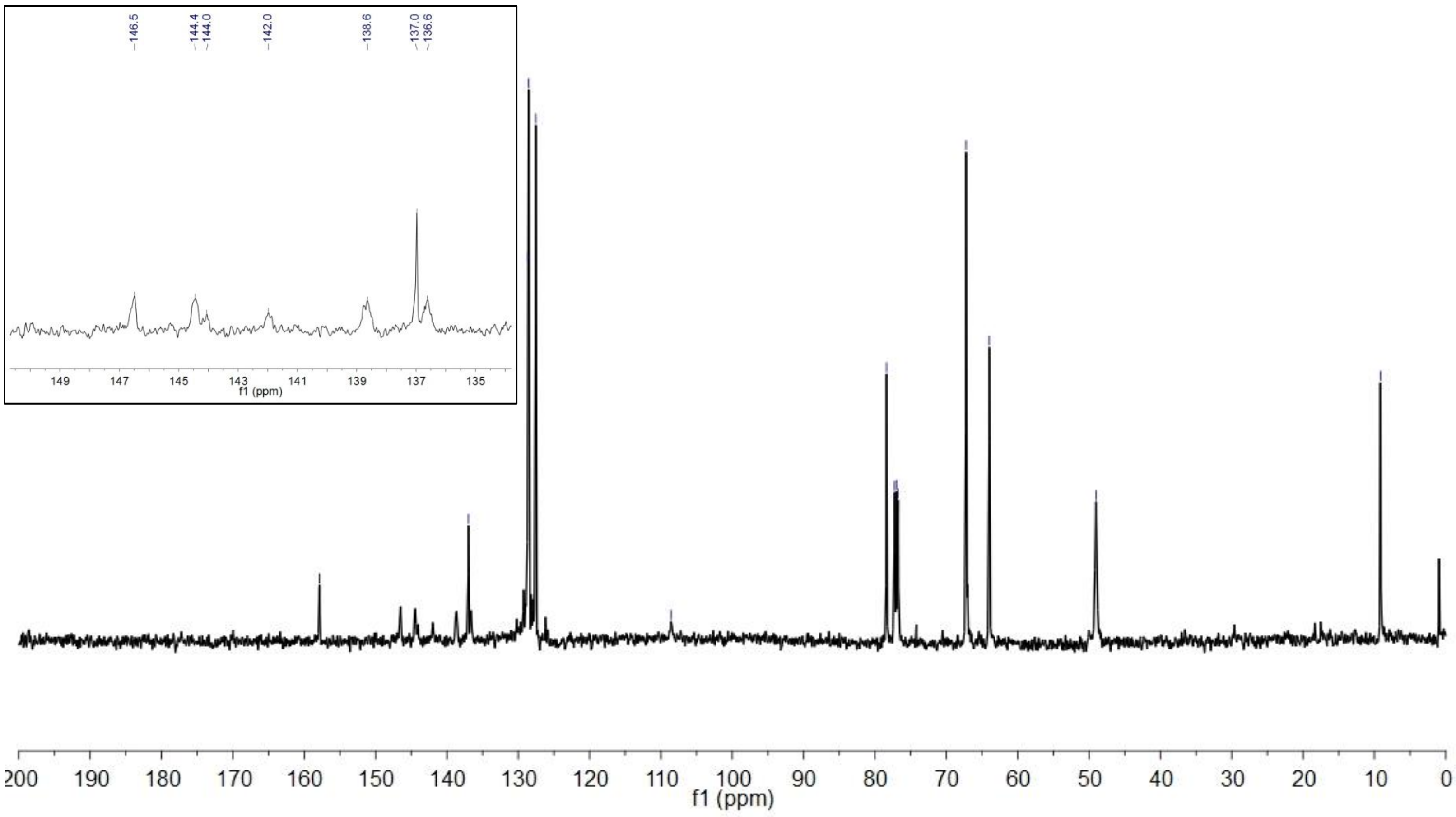

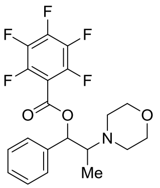

Second diastereomer

6i

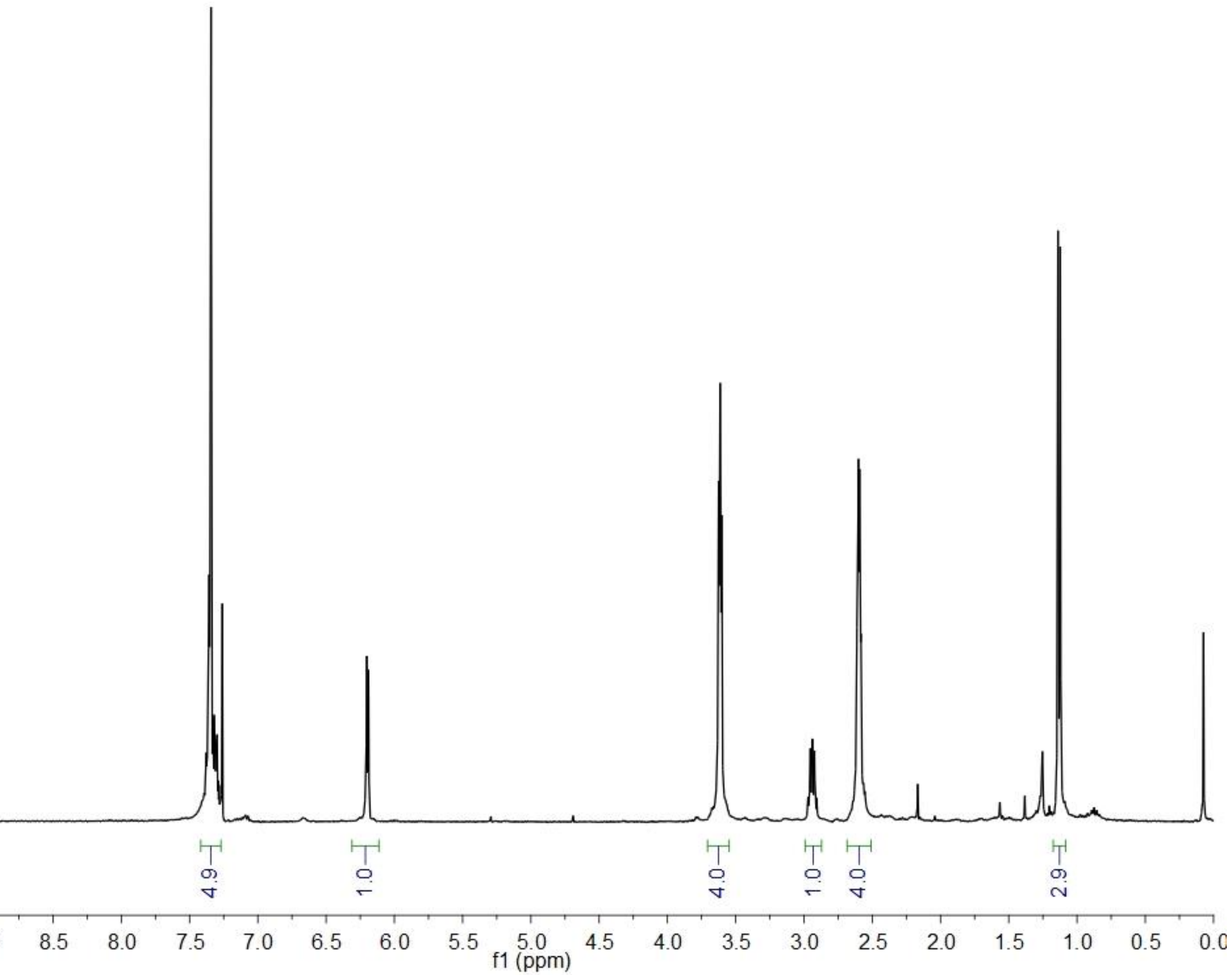

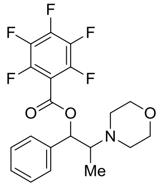

Second diastereomer

6i

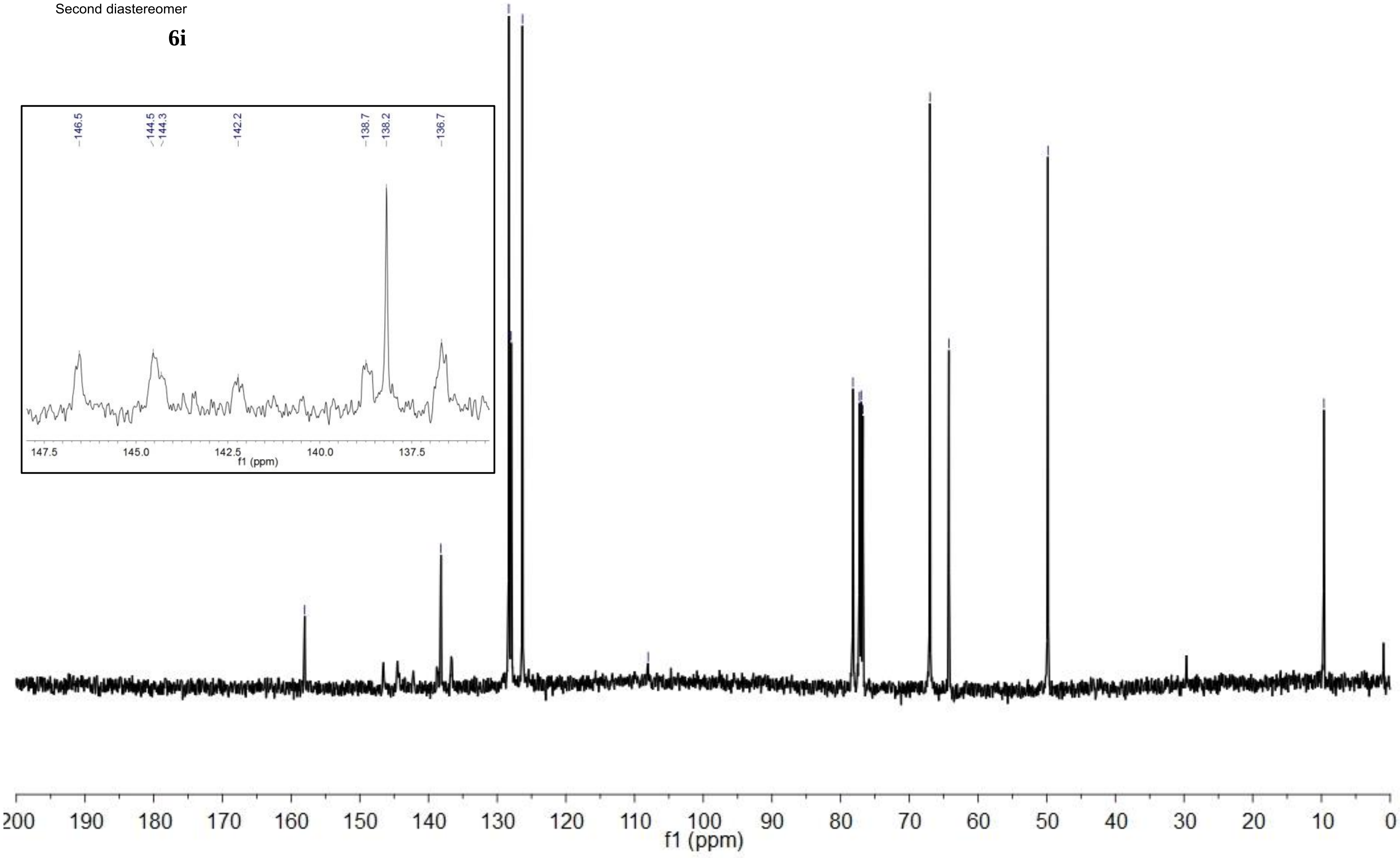

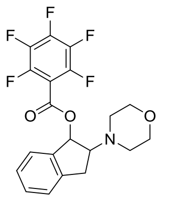

Minor diastereomer

6j

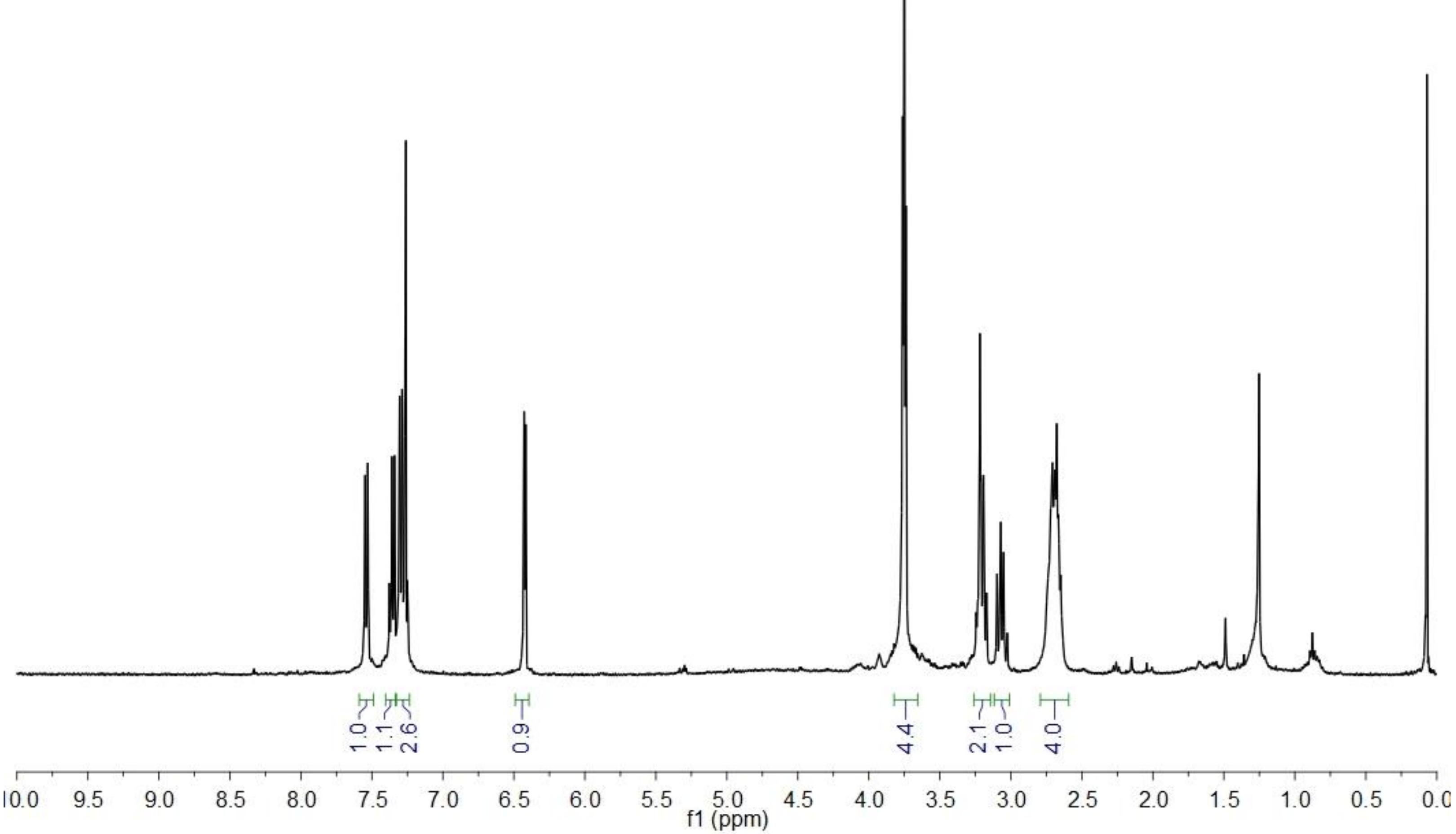

1.0
1.1
2.6
0.9
4.4
2.1
1.0
4.0
10.0 9.5 9.0 8.5 8.0 7.5 7.0 6.5 6.0 5.5 5.0 4.5 4.0 3.5 3.0 2.5 2.0 1.5 1.0 0.5 0.0
f1 (ppm)

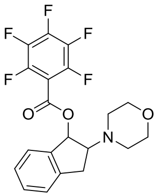

Minor diastereomer

6j

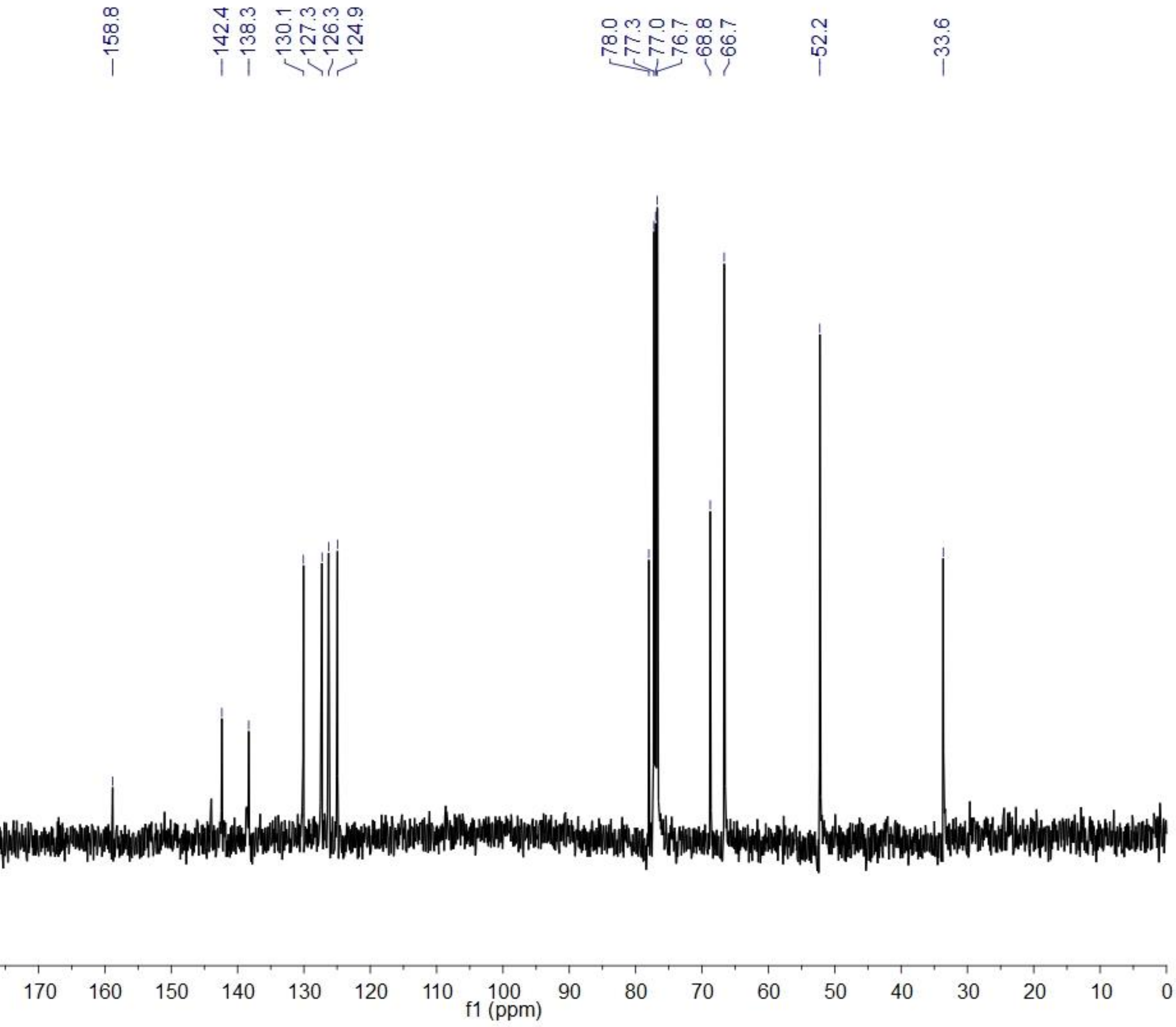

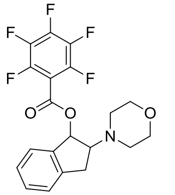

Major diastereomer **6j**

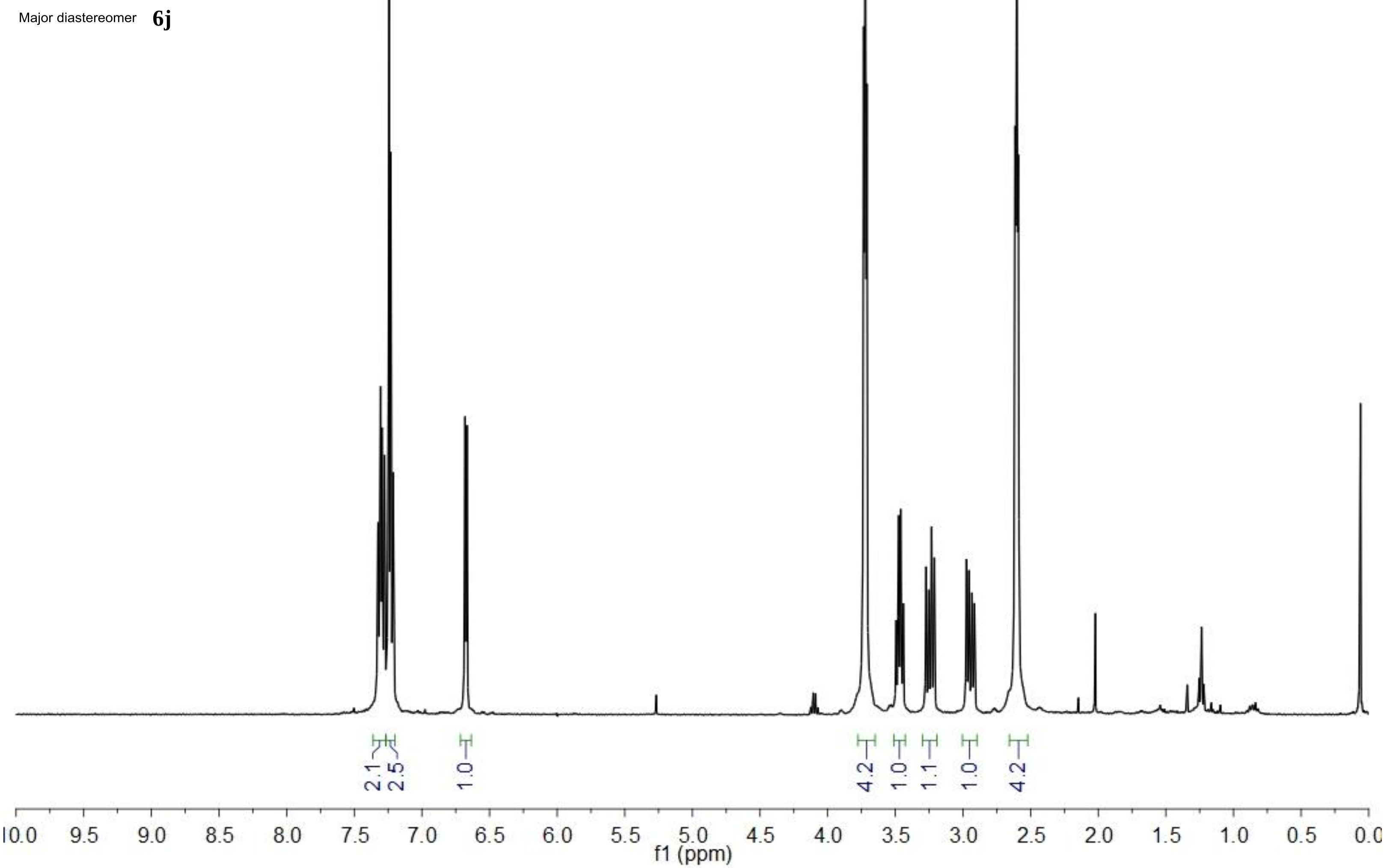

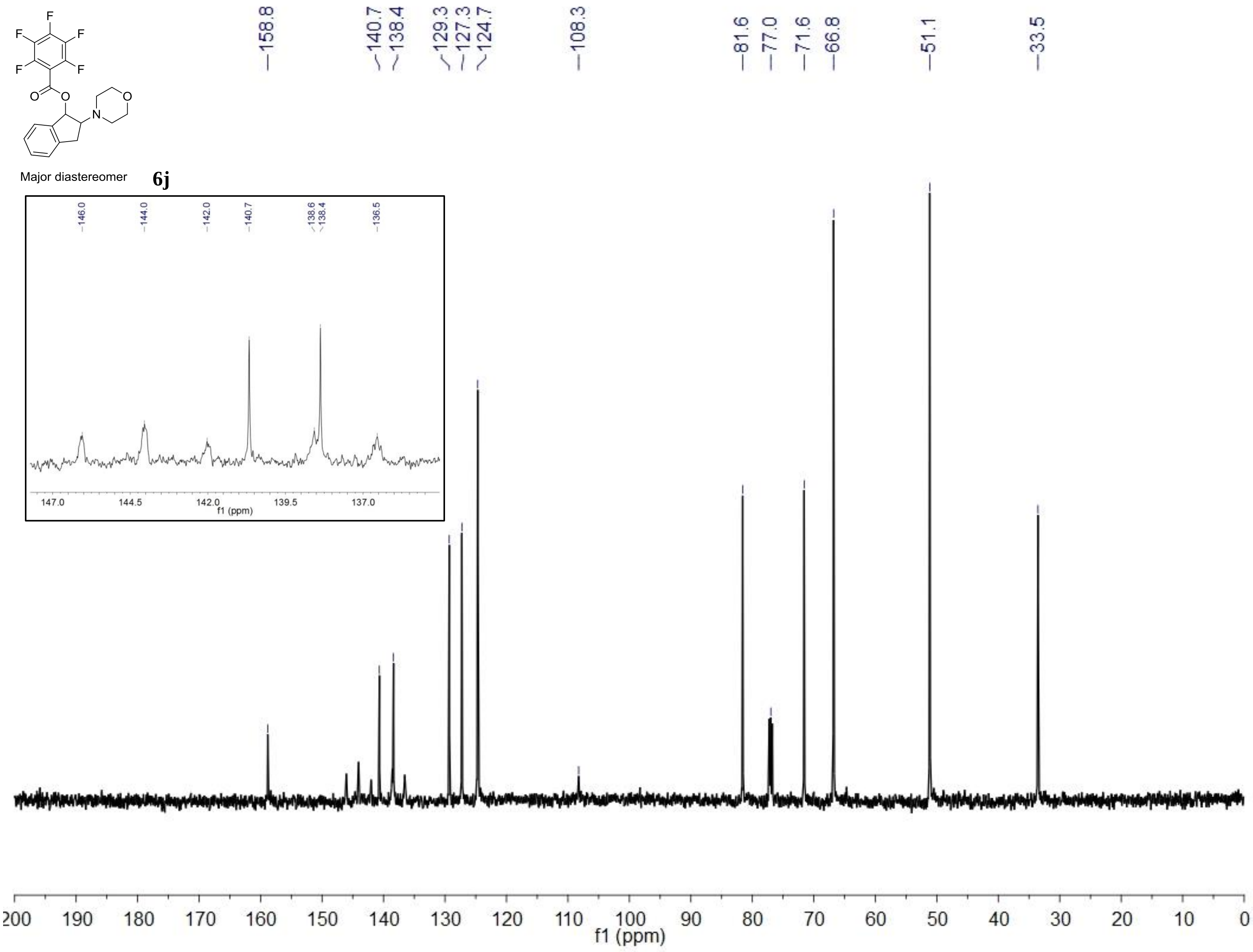

Major diastereomer
6j
158.8
140.7
138.4
129.3
127.3
124.7
108.3
81.6
77.0
71.6
66.8
51.1
33.5
146.0
144.0
142.0
140.7
138.6
138.4
136.5
f1 (ppm)
f1 (ppm)

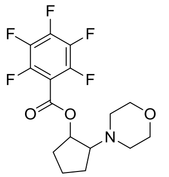

F
F
F
F
F
O
O
N
O

6k

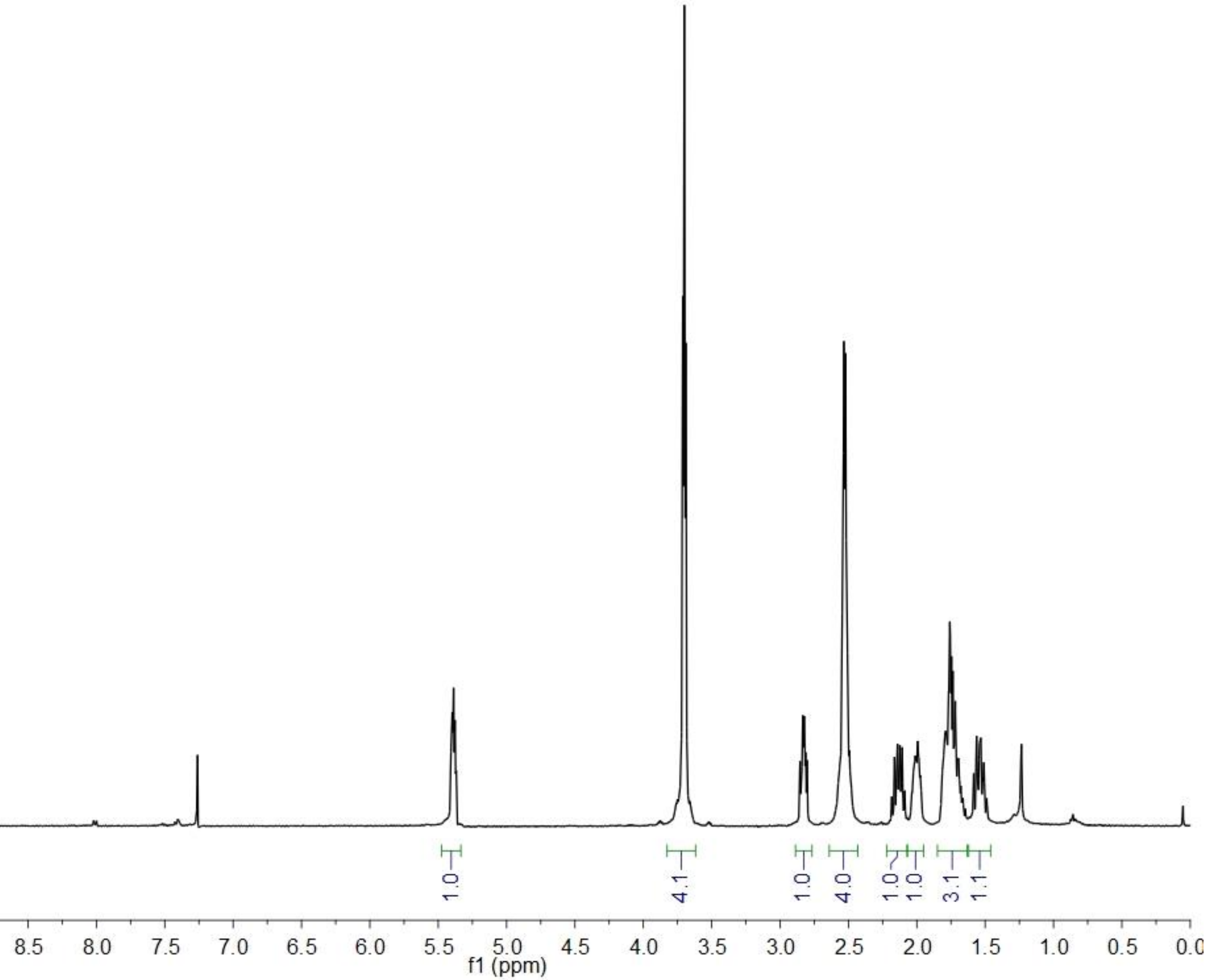

1.0
4.1
1.0
4.0
1.0
1.0
3.1
1.1
10.0
9.5
9.0
8.5
8.0
7.5
7.0
6.5
6.0
5.5
5.0
4.5
4.0
3.5
3.0
2.5
2.0
1.5
1.0
0.5
0.0
f1 (ppm)

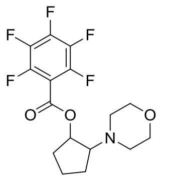
F
F
F
F
F
O
O
N
O

6k

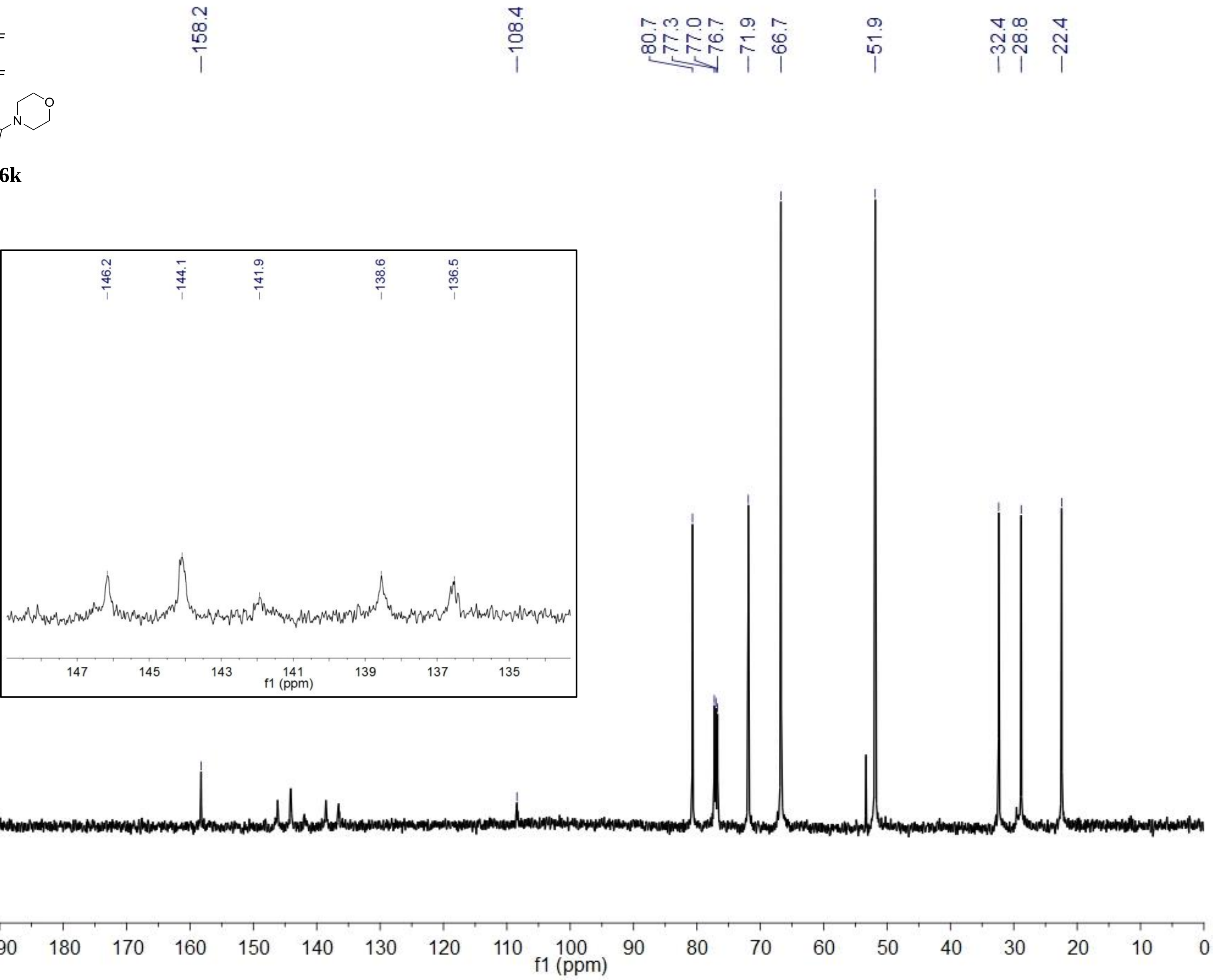
158.2
108.4
80.7
77.3
77.0
76.7
71.9
66.7
51.9
32.4
28.8
22.4
146.2
144.1
141.9
138.6
136.5
147
145
143
141
139
137
135
f1 (ppm)
200
190
180
170
160
150
140
130
120
110
100
90
80
70
60
50
40
30
20
10
0
f1 (ppm)

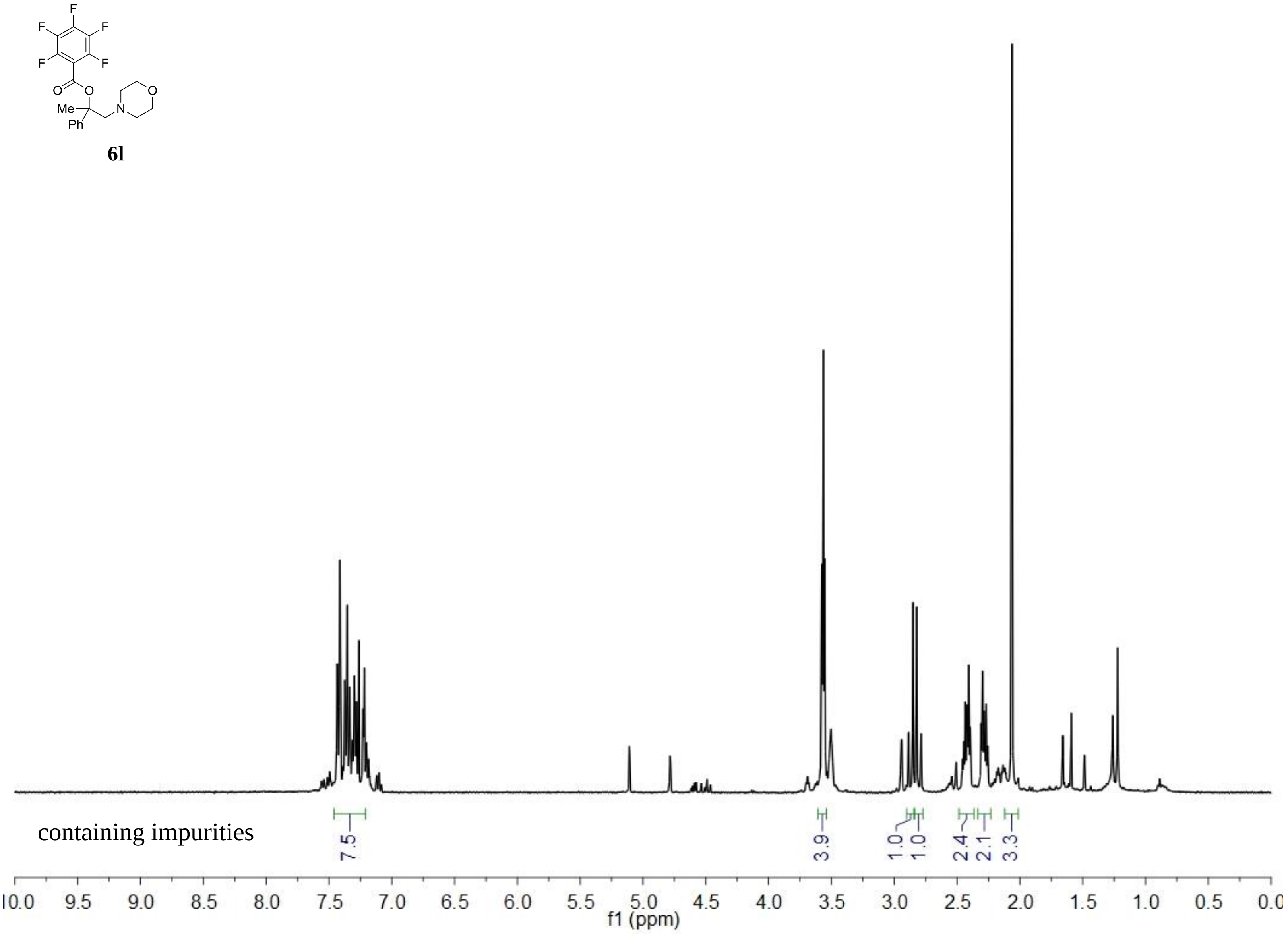
F
F
F
F
F
O
O
Me
Ph
N
O
6l
containing impurities
7.5
3.9
1.0
1.0
2.4
2.1
3.3
10.0 9.5 9.0 8.5 8.0 7.5 7.0 6.5 6.0 5.5 5.0 4.5 4.0 3.5 3.0 2.5 2.0 1.5 1.0 0.5 0.0
f1 (ppm)

VI. X-ray crystallography information

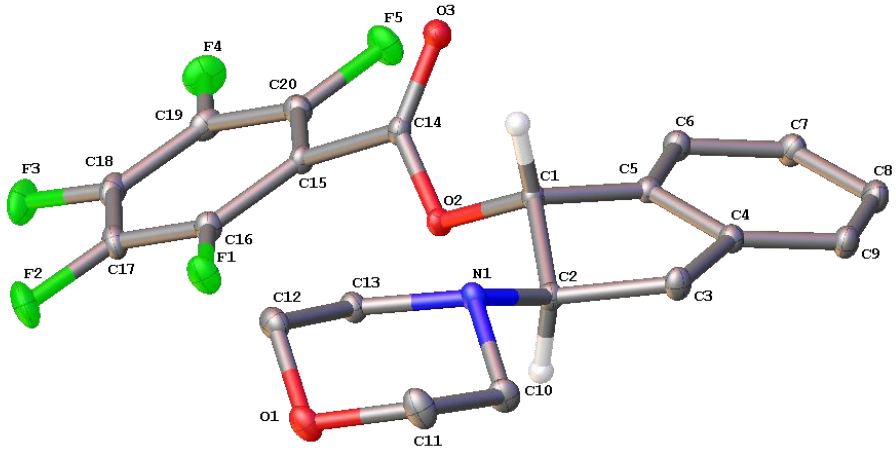

Crystal structure report for rds453 (6j – major diastereomer**)**

A colorless block-like specimen of $C_{20}H_{16}F_5NO_3$, approximate dimensions 0.346 mm × 0.370 mm × 0.509 mm, was used for the X-ray crystallographic analysis. The X-ray intensity data were measured.

The total exposure time was 4.69 hours. The frames were integrated with the Bruker SAINT software package using a narrow-frame algorithm. The integration of the data using a monoclinic unit cell yielded a total of 47250 reflections to a maximum θ angle of 33.36° (0.65 Å resolution), of which 6720 were independent (average redundancy 7.031, completeness = 99.6%, R_{int} = 2.40%, R_{sig} = 1.66%) and 5670(84.38%) were greater than 2σ(F^2). The final cell constants of *a* = 11.9738(5) Å, *b* = 12.0661(5) Å, *c* = 12.2587(5) Å, β = 101.0630(10)°, volume = 1738.19(12) $Å^3$, are based upon the refinement of the XYZ-centroids of 9873 reflections above 20 σ(I) with 4.784° < 2θ < 66.53°. Data were corrected for absorption effects using the multi-scan method (SADABS). The ratio of minimum to maximum apparent transmission was 0.971. The calculated minimum and maximum transmission coefficients (based on crystal size) are 0.6974 and 0.7469.

The final anisotropic full-matrix least-squares refinement on F^2 with 262 variables converged at R1 = 3.46%, for the observed data and wR2 = 10.26% for all data. The goodness-of-fit was 1.038. The largest peak in the final difference electron density synthesis was 0.637 $e^-/Å^3$ and the largest hole was −0.227 $e^-/Å^3$ with an RMS deviation of 0.058 $e^-/Å^3$. On the basis of the final model, the calculated density was1.579 g/cm^3 and F(000), 848 e^-.

Sample and crystal data for rds453.

**Identification code**	rds453	
**Chemical formula**	$C_{20}H_{16}F_5NO_3$	
**Formula weight**	413.34 g/mol	
**Temperature**	100(2) K	
**Wavelength**	0.71073 Å	
**Crystal size**	0.346 × 0.370 × 0.509 mm	
**Crystal habit**	colorless block	
**Crystal system**	monoclinic	
**Space group**	$P12_1/c1$ (No. 14)	
**Unit cell dimensions**	*a* = 11.9738(5) Å	α = 90°
	b = 12.0661(5) Å	β = 101.0630(10)°
	c = 12.2587(5) Å	γ = 90°
**Volume**	1738.19(12) $Å^3$	

**Z**	4
**Density (calculated)**	1.579 g/cm^3
**Absorption coefficient**	0.141 mm^{-1}
**F(000)**	848

Data collection and structure refinement for rds453.

**Theta range for data collection**	1.73 to 33.36°
**Index ranges**	−18<=h<=14, −18<=k<=18, −18<=l<=18
**Reflections collected**	47250
**Independent reflections**	6720 [R(int) = 0.0240]
**Coverage of independent reflections**	99.6%
**Absorption correction**	multi-scan
**Max. and min. transmission**	0.7469 and 0.6974
**Refinement method**	Full-matrix least-squares on F^2
**Refinement program**	SHELXL-2013 (Sheldrick, 2013)
**Function minimized**	Σ w(F_o^2 - F_c^2)^2
**Data / restraints / parameters**	6720 / 0 / 262
**Goodness-of-fit on F^2**	1.038
**Δ/σ_{max}**	0.001
**Final R indices**	5670 data; I>2σ(I) R1 = 0.0346, wR2 = 0.0957
	all data R1 = 0.0436, wR2 = 0.1026
**Weighting scheme**	w=1/[σ^2(F_o^2)+(0.0564P)^2+0.5043P] where P=(F_o^2+2F_c^2)/3
**Largest diff. peak and hole**	0.637 and -0.227 eÅ^{-3}
**R.M.S. deviation from mean**	0.058 eÅ^{-3}

Atomic coordinates and equivalent isotropic atomic displacement parameters (Å^2) for rds453.

U(eq) is defined as one third of the trace of the orthogonalized U_{ij} tensor.

	x/a	y/b	z/c	U(eq)
F1	0.04504(5)	0.10427(5)	0.97906(5)	0.02179(12)
F2	0.94089(5)	0.06547(5)	0.76558(5)	0.02235(12)
F3	0.80568(5)	0.22014(4)	0.65120(4)	0.01703(11)
F4	0.02007(5)	0.30270(5)	0.07534(4)	0.02104(12)
F5	0.88609(5)	0.45926(5)	0.96253(4)	0.01933(11)
O1	0.76857(5)	0.45042(5)	0.64236(5)	0.01094(10)
O2	0.69509(5)	0.47918(5)	0.79657(5)	0.01384(11)
O3	0.58104(6)	0.24496(5)	0.26060(5)	0.01666(12)
N1	0.60389(6)	0.43798(6)	0.40174(5)	0.01128(12)
C1	0.98112(7)	0.18267(7)	0.92166(7)	0.01455(14)
C2	0.92766(7)	0.16319(7)	0.81288(7)	0.01431(14)
C3	0.85910(7)	0.24426(7)	0.75439(6)	0.01218(13)
C4	0.84174(7)	0.34581(6)	0.80266(6)	0.01067(13)
C5	0.75944(7)	0.43169(6)	0.74772(6)	0.01069(13)
C6	0.68792(6)	0.53215(6)	0.58468(6)	0.00990(12)
C7	0.72747(6)	0.65000(6)	0.60989(6)	0.01028(13)
C8	0.78486(7)	0.69713(7)	0.70825(6)	0.01269(13)
C9	0.81179(7)	0.80998(7)	0.70900(7)	0.01466(14)

C10	0.78075(8)	0.87382(7)	0.61348(7)	0.01533(15)
C11	0.72383(7)	0.82597(7)	0.51443(7)	0.01427(14)
C12	0.69813(7)	0.71342(6)	0.51360(6)	0.01142(13)
C13	0.64106(7)	0.64194(7)	0.41857(6)	0.01295(14)
C14	0.67985(6)	0.52410(6)	0.45813(6)	0.01046(13)
C15	0.64182(7)	0.32733(7)	0.44311(6)	0.01254(13)
C16	0.57296(7)	0.23687(7)	0.37508(7)	0.01525(14)
C17	0.53610(8)	0.34961(7)	0.21967(7)	0.01788(16)
C18	0.60319(8)	0.44422(7)	0.28154(7)	0.01483(14)
C19	0.89789(7)	0.36326(7)	0.91179(6)	0.01265(13)
C20	0.96736(7)	0.28346(7)	0.97098(7)	0.01423(14)

Bond lengths (Å) for rds453.

F1-C1	1.3301(9)	F2-C2	1.3368(10)
F3-C3	1.3355(9)	F4-C20	1.3338(9)
F5-C19	1.3351(9)	O1-C5	1.3362(9)
O1-C6	1.4638(9)	O2-C5	1.2074(10)
O3-C17	1.4256(11)	O3-C16	1.4284(11)
N1-C14	1.4641(10)	N1-C15	1.4689(10)
N1-C18	1.4739(10)	C1-C20	1.3819(12)
C1-C2	1.3841(12)	C2-C3	1.3852(11)
C3-C4	1.3936(11)	C4-C19	1.3932(11)
C4-C5	1.4979(11)	C6-C7	1.5118(11)
C6-C14	1.5389(10)	C6-H6	1.0
C7-C8	1.3898(11)	C7-C12	1.3940(11)
C8-C9	1.3989(11)	C8-H8	0.95
C9-C10	1.3912(12)	C9-H9	0.95
C10-C11	1.3980(12)	C10-H10	0.95
C11-C12	1.3921(11)	C11-H11	0.95
C12-C13	1.5041(11)	C13-C14	1.5447(11)
C13-H13A	0.99	C13-H13B	0.99
C14-H14	1.0	C15-C16	1.5174(11)
C15-H15A	0.99	C15-H15B	0.99
C16-H16A	0.99	C16-H16B	0.99
C17-C18	1.5133(12)	C17-H17A	0.99
C17-H17B	0.99	C18-H18A	0.99
C18-H18B	0.99	C19-C20	1.3834(11)

Bond angles (°) for rds453.

C5-O1-C6	114.33(6)	C17-O3-C16	108.31(6)
C14-N1-C15	111.05(6)	C14-N1-C18	108.64(6)
C15-N1-C18	109.49(6)	F1-C1-C20	119.96(8)
F1-C1-C2	120.11(8)	C20-C1-C2	119.94(7)
F2-C2-C1	119.76(7)	F2-C2-C3	120.41(7)
C1-C2-C3	119.82(8)	F3-C3-C2	117.71(7)
F3-C3-C4	120.69(7)	C2-C3-C4	121.54(7)
C19-C4-C3	117.17(7)	C19-C4-C5	118.81(7)
C3-C4-C5	123.80(7)	O2-C5-O1	125.28(7)
O2-C5-C4	122.19(7)	O1-C5-C4	112.51(6)
O1-C6-C7	112.53(6)	O1-C6-C14	110.63(6)
C7-C6-C14	102.83(6)	O1-C6-H6	110.2
C7-C6-H6	110.2	C14-C6-H6	110.2

C8-C7-C12	120.82(7)	C8-C7-C6	130.21(7)
C12-C7-C6	108.97(6)	C7-C8-C9	118.62(7)
C7-C8-H8	120.7	C9-C8-H8	120.7
C10-C9-C8	120.62(8)	C10-C9-H9	119.7
C8-C9-H9	119.7	C9-C10-C11	120.62(8)
C9-C10-H10	119.7	C11-C10-H10	119.7
C12-C11-C10	118.65(7)	C12-C11-H11	120.7
C10-C11-H11	120.7	C11-C12-C7	120.66(7)
C11-C12-C13	129.20(7)	C7-C12-C13	110.14(7)
C12-C13-C14	102.71(6)	C12-C13-H13A	111.2
C14-C13-H13A	111.2	C12-C13-H13B	111.2
C14-C13-H13B	111.2	H13A-C13-H13B	109.1
N1-C14-C6	115.72(6)	N1-C14-C13	112.52(6)
C6-C14-C13	102.43(6)	N1-C14-H14	108.6
C6-C14-H14	108.6	C13-C14-H14	108.6
N1-C15-C16	111.39(7)	N1-C15-H15A	109.4
C16-C15-H15A	109.4	N1-C15-H15B	109.4
C16-C15-H15B	109.4	H15A-C15-H15B	108.0
O3-C16-C15	111.02(7)	O3-C16-H16A	109.4
C15-C16-H16A	109.4	O3-C16-H16B	109.4
C15-C16-H16B	109.4	H16A-C16-H16B	108.0
O3-C17-C18	111.32(7)	O3-C17-H17A	109.4
C18-C17-H17A	109.4	O3-C17-H17B	109.4
C18-C17-H17B	109.4	H17A-C17-H17B	108.0
N1-C18-C17	111.29(7)	N1-C18-H18A	109.4
C17-C18-H18A	109.4	N1-C18-H18B	109.4
C17-C18-H18B	109.4	H18A-C18-H18B	108.0
F5-C19-C20	117.80(7)	F5-C19-C4	120.25(7)
C20-C19-C4	121.96(7)	F4-C20-C1	119.90(7)
F4-C20-C19	120.54(8)	C1-C20-C19	119.55(7)

Torsion angles (°) for rds453.

F1-C1-C2-F2	-0.28(12)	C20-C1-C2-F2	-179.80(8)
F1-C1-C2-C3	178.52(7)	C20-C1-C2-C3	-0.99(13)
F2-C2-C3-F3	1.39(12)	C1-C2-C3-F3	-177.40(7)
F2-C2-C3-C4	178.46(7)	C1-C2-C3-C4	-0.34(13)
F3-C3-C4-C19	178.07(7)	C2-C3-C4-C19	1.10(12)
F3-C3-C4-C5	3.52(12)	C2-C3-C4-C5	-173.46(7)
C6-O1-C5-O2	-3.07(11)	C6-O1-C5-C4	178.74(6)
C19-C4-C5-O2	-39.97(11)	C3-C4-C5-O2	134.50(9)
C19-C4-C5-O1	138.29(7)	C3-C4-C5-O1	-47.24(10)
C5-O1-C6-C7	82.08(8)	C5-O1-C6-C14	-163.52(6)
O1-C6-C7-C8	-37.28(11)	C14-C6-C7-C8	-156.34(8)
O1-C6-C7-C12	142.17(6)	C14-C6-C7-C12	23.11(8)
C12-C7-C8-C9	0.57(12)	C6-C7-C8-C9	179.96(8)
C7-C8-C9-C10	0.60(12)	C8-C9-C10-C11	-1.13(13)
C9-C10-C11-C12	0.48(13)	C10-C11-C12-C7	0.69(12)
C10-C11-C12-C13	-178.17(8)	C8-C7-C12-C11	-1.23(12)
C6-C7-C12-C11	179.26(7)	C8-C7-C12-C13	177.83(7)
C6-C7-C12-C13	-1.67(9)	C11-C12-C13-C14	158.60(8)
C7-C12-C13-C14	-20.36(8)	C15-N1-C14-C6	-61.44(8)
C18-N1-C14-C6	178.09(6)	C15-N1-C14-C13	-178.72(6)

C18-N1-C14-C13	60.81(8)	O1-C6-C14-N1	82.47(8)
C7-C6-C14-N1	-157.16(6)	O1-C6-C14-C13	-154.75(6)
C7-C6-C14-C13	-34.37(7)	C12-C13-C14-N1	158.27(6)
C12-C13-C14-C6	33.36(7)	C14-N1-C15-C16	-172.61(6)
C18-N1-C15-C16	-52.64(8)	C17-O3-C16-C15	-61.14(9)
N1-C15-C16-O3	58.25(9)	C16-O3-C17-C18	61.24(9)
C14-N1-C18-C17	173.94(7)	C15-N1-C18-C17	52.50(9)
O3-C17-C18-N1	-58.11(9)	C3-C4-C19-F5	179.17(7)
C5-C4-C19-F5	-5.99(11)	C3-C4-C19-C20	-0.56(12)
C5-C4-C19-C20	174.27(7)	F1-C1-C20-F4	1.34(12)
C2-C1-C20-F4	-179.14(7)	F1-C1-C20-C19	-178.00(7)
C2-C1-C20-C19	1.51(12)	F5-C19-C20-F4	0.19(12)
C4-C19-C20-F4	179.93(7)	F5-C19-C20-C1	179.53(7)
C4-C19-C20-C1	-0.73(12)		

Anisotropic atomic displacement parameters (Å^2) for rds453.

The anisotropic atomic displacement factor exponent takes the form: $-2\pi^2[h^2 a^{*2} U_{11} + ... + 2 h k a^* b^* U_{12}]$

	U_{11}	U_{22}	U_{33}	U_{23}	U_{13}	U_{12}
F1	0.0174(2)	0.0189(3)	0.0267(3)	0.0113(2)	-0.0018(2)	0.0042(2)
F2	0.0247(3)	0.0111(2)	0.0289(3)	-0.0034(2)	-0.0007(2)	0.0054(2)
F3	0.0207(3)	0.0159(2)	0.0122(2)	-0.00424(17)	-0.00281(18)	0.00342(19)
F4	0.0194(3)	0.0294(3)	0.0112(2)	0.0016(2)	-0.00476(19)	-0.0002(2)
F5	0.0229(3)	0.0172(2)	0.0158(2)	-0.00703(19)	-0.0018(2)	0.0018(2)
O1	0.0123(2)	0.0110(2)	0.0091(2)	0.00179(18)	0.00074(18)	0.00265(19)
O2	0.0167(3)	0.0120(2)	0.0135(3)	0.00036(19)	0.0045(2)	0.0016(2)
O3	0.0193(3)	0.0144(3)	0.0147(3)	-0.0042(2)	-0.0006(2)	0.0030(2)
N1	0.0119(3)	0.0105(3)	0.0104(3)	-0.0017(2)	-0.0005(2)	0.0003(2)
C1	0.0111(3)	0.0139(3)	0.0176(3)	0.0063(3)	0.0001(3)	0.0011(3)
C2	0.0141(3)	0.0097(3)	0.0184(4)	0.0003(3)	0.0014(3)	0.0014(3)
C3	0.0133(3)	0.0116(3)	0.0107(3)	-0.0003(2)	-0.0001(2)	-0.0001(2)
C4	0.0119(3)	0.0096(3)	0.0099(3)	0.0009(2)	0.0006(2)	0.0002(2)
C5	0.0124(3)	0.0089(3)	0.0102(3)	0.0007(2)	0.0006(2)	-0.0009(2)
C6	0.0106(3)	0.0091(3)	0.0094(3)	0.0007(2)	0.0002(2)	0.0010(2)
C7	0.0108(3)	0.0097(3)	0.0102(3)	0.0001(2)	0.0016(2)	0.0004(2)
C8	0.0152(3)	0.0114(3)	0.0110(3)	-0.0003(2)	0.0013(3)	-0.0012(3)
C9	0.0186(4)	0.0125(3)	0.0126(3)	-0.0020(3)	0.0022(3)	-0.0029(3)
C10	0.0199(4)	0.0111(3)	0.0153(3)	-0.0005(3)	0.0040(3)	-0.0018(3)
C11	0.0182(4)	0.0111(3)	0.0134(3)	0.0017(3)	0.0026(3)	-0.0006(3)
C12	0.0124(3)	0.0105(3)	0.0109(3)	0.0003(2)	0.0012(2)	0.0001(2)
C13	0.0159(3)	0.0114(3)	0.0103(3)	0.0012(2)	-0.0007(3)	0.0005(3)
C14	0.0112(3)	0.0103(3)	0.0092(3)	-0.0003(2)	0.0004(2)	0.0002(2)
C15	0.0135(3)	0.0111(3)	0.0123(3)	-0.0012(2)	0.0007(3)	0.0004(3)
C16	0.0156(3)	0.0126(3)	0.0172(3)	-0.0035(3)	0.0023(3)	-0.0009(3)
C17	0.0200(4)	0.0161(4)	0.0145(3)	-0.0036(3)	-0.0043(3)	0.0033(3)
C18	0.0182(4)	0.0150(3)	0.0099(3)	-0.0011(3)	-0.0008(3)	0.0006(3)
C19	0.0137(3)	0.0126(3)	0.0111(3)	-0.0010(2)	0.0008(2)	-0.0008(3)
C20	0.0126(3)	0.0182(4)	0.0105(3)	0.0029(3)	-0.0013(3)	-0.0012(3)

Hydrogen atomic coordinates and isotropic atomic displacement parameters (Å^2) for rds453.

	**x/a**	**y/b**	**z/c**	**U(eq)**
H6	0.6112	0.5209	0.6039	0.012
H8	0.8054	0.6536	0.7736	0.015
H9	0.8516	0.8433	0.7754	0.018
H10	0.7984	0.9506	0.6156	0.018
H11	0.7031	0.8694	0.4490	0.017
H13A	0.6669	0.6612	0.3490	0.016
H13B	0.5572	0.6491	0.4066	0.016
H14	0.7579	0.5112	0.4428	0.013
H15A	0.6339	0.3204	0.5217	0.015
H15B	0.7233	0.3180	0.4399	0.015
H16A	0.6013	0.1635	0.4042	0.018
H16B	0.4922	0.2432	0.3822	0.018
H17A	0.4557	0.3551	0.2280	0.021
H17B	0.5383	0.3554	0.1395	0.021
H18A	0.6824	0.4417	0.2690	0.018
H18B	0.5692	0.5156	0.2521	0.018
